# Lineage-tracing hematopoietic stem cell origins *in vivo* to efficiently make human HLF+ HOXA+ hematopoietic progenitors from pluripotent stem cells

**DOI:** 10.1016/j.devcel.2024.03.003

**Published:** 2024-05-06

**Authors:** Jonas L. Fowler, Sherry Li Zheng, Alana Nguyen, Angela Chen, Xiaochen Xiong, Timothy Chai, Julie Y. Chen, Daiki Karigane, Allison M. Banuelos, Kouta Niizuma, Kensuke Kayamori, Toshinobu Nishimura, M. Kyle Cromer, David Gonzalez-Perez, Charlotte Mason, Daniel Dan Liu, Leyla Yilmaz, Lucile Miquerol, Matthew H. Porteus, Vincent C. Luca, Ravindra Majeti, Hiromitsu Nakauchi, Kristy Red-Horse, Irving L. Weissman, Lay Teng Ang, Kyle M. Loh

**Affiliations:** 1 Institute for Stem Cell Biology & Regenerative Medicine, Stanford University, Stanford, CA 94305, USA; 2 Department of Developmental Biology, Stanford University, Stanford, CA 94305, USA; 3 Division of Hematology, Department of Medicine, Stanford University, Stanford, CA 94305, USA; 4 Department of Pathology, Stanford University, Stanford, CA 94305, USA; 5 Department of Genetics, Stanford University, Stanford, CA 94305, USA; 6 Department of Surgery, University of California, San Francisco, San Francisco, CA 94143, USA; 7 Department of Drug Discovery, Moffitt Cancer Center, Tampa, FL 33612, USA; 8 Aix-Marseille Université, CNRS UMR 7288, IBDM, Marseille 13288, France; 9 Department of Pediatrics, Stanford University, Stanford, CA 94305, USA; 10 Department of Biology, Howard Hughes Medical Institute, Stanford University, Stanford, CA 94305, USA

**Keywords:** hematopoietic stem cell, artery, human pluripotent stem cell differentiation, developmental biology

## Abstract

The developmental origin of blood-forming hematopoietic stem cells (HSCs) is a longstanding question. Here, our non-invasive genetic lineage tracing in mouse embryos pinpoints that artery endothelial cells generate HSCs. Arteries are transiently competent to generate HSCs for 2.5 days (∼E8.5–E11) but subsequently cease, delimiting a narrow time frame for HSC formation *in vivo*. Guided by the arterial origins of blood, we efficiently and rapidly differentiate human pluripotent stem cells (hPSCs) into posterior primitive streak, lateral mesoderm, artery endothelium, hemogenic endothelium, and >90% pure hematopoietic progenitors within 10 days. hPSC-derived hematopoietic progenitors generate T, B, NK, erythroid, and myeloid cells *in vitro* and, critically, express hallmark HSC transcription factors *HLF* and *HOXA5-HOXA10*, which were previously challenging to upregulate. We differentiated hPSCs into highly enriched *HLF*+ *HOXA*+ hematopoietic progenitors with near-stoichiometric efficiency by blocking formation of unwanted lineages at each differentiation step. hPSC-derived *HLF*+ *HOXA*+ hematopoietic progenitors could avail both basic research and cellular therapies.

## Highlights

• Lineage tracing demonstrates that artery endothelial cells (ECs) form HSCs *in vivo*
• Stepwise hPSC differentiation into artery ECs and then hematopoietic progenitors
• Rapid and efficient generation of >90% pure hematopoietic progenitors in 10 days
• hPSC-derived hematopoietic progenitors express core HSC markers *HLF* and *HOXA5-10*

## Graphical abstract

**Figure:**
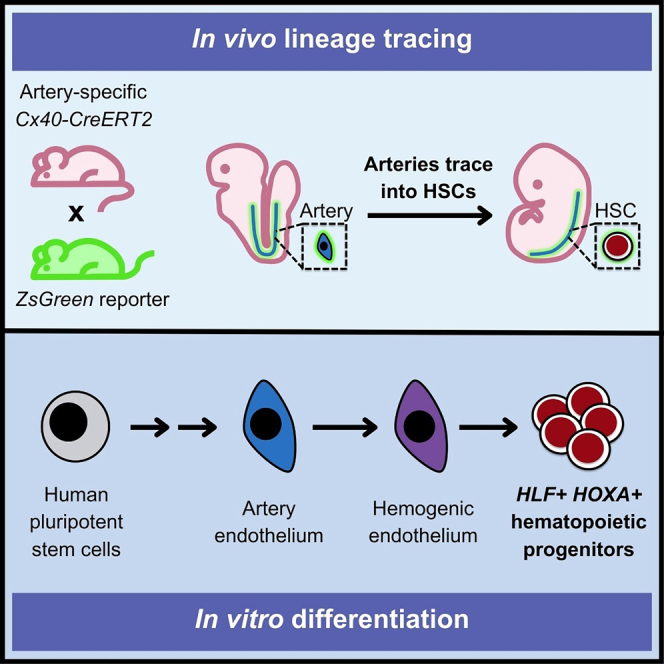

## Teaser

Fowler and Zheng et al. show that artery endothelial cells give rise to hematopoietic stem cells in the mouse embryo. Leveraging this knowledge, they differentiate human pluripotent stem cells into artery endothelial cells and, subsequently, hematopoietic progenitors that express *HLF* and *HOXA5-10*, which are hallmark hematopoietic stem cell transcription factors.

## Introduction

The ability to differentiate human pluripotent stem cells (hPSCs) into hematopoietic progenitors provides a foundation to manufacture massive numbers of human blood and immune cells, availing research and therapies.^1^^,^^2^^,^^3^^,^^4^^,^^5^^,^^6^ However, hPSCs can generate thousands of cell-types through a sequence of branching lineage decisions.^7^ At each step of differentiation, pluripotent cells can stray from the intended lineage path, inadvertently generating non-blood cells. Generating pure populations of hematopoietic progenitors *in vitro* thus remains challenging. To meet this challenge, we (1) revisited the developmental origins of hematopoietic stem cells (HSCs) *in vivo* and (2) identified extracellular signals that must be turned on or off at each step of differentiation from pluripotency to hematopoietic progenitors *in vitro*.

Multiple models have been proposed regarding the embryonic origins of blood-forming HSCs—which generate all blood and immune cells throughout life—and important questions remain.^8^^,^^9^^,^^10^^,^^11^^,^^12^^,^^13^^,^^14^ One model posits that blood and endothelial cells (ECs) largely arise from independent precursors, on the account of single-cell lineage analyses in zebrafish,^15^ mouse,^16^ and chicken.^17^ The second model, introduced by Florence Sabin in 1917, suggests that ECs (known as “hemogenic endothelium”) generate blood.^18^^,^^19^^,^^20^ Live imaging,^21^^,^^22^^,^^23^^,^^24^^,^^25^^,^^26^ staining of fixed sections,^27^ and single-cell RNA sequencing (scRNA-seq)^28^^,^^29^^,^^30^^,^^31^^,^^32^^,^^33^ have revealed that ECs generate cells that express HSC markers. Nevertheless, it is experimentally challenging to test whether the emergent cells are, strictly speaking, functional HSCs capable of engrafting mice and generating all blood and immune cell-types.

Lineage tracing has the capacity to definitively identify the cell-type that generates HSCs. In fact, an elegant lineage-tracing study showed that *VE-Cadherin* (*Cdh5/CD144*)-expressing cells—which were putatively designated as ECs—generate functional HSCs *in vivo*.^34^ However, interpretation of this result is complicated by the fact that VE-Cadherin is expressed by both ECs and embryonic HSCs.^35^^,^^36^ We previously showed that *VE-Cadherin-CreER* directly labels embryonic HSCs,^37^ leaving open the question of whether ECs form functional HSCs within mammalian embryos.

If ECs form HSCs, an intimately related question is what endothelial subtype—such as artery, vein, capillary, or lymphatic EC^38^^,^^39^^,^^40^—generates HSCs. Embryonic tissue fragments that include arteries (e.g., the dorsal aorta [DA]) also physically contain HSCs^41^^,^^42^^,^^43^^,^^44^ and HSC precursors.^29^^,^^45^^,^^46^^,^^47^^,^^48^^,^^49^^,^^50^^,^^51^^,^^52^ However, it remains controversial whether artery ECs directly give rise to HSCs,^41^^,^^53^ or alternatively, non-arterial cells spatially juxtaposed nearby arteries generate HSCs.^11^^,^^13^^,^^15^^,^^16^^,^^17^^,^^54^^,^^55^^,^^56^^,^^57^

These questions surrounding the embryonic origins of blood have complicated the differentiation of hPSCs into blood and immune cells *in vitro*.^11^^,^^12^^,^^58^^,^^59^ There have been spectacular successes in differentiating hPSCs into hematopoietic progenitors that are capable of forming myeloid, erythroid, and lymphoid cells *in vitro*.^1^^,^^2^^,^^3^^,^^4^ However, multiple challenges remain. First, differentiation typically generates heterogeneous populations comprising both blood and non-blood cells. This suggests that, at every step of differentiation, the signals controlling the segregation between blood vs. non-blood cells remain incompletely defined. Second, despite tremendous progress in differentiating hPSCs toward hematopoietic progenitors, the progenitors generated by current protocols often minimally express key HSC transcription factors including *HOXA5*, *HOXA7*, *HOXA9*, and *HOXA10* (collectively referred to as “*HOXA5-10*”) and *HLF*,^60^^,^^61^^,^^62^^,^^63^^,^^64^ with some exceptions.^65^ *Hlf* is required for HSC function^66^ and constitutes an exquisitely specific HSC marker.^67^^,^^68^^,^^69^^,^^70^^,^^71^^,^^72^^,^^73^ Likewise, *HOXA5-10* are required for HSC function,^62^^,^^74^ and forced expression of *HOXA* and other genes enhances the engraftment of hPSC-derived hematopoietic progenitors.^75^^,^^76^ This underscores the importance of generating *HLF*+ *HOXA*+ hematopoietic progenitors from hPSCs and discovering the extracellular signals that ignite expression of these hallmark HSC markers.

Here we revisited the developmental precursors of HSCs *in vivo* to efficiently reconstitute the developmental pathway leading from hPSCs to *HLF*+ *HOXA*+ hematopoietic progenitors *in vitro*. First, our non-invasive genetic lineage tracing in mouse embryos pinpointed artery ECs as the *in vivo* precursors to HSCs. Arteries were the dominant, if not exclusive, source of HSCs. Intriguingly, artery ECs were only fleetingly competent to generate HSCs for ∼2.5 days (E8.5–E11).

Guided by the arterial origins of HSCs *in vivo*, we sequentially differentiated hPSCs into posterior primitive streak (PPS), lateral mesoderm, artery ECs, hemogenic ECs, and >90% pure hematopoietic progenitors *in vitro*. We defined the combinations and timings of extracellular signals that had to be turned on and off at each step of hPSC differentiation to effect efficient differentiation and to block differentiation into unwanted cell-types at each lineage branchpoint. At the very first differentiation step, we generated different types of anterior primitive streak (APS), mid primitive streak (MPS), and PPS. Posterior primitive streak already expressed *HOXA5-HOXA10* and was uniquely competent to generate *HLF*+ *HOXA+* hematopoietic progenitors. APS- and MPS-derived ECs, which lacked *HOXA5-HOXA10* expression, could also generate hematopoietic progenitors, but these lacked *HLF* and *HOXA* expression. This emphasizes the importance of an artery EC’s developmental history (i.e., its PPS provenance) in equipping it with the competence to subsequently generate *HLF*+ *HOXA*+ hematopoietic progenitors. Our ability to convert hPSCs into *HLF*+ *HOXA*+ hematopoietic progenitors with near-stochiometric efficiency (1.01 ± 0.15 hematopoietic progenitors produced per input hPSC) provides a powerful foundation for basic research and regenerative medicine and will avail efforts to generate functional HSCs *in vitro*.

## Results

### Genetic lineage tracing reveals that artery ECs form HSCs *in vivo*

**Figure 1:**
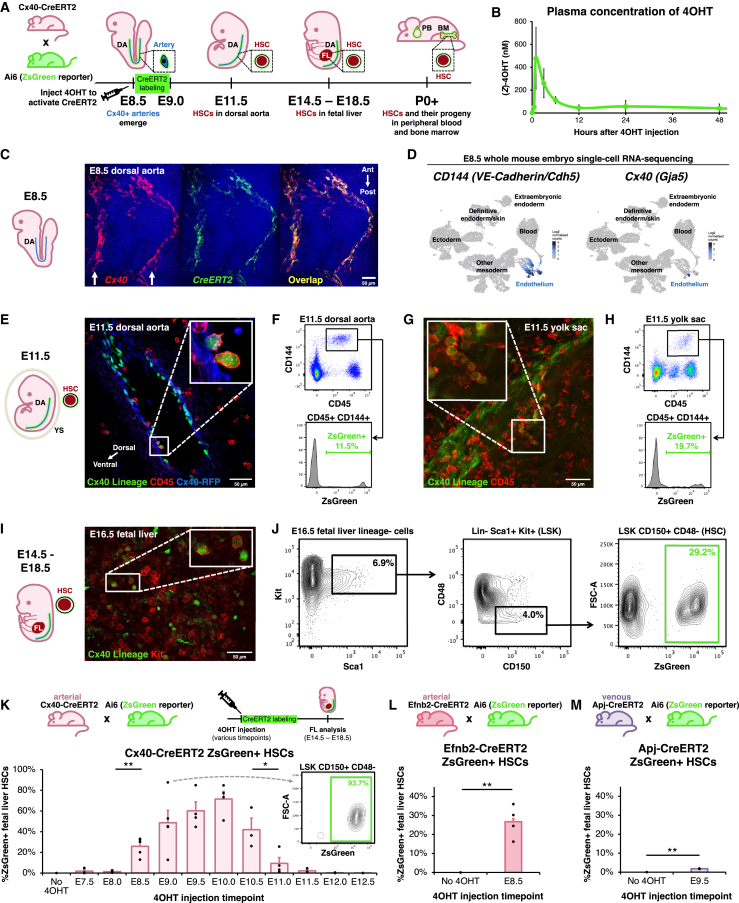
Genetic lineage tracing reveals that artery endothelial cells generate HSCs *in vivo* (A) Experimental strategy. DA, dorsal aorta; FL, fetal liver; PB, peripheral blood; BM, bone marrow; E, embryonic day; P, postnatal day. (B) Mass spectrometry quantification of (*Z*)-4OHT levels in plasma of female adult *Cx40-CreERT2* mice that intraperitoneally injected with (*Z*)-4OHT. (C) *Cx40* and *CreERT2 in situ* staining of E8.5 *Cx40-CreERT2* mouse embryos, using hybridization chain reaction v3.0 (HCR3).^79^ Arrows: paired dorsal aortae.^80^ Ant, anterior; post, posterior. (D) scRNA-seq of the entire E8.5 mouse embryo.^81^ (E–J) Arteries were lineage-traced in *Cx40-CreERT2*; *Ai6* (*ZsGreen* reporter) embryos by administering 4OHT at E8.5. The *Cx40-CreERT2* allele also encodes *RFP*, which was used to visualize Cx40+ cells.^77^ (E, G, and I) Immunostaining and (F, H, and J) flow cytometry of E11.5 dorsal aorta, E11.5 yolk sac, and E16.5 fetal liver was performed. (K) Arteries were lineage-traced in *Cx40-CreERT2*; *Ai6* (*ZsGreen* reporter) embryos by administering a single 4OHT dose at the indicated times (E7.5–E12.5). Flow cytometry was performed to quantify artery-derived (i.e., ZsGreen+) HSCs in the E14.5–E18.5 fetal liver. Each dot: independent litter. For each time point, ≥8 independent embryos were analyzed. Inset: fetal liver HSCs labeled after E9.0 4OHT administration. (L) Arteries were lineage-traced in *Efnb2-CreERT2*; *Ai6* (*ZsGreen* reporter) embryos by administering 4OHT at E8.5. Flow cytometry was performed to quantify ZsGreen+ E14.5–E18.5 fetal liver HSCs. (M) Veins and capillaries were lineage-traced in *Apj-CreERT2*; *Ai6* (*ZsGreen* reporter) embryos by administering 4OHT at E9.5. Flow cytometry was performed to quantify ZsGreen+ E14.5–E18.5 fetal liver HSCs. Histograms depict the mean ± standard error of the mean (SEM). ^∗^p < 0.05, ^∗∗^p < 0.01. Scale bars, 50 μm. Related to Figures S1 and S2 and Table S1.

To stringently test whether arteries form HSCs *in vivo*, we performed non-invasive genetic lineage tracing with an artery-specific, tamoxifen-inducible *Cx40-CreERT2* driver^77^ crossed to a Cre-dependent *ZsGreen* reporter^78^ (Figure 1A). In this approach, Cx40^+^ artery ECs—and all of their progeny cells, even if they downregulate Cx40—are permanently labeled with fluorescent ZsGreen protein. To initiate lineage tracing, we employed (*Z*)-4-hydroxytamoxifen (4OHT), which has a shorter half-life *in vivo* than tamoxifen and permits a restricted labeling period. Within 12 h post-injection, 4OHT declined to almost undetectable levels (half-life <3 h), as shown by mass spectrometry (Figure 1B). Taken together, 4OHT acutely labels cells within a ∼12-h window. This thus circumvents a limitation of tamoxifen, which was previously used to label presumed HSC precursors, but perdures for several days *in vivo*,^34^^,^^82^ incurring the risk of inadvertently labeling emerging HSCs as well.

We selected *Cx40-CreERT2* for arterial lineage tracing because *Cx40* (*Connexin 40/Gja5*) encodes a gap-junction protein specific to artery ECs.^28^^,^^77^^,^^80^^,^^83^^,^^84^ The earliest arterial ECs in the ∼E8.5 DA express *Cx40*,^80^ which colocalizes precisely with *CreERT2* in *Cx40-CreERT2* embryos (Figure 1C). scRNA-seq of the entire mouse embryo revealed that *Cx40* is exquisitely specific to artery ECs from E8.5 to E11^81^^,^^85^: it was expressed by virtually no other cells in the body, except endocardial ECs at E11 (Figures 1D, S1A, and S1B). Importantly, *Cx40* was minimally expressed by E8–E11 hematopoietic stem and progenitor cells (HSPCs), as shown across three scRNA-seq datasets (Figures 1D, S1A, S1C, and S1D).^28^^,^^81^^,^^85^ Indeed, gap junctions are specific to solid tissues and do not conjoin blood cells.^86^ *Cx40-CreERT2* thus affords improved specificity compared to previous *VE-Cadherin-CreERT2* lineage tracing models,^34^ as VE-Cadherin (CD144) is expressed by both HSCs and ECs.^35^^,^^36^^,^^37^

Cx40^+^ artery ECs were lineage-traced by administering 4OHT at E8.5; these gave rise to arterially derived (i.e., ZsGreen+) CD45+ CD144+ HSPCs in the E11.5 DA (Figures 1E, 1F, and S2A–S2C) and yolk sac (Figures 1G and 1H). Of note, the first adult-engrafting HSCs within the DA are CD45+ CD144+.^35^ Subsequently, arterially derived Lineage^−^ Sca1^+^ Kit^+^ (LSK) CD150+ CD48− HSCs^87^ arose in the E14.5–E18.5 fetal liver (FL) (Figures 1I, 1J, S2D, and S2E). In summary, cell surface marker-defined HSCs arise from arteries; below, we demonstrate that these HSCs are in fact functional.

### Artery ECs are competent to generate HSCs for a brief period (E8.5–E11.0) *in vivo*

Do HSCs continuously emerge from arteries, or are they instead produced in a brief burst? To delineate precisely *when* arteries are competent to generate HSCs *in vivo*, we injected a single dose of 4OHT at 12-h increments between E7.5 and E12.5 (Figure 1K). Strikingly, arteries were only competent to form HSCs during a narrow developmental window: 4OHT injection between E8.5 and E10.0 led to steadily increasing percentages of ZsGreen+ FL HSCs, but arteries labeled from E11.5-onward failed to generate appreciable numbers of HSCs (Figure 1K). Labeling peaked at ∼93.7%, suggesting that most—if not all—HSCs originated from a *Cx40*+ arterial precursor (Figure 1K). As expected, 4OHT administration at E7.5 and E8.0 did not lead to labeled HSCs (Figure 1K), as *Cx40*+ artery ECs only arise at E8.5^80^; this underscores the extremely narrow labeling window of 4OHT, which does not meaningfully perdure beyond 12 h (Figure 1B). Additionally, *Cx40-CreERT2* labeling at E11.5–E12.5 did not directly label HSCs (Figure 1K), which are abundant within the embryo at these stages.^41^^,^^43^^,^^44^^,^^88^ This reiterates that *Cx40* is an artery-specific marker suitable to mark HSC precursors, as it is not expressed by HSCs (cf. VE-Cadherin^34^). Taken in collective, arteries are briefly competent to form HSCs for ∼2.5 days (E8.5–E11.0), thus delimiting a narrow time window for *de novo* HSC generation.

To independently confirm the arterial origin of HSCs using a second, well-known arterial marker (*Efnb2*; Figure S1B),^80^^,^^89^^,^^90^ we engineered a *Efnb2-CreERT2* knock-in mouse (Figure S2F). 4OHT labeling of E8.5 artery ECs using *Efnb2-CreERT2* likewise revealed that they subsequently transformed into FL HSCs (Figure 1L). This provides further evidence for the arterial origin of HSCs.

Additionally, we found that embryonic vein and capillary ECs minimally form HSCs. We genetically labeled vein and capillary ECs using *Apj-CreERT2* (Figure 1M).^90^^,^^91^ At E8.5, *Apj* (*Aplnr*) marks mesoderm, but is restricted to vein and capillary ECs by E9.5 and thereafter.^84^^,^^90^^,^^92^^,^^93^^,^^94^ Vein and capillary ECs labeled with *Apj-CreERT2* at E9.5 generated few, if any, FL HSCs (2%) (Figures 1M and S2G). Taken together, arteries—but not veins or capillaries—are the dominant source of HSCs *in vivo*.

Finally, artery ECs also potentially contributed to adult tissue-resident macrophages, including liver Kupffer cells and, to a lesser degree, brain microglia (Figures S2H–S2K). Tissue-resident macrophages arise from primitive myeloid progenitors, but not HSCs.^95^^,^^96^^,^^97^^,^^98^ Nevertheless, our results imply that both tissue-resident macrophages and HSCs ultimately arise from artery ECs. Raw data for all lineage tracing experiments are tabulated in Table S1.

### Artery-derived HSCs are functional *in vivo*

**Figure 2:**
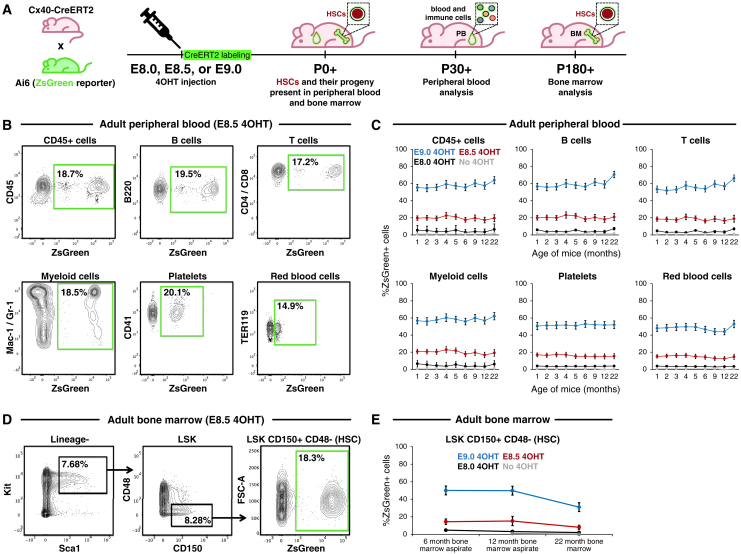
Artery-derived HSCs are functional *in vivo* (A–E) Arteries were lineage-traced in *Cx40-CreERT2*; *Ai6* (*ZsGreen* reporter) embryos by administering 4OHT at either E8.0, E8.5, or E9.0. After embryos developed into adults, flow cytometry was performed to quantify ZsGreen+ cells in (B) and (C) peripheral blood and (C) and (D) bone marrow HSCs in 1- to 22-month-old adult mice. Line graphs depict the mean ± SEM. Related to Figure S3.

Cx40^+^ artery-derived HSCs were functional: they self-renewed to generate additional HSCs and produced all major blood and immune cell-types *in vivo* over prolonged periods.^5^^,^^99^^,^^100^^,^^101^ First, long-term lineage tracing of E8.5 Cx40^+^ artery ECs (Figure 2A) revealed that they generated all major adult blood and immune lineages (including B cells, T cells, monocytes/granulocytes, red blood cells, and platelets; Figures 2B and 2C) and HSCs (Figures 2D and 2E) for the entire adult lifespan (22 months). This suggests long-term self-renewal and differentiation of artery-derived HSCs. Additionally, artery ECs contributed to all major blood lineages at similar frequencies (Figures 2B and 2C), indicating balanced blood lineage production. Cx40^+^ artery ECs labeled by 4OHT at E9.0 contributed to adult HSCs and mature blood and immune lineages even more extensively, whereas 4OHT administration at E8.0 (which would be expected to minimally label arteries) led to negligible contribution (Figures 2C, 2E, and S3A–S3D).

**Figure 3:**
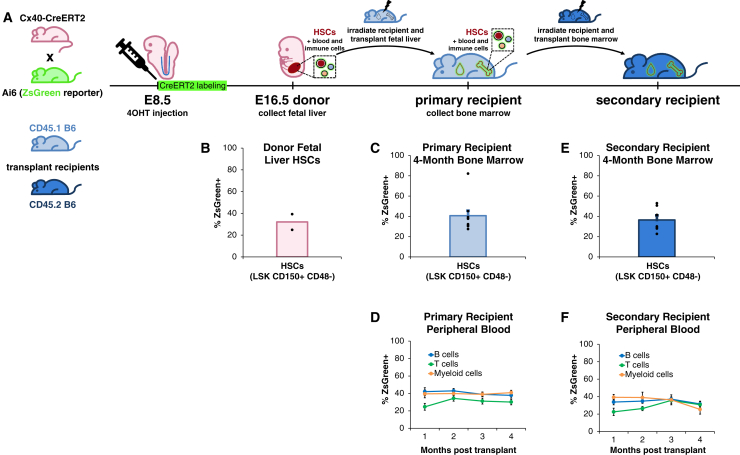
Artery-derived HSCs are functional *in vivo* upon transplantation (A and B) Arteries were lineage-traced by administering 4OHT to E8.5 *Cx40-CreERT2*; *Ai6* (*ZsGreen* reporter) embryos. B6, C57BL/6 mouse. (C and D) ZsGreen+ E16.5 fetal liver HSCs were (B) analyzed by flow cytometry and (C and D) transplanted into lethally irradiated primary recipient mice. 1–4 months post transplantation, flow cytometry was performed to quantify ZsGreen+ (C) peripheral blood cells and (D) bone marrow HSCs in primary recipients. (E and F) Bone marrow from primary recipient mice was transplanted into lethally irradiated secondary recipient mice. 1–4 months post transplantation, flow cytometry was performed to quantify ZsGreen+ (E) peripheral blood and (F) bone marrow HSCs in secondary recipients. Data depict the mean ± SEM. Each dot represents a single mouse. Related to Figure S4.

Additionally, Cx40^+^ artery-derived FL HSCs (Figures 3A, 3B, and S4A) could reconstitute the blood and immune system upon transplantation into lethally irradiated recipient mice, whereupon they regenerated the bone marrow (BM) HSC compartment (Figures 3C and S4B), as well as B cells, T cells, monocytes/granulocytes, red blood cells, and platelets for 4 months (Figures 3D and S4C). Similar results were observed upon serial transplantation into lethally irradiated secondary recipient mice for 4 months (Figures 3E, 3F, S4D, and S4E). Taken together, upon both primary and secondary transplantation, artery-derived HSCs generated all major blood and immune cell-types within the peripheral blood (PB), and reconstituted the HSC pool within the BM, of recipient mice. Our lineage tracing strongly supports the hypothesis that artery ECs generate HSCs *in vivo*, and further reveals that artery ECs are only competent to produce HSCs for a restricted time frame (E8.5–E11.0).

### Differentiation of hPSCs into PPS is critical to ignite *HOXA5-HOXA10* expression at the beginning of differentiation

**Figure 4:**
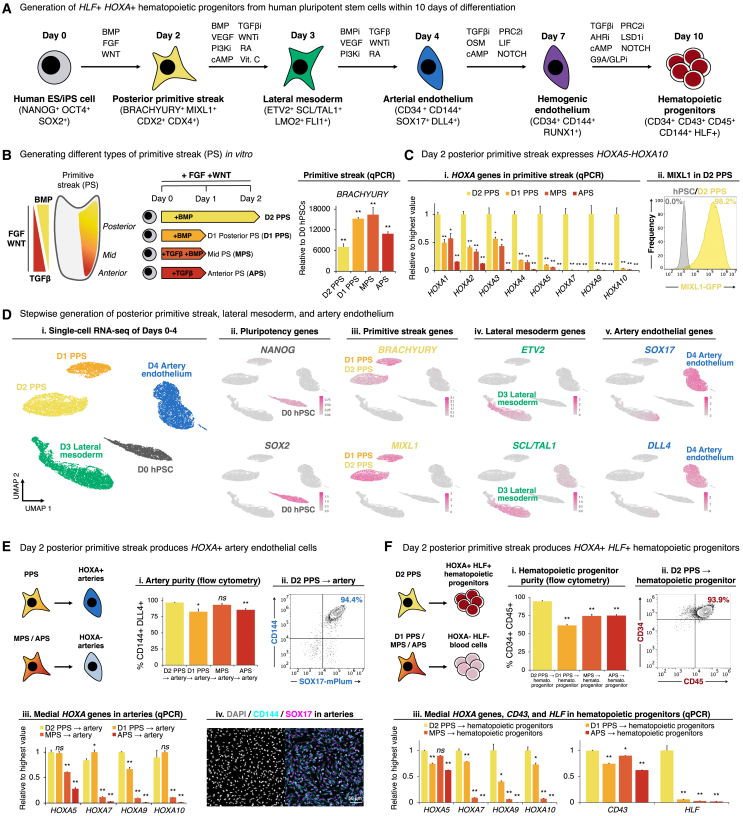
Differentiation of hPSCs into posterior primitive streak competent to subsequently generate *HOXA*+ artery ECs and *HLF*+ *HOXA*+ hematopoietic progenitors (A) Summary (this study). (B and C) qPCR of hPSCs differentiated into anterior primitive streak (day 1, “APS”), mid primitive streak (day 1, “MPS”), posterior primitive streak (day 1, “day-1 PPS”), or posterior primitive streak (day 2, “day-2 PPS”). (Cii) Flow cytometry of day-2 PPS generated from *MIXL1-GFP* hPSCs.^102^ (D) scRNA-seq of differentiated hPSCs. Colors in the left-most panel indicate differentiation day. (E and F) hPSCs were initially differentiated into APS, MPS, day-1 PPS, or day-2 PPS, and then further differentiated into (E) artery ECs or (F) hematopoietic progenitors, which were profiled by (Ei, Eii, Fi, and Fii) flow cytometry, (Eiii and Eiii) qPCR, and (Eiv and Fiv) immunostaining. Scale bars, 50 μm. Histograms depict the mean ± SEM. ^∗^p < 0.05, ^∗∗^p <0.01, n.s., not significant. Related to Figure S5.

Our lineage tracing suggests that artery ECs generate HSCs *in vivo*, and we therefore sought to recapitulate this developmental trajectory *in vitro*. We developed a method to sequentially differentiate hPSCs into PPS, lateral mesoderm, artery ECs, hemogenic ECs, and subsequently hematopoietic progenitors within 10 days, at high efficiency (Figure 4A). While we previously differentiated hPSCs into MPS^103^ and subsequently, lateral mesoderm and artery ECs,^104^ we were unable to differentiate these artery ECs into *HLF*+ *HOXA*+ hematopoietic progenitors (as detailed below). To this end, we first revisited the first step of differentiation: the primitive streak.

*In vivo*, there exist multiple types of primitive streak (anterior, mid, and posterior) that each generate different mesodermal subtypes.^105^^,^^106^^,^^107^*In vivo*, primitive streak is induced by FGF and WNT, which act alongside anteriorizing transforming growth factor β (TGF-β) and posteriorizing BMP gradients^108^^,^^109^^,^^110^^,^^111^^,^^112^^,^^113^^,^^114^ (Figure 4B). Armed with this developmental knowledge, we generated four different types of *BRACHYURY*+ *MIXL1*+ primitive streak: APS, MPS, and PPS (within 1 day of hPSC differentiation), as well as prolonged PPS (within 2 days of hPSC differentiation) (Figures 4B, S5A, and S5B). Only day-2 PPS expressed *HOXA5-HOXA10* (Figures 4Ci and S5Bv), consistent with how *HOX* genes are expressed in PPS^115^^,^^116^^,^^117^ and are expressed *later* in development *in vivo* (spatial and temporal collinearity, respectively).^118^ Congruent with its posterior identity, day-2 PPS also expressed the posterior transcription factors *CDX2* and *CDX4* (Figures S5A and S5C), which are known to induce *HOXA6-HOXA10* and are important for subsequent hematopoietic differentiation.^65^^,^^119^^,^^120^^,^^121^^,^^122^ Production of day-2 PPS was remarkably efficient: over 98% of cells expressed the primitive streak marker MIXL1 (Figure 4Cii), as assessed using *MIXL1-GFP* reporter hPSCs.^102^ scRNA-seq revealed synchronous pluripotency marker downregulation and uniform primitive streak marker expression (Figures 4D, S5B, and S5C).

These four different types of primitive streak generated fundamentally different types of artery ECs and hematopoietic progenitors later during differentiation (Figures 4E, 4F, and S5D–S5F). We previously showed that hPSC-derived day-1 MPS could generate artery ECs,^104^ but here we found these ECs minimally expressed *HOXA5-HOXA10* (Figures 4Eiii and S5E). By contrast, day-2 PPS generated artery ECs that expressed *HOXA5-HOXA10* (Figures 4Eiii, S5B, and S5E), which could further differentiate into CD34+ CD45+ hematopoietic progenitors that expressed the hallmark HSC transcription factors *HLF* and *HOXA5-HOXA10* (Figure 4F). Interestingly, while all four types of primitive streak could form CD34+ CD45+ hematopoietic progenitors, day-2 PPS was solely capable of producing *HLF*+ *HOXA*+ hematopoietic progenitors in our conditions (Figures 4Fiii and S5F–S5H). This reiterates the importance of creating the appropriate type of primitive streak at the very first step of differentiation.

Past studies showed that certain manipulations, such as modulating the RA pathway at intermediate steps of hPSC differentiation, transiently elevated *HOXA* expression, but *HOXA* genes were often turned off upon later differentiation into hematopoietic progenitors.^62^^,^^63^ In development, *HOX* gene expression initiates in the primitive streak and stably persists thereafter,^115^^,^^116^^,^^117^ perhaps explaining why starting differentiation through the appropriate type of primitive streak appears critical to generate *HLF+ HOXA+* hematopoietic progenitors *in vitro*. Below, we detail the extracellular signals that were turned on and off at each step of differentiation to efficiently convert day-2 PPS into *HLF*+ *HOXA*+ hematopoietic progenitors.

### Efficient differentiation of hPSC-derived PPS into artery ECs, hemogenic ECs, and finally, HLF+ HOXA+ hematopoietic progenitors

Next, we differentiated day-2 PPS into day-3 lateral mesoderm, and subsequently day-4 artery ECs. Generation of each of these cell-types required explicit inhibition of signals that would instead generate unwanted cell-types. To differentiate PPS into lateral mesoderm, we activated the BMP (using BMP4), PKA (Forskolin), RA (TTNPB), and VEGF (VEGF) pathways, while simultaneously inhibiting TGF-β (SB505124), WNT (XAV939), and PI3K (GDC-0941) for 24 h. In particular, we explicitly blocked TGF-β and WNT signaling, which instead specify endoderm and lateral mesoderm, respectively.^103^^,^^104^^,^^123^ At this stage, RA activation (TTNPB) promoted *HOXA* expression^62^^,^^63^^,^^124^ (Figure S5I). scRNA-seq revealed that this combination of lateral mesoderm-inducing signals generated enriched *SCL*/*TAL1*+ *KDR*+ lateral mesoderm, with minimal expression of endoderm (*FOXA2*) and paraxial mesoderm (*MSGN1*) markers, thereby reiterating the precision of lateral mesoderm induction (Figures 4D, S5B, and S5C).

Day-3 lateral mesoderm was further differentiated into day-4 artery ECs, by activating TGF-β (using activin A), VEGF (VEGF), and RA (TTNPB), while simultaneously inhibiting BMP (DMH1), WNT (XAV939), and PI3K (GDC-0941), for 24 h. We blocked BMP and PI3K, which respectively induced heart progenitors and vein ECs at this stage of differentiation,^104^ thus consolidating artery specification. At this stage, RA also promoted *HOXA1* expression (Figure S5I). scRNA-seq revealed efficient generation of ∼98.6% pure *SOX17*+ CD144+ artery ECs, with minimal expression of heart (*NKX2.5*) and vein (*APLNR*) markers; the minority (1.4%) of remaining non-ECs corresponded to mesenchymal cells (Figures 4D and S5D). Taken together, we efficiently differentiated hPSCs into PPS, lateral mesoderm, and artery ECs, while inhibiting differentiation into alternate fates at each step of differentiation. Across each of these steps, *HOXA5-HOXA10* genes were continuously expressed (Figure S5Bv).

**Figure 5:**
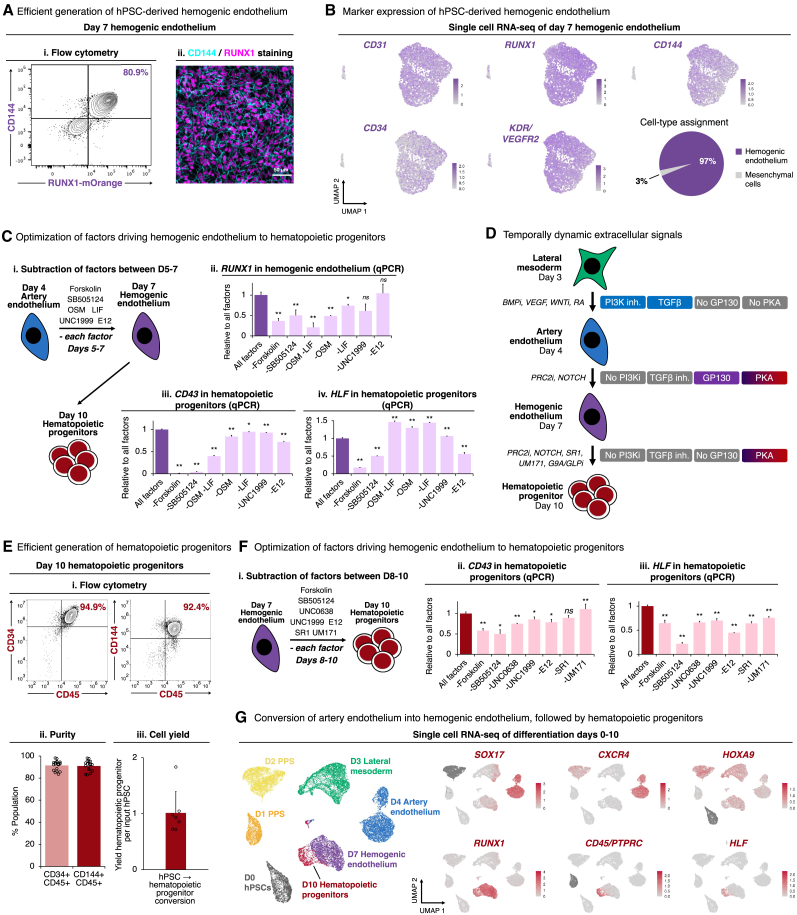
Differentiation of hPSCs into hemogenic ECs and subsequently *HOXA*+ *HLF*+ hematopoietic progenitors (A) Day-7 hemogenic ECs generated from (Ai) *RUNX1-mOrange*^126^ or (Aii) wild-type hPSCs, profiled by flow cytometry or immunostaining, respectively. Scale bars, 50 μm. (B) scRNA-seq of hPSC-derived day-7 hemogenic ECs. Entire population is shown, without preselecting cells. (C) Individual differentiation factors were withheld during differentiation of day-4 hPSC-derived artery ECs into hemogenic ECs over 3 days (Ci), followed by qPCR of day-7 hemogenic ECs (Cii) and day-10 hematopoietic progenitors derived from these hemogenic ECs (Ciii and Civ). (D) Summary (this study). (E) Flow cytometry (Ei and Eii) and absolute number/cell yield (Eiii) of day-10 hPSC-derived hematopoietic progenitors. Each dot: independent experiment. (F) Individual differentiation factors were withheld during differentiation of day-7 hPSC-derived hemogenic ECs into hematopoietic progenitors over 3 days, followed by qPCR of day-10 hematopoietic progenitors. (G) scRNA-seq of differentiated hPSCs. Colors in the left-most panel indicate differentiation day. Histograms depict the mean ± SEM. ^∗^p < 0.05, ^∗∗^p < 0.01; n.s., not significant. Related to Figure S6.

Subsequently, we drove *HOXA*+ artery ECs out of an arterial state and further differentiated them into hemogenic ECs within 3 additional days, by activating the GP130 (using OSM and LIF), NOTCH (DLL4-E12),^125^ and PKA (Forskolin) pathways, while simultaneously inhibiting TGF-β (SB505124) and PRC2 (UNC1999). This yielded >80% pure RUNX1+ hemogenic ECs (Figures 5A, S6A, and S6B), as assessed using *RUNX1-mOrange* reporter hPSCs^126^; *Runx1* expression in ECs *in vivo* signifies their future hematopoietic potential.^127^ scRNA-seq of day-7 differentiated cultures revealed that ∼97% of cells were CD144+ *RUNX1*+ hemogenic ECs, with a small minority (3%) of mesenchymal cells (Figures 5B, S6C, and S6D).

Combinatorial manipulation of these signaling pathways was crucial to efficiently generate hemogenic ECs; individual signals were insufficient. Withholding either GP130 agonists (OSM and LIF), PKA agonist (Forskolin), or TGF-β inhibitor (SB505124) revealed that each of these factors were critical to specify RUNX1+ hemogenic ECs with the future potential to generate blood (Figures 5C and S6E). First, we found that GP130 signaling was crucial for human hemogenic EC specification (Figure 5C), paralleling its role in mouse and zebrafish hematopoietic development.^128^^,^^129^^,^^130^ Second, we discovered that PKA activation specified human hemogenic ECs: in model organisms, both prostaglandin E2 and shear stress activate PKA to induce *Runx1* expression.^131^^,^^132^^,^^133^^,^^134^ Our use of PKA agonist may thus partly alleviate the requirement for shear stress in blood development.^131^^,^^135^^,^^136^ Third, high cell density was crucial to specify hemogenic ECs (Figure S6F). In summary, we discovered temporally dynamic signals convert artery ECs into hemogenic ECs: while VEGF and TGF-β initially induced arterial fate, subsequently withholding these signals and providing others (e.g., GP130 and PKA) drove cells out an arterial fate and specified hemogenic ECs (Figure 5D).

Finally, we differentiated RUNX1+ hemogenic ECs into HLF+ HOXA+ hematopoietic progenitors within 3 additional days (Figures 5E and S6G). To specify hematopoietic progenitors, we activated PKA (using Forskolin), while inhibiting TGF-β (SB505124), inhibiting PRC2 (UNC1999), inhibiting G9A/GLP (UNC0638), inhibiting aryl hydrocarbon receptor (SR1), and inhibiting LSD1 (UM171). Withholding any of these individual factors reduced *HLF*, attesting to their combined significance (Figures 5F and S6H). Specifically, we inhibited PRC2 (Figure S6I), because repression of PRC2/EZH1 precociously induces HSCs *in vivo*.^137^ We also provided UM171,^138^^,^^139^ SR1,^140^ and G9A/GLP inhibitors^141^^,^^142^ to stabilize incipiently arising *HLF*+ *HOXA*+ hematopoietic progenitors in an undifferentiated state and to reduce their spontaneous differentiation into downstream progeny.

These combined signals yielded 91.5% ± 1.0% pure CD34+ CD45+ and 91.0% ± 1.0% pure CD144+ CD45+ hematopoietic progenitors by day 10 of hPSC differentiation, as observed across 20 independent experiments in two different hESC lines: H1 and H7 (Figure 5E). Of note, the first adult-engrafting HSCs within the human and mouse embryo are CD144+ CD45+,^35^^,^^156^ and CD45 distinguishes blood cells from ECs.^37^^,^^157^ Each 1 input hPSC yielded 1.01 ± 0.15 output hematopoietic progenitors (n = 8 independent experiments), indicating near-stochiometric conversion of hPSCs into hematopoietic progenitors (Figure 5Eiii). Similar results were observed with the hiPSC line WTC11 (Figure S6J).

Conversion of hemogenic ECs into hematopoietic progenitors was visually accompanied by progressive emergence of round, semi-adherent cells from the EC monolayer (Figure S6K), and by expression of GFI1, a transcriptional repressor of endothelial genes^158^^,^^159^ (Figure S6L). Transcriptionally, arterial markers *SOX17* and *CXCR4* became downregulated in hemogenic ECs, followed by the gain of *HLF* and *CD45* in emerging hematopoietic progenitors; *HOXA9* was expressed throughout this entire differentiation process (Figure 5G). An interactive scRNA-seq browser is available online, and constitutes a rich resource to conveniently identify markers and candidate regulators of each differentiation stage (https://anglohlabs.shinyapps.io/blood_devcell_shiny/).

### hPSC-derived hematopoietic progenitors express *HLF*, *HOXA5-10*, and other hallmark HSC transcription factors

**Figure 6:**
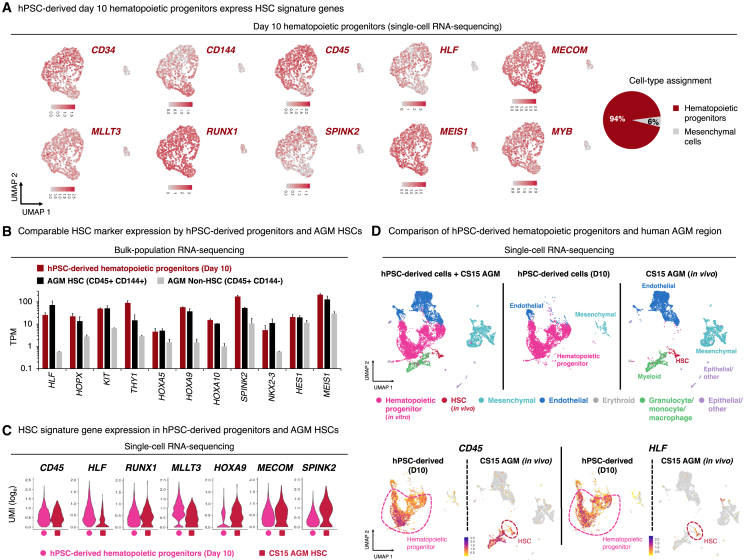
hPSC-derived *HLF*+ *HOXA*+ hematopoietic progenitors express similar levels of hallmark HSC markers as human fetal dorsal aorta HSCs (A) scRNA-seq of day-10 hPSC-derived hematopoietic progenitors, showing gene expression (left) and clustering-based cell-type assignment (right). Entire population is shown, without preselecting cells. (B) Bulk-population RNA-seq comparison of day-10 hPSC-derived hematopoietic progenitors vs. CD45+ CD144+ HSCs vs. CD45+ CD144− non-HSCs from Carnegie stage 15/16 human embryo aorta-gonad-mesenephros (AGM) region.^143^ TPM, transcripts per million. (C and D) scRNA-seq of day-10 hPSC-derived hematopoietic progenitors, compared with (C) HSCs and (D) all cells originating from CS15 human embryo AGM.^30^ Histograms depict the mean ± SEM. Related to Figure S7.

scRNA-seq revealed that ∼94% of day-10 cells were hematopoietic progenitors that expressed HSC transcription factors (*HLF*, *MECOM*, *RUNX1*, *MEIS1*, *MYB*), HSC chromatin regulators (*MLLT3*), and HSPC surface markers (*CD34*, *CD45/PTPRC, CD144*); there was also a small proportion (6%) of remaining mesenchymal cells (Figures 6A, S6M, and S7A). At this stage, we did not detect *IL7R*+ lymphoid or *GATA1*+ erythroid progenitors (Figure S6M), which exist alongside *HLF*+ HSPCs in the human DA.^30^^,^^31^ This may be attributable to our aforementioned use of inhibitors to suppress the precocious differentiation of hPSC-derived hematopoietic progenitors into downstream progeny.

We compared hPSC-derived day-10 *HLF*+ *HOXA*+ hematopoietic progenitors to published scRNA-seq profiles of hPSC-derived hematopoietic progenitors produced from three differentiation protocols.^30^^,^^144^^,^^145^ These three protocols generated CD34+ CD43+ hematopoietic progenitors to varying extents, but *HLF* expression was not appreciably detected (Figure S7B).

Bulk RNA-seq revealed that hPSC-derived day-10 hematopoietic progenitors and human embryonic DA CD144+ CD45+ HSCs^143^ expressed comparable levels of HSC signature transcription factors, including *HLF*,^66^^,^^67^^,^^68^^,^^69^^,^^70^^,^^71^^,^^72^^,^^73^ *HOPX*,^160^ *NKX2.3*,^161^ *MEIS1*,^162^^,^^163^^,^^164^^,^^165^ and *HOXA* genes (*HOXA5*, *HOXA9*, and *HOXA10*)^62^^,^^65^^,^^74^ (Figures 6B and S7C). As a negative control, human DA CD144− CD45+ non-HSCs minimally expressed these HSC signature transcription factors (Figures 6B and S7C).

Likewise, scRNA-seq revealed that six previously defined HSC signature genes (*HLF*, *RUNX1*, *MLLT3*, *HOXA9*, *MECOM*, and *SPINK2*) were comparably expressed between hPSC-derived hematopoietic progenitors and Carnegie stage 15 (CS15) human embryonic DA HSCs^30^ (Figure 6C). In addition to HSCs, the DA also contained additional cell-types (mesenchymal, endothelial, myeloid, and epithelial cells) that were scarce in, or absent, from our hPSC-derived population (Figures 6D and S7D); this reiterates the precision of our *in vitro* differentiation protocol in suppressing unwanted cell-type emergence. Taken together, we generated hPSC-derived hematopoietic progenitors expressing *HLF*, *HOXA5-10*, and other signature HSC markers that were previously challenging to upregulate in other hPSC differentiation protocols.

Nevertheless, there were transcriptional differences between hPSC-derived *HLF*+ *HOXA*+ hematopoietic progenitors and primary HSCs. In particular, hPSC-derived hematopoietic progenitors minimally expressed class II HLA genes, which were instead expressed by primary human HSCs (Tables S2 and S3). hPSC-derived *HLF*+ *HOXA*+ hematopoietic progenitors may therefore approximate early *HLF*+ HSPCs in hematopoietic development, which progressively mature upon progression into the FL and eventually activate class II HLA and other genes.^30^^,^^31^

### hPSC-derived *HLF*+ *HOXA*+ hematopoietic progenitor cells can generate T, B, NK, myeloid, and erythroid cells *in vitro*

**Figure 7:**
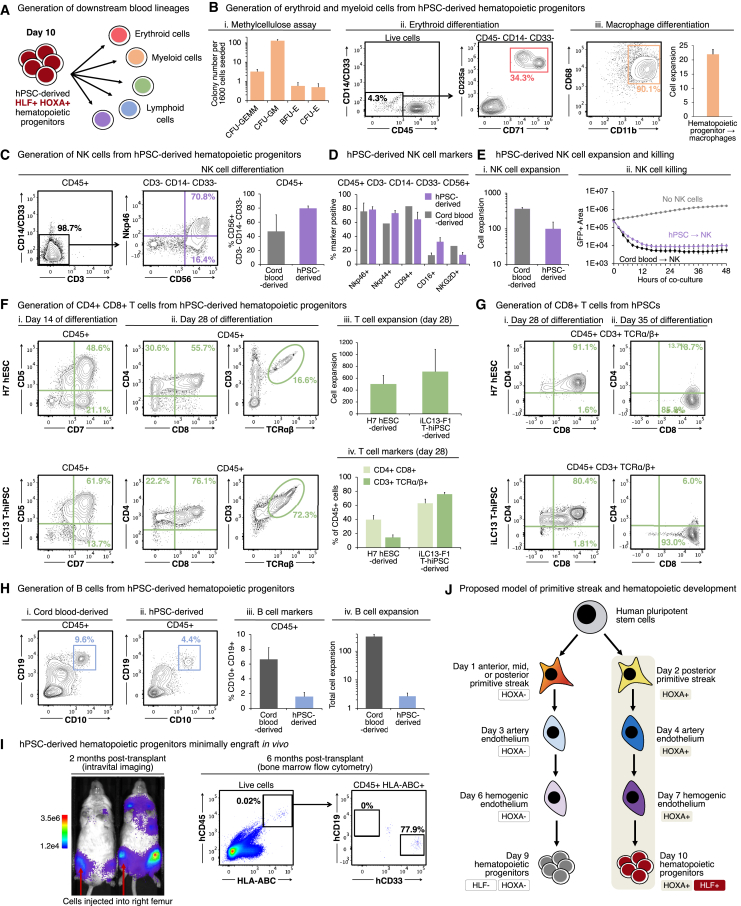
hPSC-derived *HLF*+ *HOXA*+ hematopoietic progenitors generate lymphoid, myeloid, and erythroid cells *in vitro* (A) Summary (this study). (B) Day-10 hPSC-derived hematopoietic progenitors were differentiated into myeloerythroid cells (Bi), erythroid cells (Bii), or macrophages (Biii). Macrophage number/yield per input progenitor is also shown. (C–E) NK cells differentiated from day-10 hPSC-derived hematopoietic progenitors or cord blood CD34+ HSPCs. (C and D) Flow cytometry analysis, with subgating on indicated populations. (Ei) NK cell number/yield generated per input progenitor. (Eii) Live imaging of NK cells killing fluorescent OP9-DLL4*-IRES-GFP* cells. (F and G) T cells differentiated from wild-type H7 hESCs, iLC13-F1 T-hiPSCs, or cord blood CD34+ HSPCs. Flow cytometry analysis with pregating on indicated populations (e.g., CD45+), and T cell number/yield generated per input progenitor. (H) B cells differentiated from day 10 hPSC-derived hematopoietic progenitors or cord blood CD34+ HSPCs. (Hi–Hiii) Flow cytometry analysis and (Hiv) B cell number/yield generated per input progenitor. (I) H7 *AkaLuciferase*-expressing hPSC-derived day-10 hematopoietic progenitors were intrafemorally transplanted into NSG mice, followed by (Ii) bioluminescent imaging and (Iii) flow cytometry. (J) Developmental model (this study). Histograms depict the mean ± SEM. Related to Figure S7 and Video S1.

Finally, day 10 hPSC-derived *HLF*+ *HOXA*+ hematopoietic progenitors harbored the ability to generate all major types of blood and immune cell *in vitro*: myeloid, erythroid, and lymphoid cells (Figure 7A). First, hPSC-derived hematopoietic progenitors could differentiate into granulocytes, monocytes, megakaryocytes, fetal hemoglobin-expressing erythroid cells, and macrophages (Figures 7B and S7E).

Second, hPSC-derived *HLF*+ *HOXA*+ hematopoietic progenitors could differentiate into natural killer (NK) cells, which expressed archetypic NK cell markers including CD56, NKp46, NKp44, CD94, CD16, and NKG2D (Figures 7C and 7D). There was a 99.4-fold increase in cell numbers during NK cell differentiation (Figure 7Ei), demonstrating the ability to generate large numbers of hPSC-derived NK cells. Live imaging revealed that hPSC-derived NK cells were functional and destroyed target cells within 12 h (Figure 7Eii; Video S1).

Video S1. Live imaging of hPSC-derived NK cells killing target cells, related to Figure 7 hPSC-derived NK cells were cocultured with fluorescently-labeled OP9-DLL4*-IRES-GFP* feeder cells and imaging was performed every hour for 72 hours.

Third, hPSC-derived *HLF*+ *HOXA*+ hematopoietic progenitors could differentiate into T cells in feeder-free conditions. CD5+ CD7+ lymphoid progenitors emerged within 2 weeks, followed by CD4+ CD8+ T cells that coexpressed the T cell receptor (TCRα/β) and coreceptor (CD3) at 4 weeks, and finally, CD8+ TCRα/β+ CD3+ T cells by 5 weeks (Figures 7F and 7G). Similarly, we generated CD8+ TCRα/β+ CD3+ T cells from a T-hiPSC line (iLC13-F1), originally reprogrammed from a patient-derived T cell that recognized an Epstein-Barr virus (EBV) antigen^147^ (Figures 7F and 7G). This thus suggests the feasibility of producing antigen-specific T cells. During T cell differentiation, there was a massive (502.5- to 711.6-fold) increase in cell numbers, attesting to the potential scalability of hPSC-derived T cell manufacturing (Figure 7Fiii). Fourth, hPSC-derived hematopoietic progenitors could differentiate into CD10+ CD19+ B cells (Figure 7H).

Given that hPSC-derived *HLF*+ *HOXA*+ hematopoietic progenitors can be generated with high efficiency and speed, they offer a standardized platform to generate multiple human blood and immune cell-types, building on the past progress in generating these cell-types from hPSCs.^1^^,^^2^^,^^3^^,^^4^

Next, we applied hPSC-derived *HLF*+ *HOXA*+ hematopoietic progenitors to model a hematologic cancer, acute myeloid leukemia (AML).^166^^,^^167^ AML patient-derived hiPSCs bearing a *KMT2A-MLLT3* fusion^146^^,^^166^ were differentiated into *HLF*+ *HOXA*+ hematopoietic progenitors; these could robustly engraft NOD-SCID *Il2rg*^*−/−*^ (NSG) mice and yielded almost exclusively myeloid cells *in vivo* (Figure S7F).

However, genetically normal hPSC-derived *HLF*+ *HOXA*+ hematopoietic progenitors minimally engrafted NSG mice. Bioluminescent imaging^168^ revealed that *AkaLuciferase*-expressing hPSC-derived hematopoietic progenitors transplanted directly into the femur subsequently spread to multiple bones throughout the mouse, as expected for HSPCs^169^^,^^170^ (Figure 7I). However, 6 months post-transplantation, there were very low levels of human blood cells (<0.1% CD45+), the majority of which were CD33+ myeloid cells (Figures 7I and S7G).

## Discussion

Through non-invasive genetic lineage tracing in mice, we definitively confirm that artery ECs generate HSCs *in vivo*, and we define a short time frame of ∼2.5 days (E8.5–E11.0) when arteries are competent to do so. Equipped with this developmental knowledge, we rapidly and efficiently differentiated hPSCs into >90% pure *HLF*+ *HOXA*+ hematopoietic progenitors, which expressed HSC signature transcription factors including *HLF*, *HOXA5*, *HOXA7*, *HOXA9*, and *HOXA10* at levels comparable to human HSCs. This differentiation occurs through five sequential steps (PPS, lateral mesoderm, arterial ECs, hemogenic ECs, and finally, hematopoietic progenitors) in 10 days of serum-free and monolayer culture, without any genetic manipulations. At each step of differentiation, we illuminated the extracellular signals that had to be turned on or off to efficiently segue from one step to the next. Consequently, we achieved near-stochiometric conversion of hPSCs into hematopoietic progenitors (1.01 ± 0.15 hematopoietic progenitors produced per input hPSC). The resultant hPSC-derived *HLF*+ *HOXA*+ hematopoietic progenitors could generate a wide range of blood and immune cells, including T cells, B cells, NK cells, myeloid cells, and erythroid cells. This provides a foundation to reliably and efficiently derive human blood and immune cell-types for regenerative medicine, cancer immunotherapy, disease modeling, and various applications.

### Arteries generate HSCs *in vivo* and a narrow time frame for HSC production

Our genetic lineage tracing provides firm evidence that artery ECs form HSCs *in vivo*. We find that arteries generate up to ∼93.7% of HSCs, implying they are the dominant—if not exclusive—source of HSCs. Whether ECs give rise to HSCs has been a longstanding question, with multiple studies arguing against this hypothesis.^11^^,^^15^^,^^16^^,^^17^^,^^54^ That notwithstanding, it has long been recognized that embryonic tissue fragments containing arteries (e.g., the DA) physically contain HSCs, as revealed by transplantation,^41^^,^^42^^,^^43^^,^^44^ and HSC precursors, as shown by explant culture studies.^29^^,^^45^^,^^46^^,^^47^^,^^48^ However, these pioneering studies could not discriminate which exact cell-type within these tissue fragments generate HSCs *in vivo*. Others have instead suggested that HSC precursors are not artery ECs, but rather represent another lineage physically located in the vicinity of arteries,^11^^,^^54^^,^^55^^,^^56^ or are not ECs at all.^13^^,^^15^^,^^16^^,^^17^^,^^57^

Using two lineage tracing systems (*Cx40-CreERT2* and *Efnb2-CreERT2*), we definitively show that artery ECs give rise to HSCs. A chief advantage of *Cx40* is its exquisite arterial specificity; it is not expressed by HSCs.^77^^,^^80^^,^^83^^,^^84^ These two approaches afford greater genetic specificity compared to a previously used *VE-Cadherin-CreERT2* system,^34^ which labels both ECs and HSCs themselves.^37^

Non-invasive lineage tracing *in vivo* also affords multiple advantages. We directly demonstrate that artery-derived HSCs are functional, thus expanding beyond descriptive live-imaging, imaging, and scRNA-seq studies showing the emergence of cells expressing HSC markers, but which could not functionally interrogate the emergent cells.^21^^,^^22^^,^^23^^,^^24^^,^^25^^,^^26^^,^^27^^,^^28^^,^^29^^,^^30^^,^^31^^,^^32^^,^^33^ Moreover, we show that artery ECs generate HSCs *in vivo*, thus expanding beyond *ex vivo* explant culture systems previously used to investigate HSC origins.^29^^,^^45^^,^^46^^,^^47^^,^^48^^,^^49^^,^^50^^,^^51^^,^^52^

However, our studies raise multiple unresolved questions regarding the arterial origins of HSCs. First, we find that arteries are competent to generate HSCs only for a narrow time frame of ∼2.5 days (E8.5–E11.0). Incredibly, there is thus a brief ∼2.5-day developmental window wherein embryonic arteries give rise to almost all future adult blood cells, which then self-perpetuate for years as arteries cease new blood production. But why are early arteries only briefly competent to generate HSCs? To borrow Waddington’s parlance,^171^ this could reflect a restriction in cell-intrinsic *developmental competence* (i.e., later arteries are intrinsically refractory to produce HSCs) and/or absence of cell-extrinsic *inductive cues* (i.e., HSC-specifying signals are no longer present later in development). HSC production from arteries may be under epigenetic control, as HSCs precociously emerge *in vivo* in mouse embryos lacking the H3K27 methyltransferase *Ezh1*, a PRC2 component.^137^ Such epigenetic regulation could explain why ECs later in development silence *Runx1* and resist conversion into hemogenic ECs.^172^^,^^173^ Consequently, epigenetic alterations may shift the time frame of HSC production from arteries, with interesting implications to potentially re-enable HSC production from adult arteries in the future. Second, we show that artery ECs generate HSCs, but we do not assess which tissue this occurs in; candidates include the DA, yolk sac, placenta, umbilical or vitelline vessels, and heart vasculature.^8^^,^^9^^,^^10^^,^^11^^,^^12^^,^^13^^,^^14^^,^^41^^,^^42^^,^^43^^,^^44^^,^^174^^,^^175^

Finally, why are arteries—but not veins or capillaries—capable of generating HSCs *in vivo*? Our *Apj-CreERT2* lineage tracing reveals that essentially no HSCs emerge from veins or capillaries. The molecular mechanisms underlying why arteries, but not veins or capillaries, can generate HSCs warrant further investigation. The proposed arterial origin of HSCs is consistent with how (1) deletion of artery-specifying genes *Sox17* and *Notch1* leads to a complete loss of engraftable HSCs *in vivo*,^176^^,^^177^^,^^178^^,^^179^^,^^180^ whereas (2) genetic loss of the vein-specifying transcription factor *Nr2f2* converts veins into arteries, and blood cells seemingly emerge from the supernumerary arteries *in vivo*.^181^

### Importance of the earliest primitive streak stage in blood differentiation

Despite considerable past successes in differentiating hPSCs into hematopoietic progenitors, it has long been recognized that certain HSC transcription factors—including *HLF* and *HOXA* family members^60^^,^^61^^,^^62^^,^^63^^,^^64^—have proven difficult to upregulate, piquing the question of how and when to turn them on. Numerous manipulations, including RA activation, have been tested at various stages of hPSC differentiation, but often only transiently upregulate *HOXA*.^62^^,^^63^ Other pioneering studies have instead activated WNT and blocked TGF-β at intermediate stages of hPSC differentiation (days 2–4) toward blood lineages.^65^^,^^182^

We instead revisited the very first step of differentiation: primitive streak induction. During embryonic development, *HOX* genes are first turned on in the primitive streak at the beginning of gastrulation, with posterior *HOX* genes activated in the PPS,^115^^,^^116^^,^^117^ even prior to the emergence of mesoderm. By generating four different types of primitive streak *in vitro*, we reveal that day-2 PPS expresses *HOXA5-HOXA10* and is uniquely competent to differentiate into hematopoietic progenitors expressing *HLF* and *HOXA5-HOXA10* (Figure 7J). In our *in vitro* differentiation system, once *HOXA* gene expression is activated within the primitive streak, these genes are continuously expressed throughout differentiation. This is consistent with how continued *HOX* gene expression persistently encodes a cell’s positional identity in developmental biology.^118^ Interestingly, other types of primitive streak—including MPS^103^—can generate artery ECs,^104^ but these ECs express more anterior *HOXA* genes and cannot produce *HLF*+ *HOXA5-10*+ hematopoietic progenitors. These *HLF*- and *HOXA*-deficient CD34+ CD45+ hematopoietic progenitors obtained from anterior or middle primitive streak may approximate hPSC-derived hematopoietic progenitors produced by alternative differentiation protocols. Based on *HOX* codes, it appears that our hPSC-derived APS, MPS, and PPS can respectively produce anterior, mid, and posterior ECs. Why are posterior ECs uniquely capable of subsequently upregulating *HLF* in our system? Mechanistically, how is anterior-posterior positional identity encoded within incipiently emerging ECs? How do different anterior-posterior identities in ECs exert far-reaching effects on their ability to subsequently produce HSCs or hematopoietic progenitors? Our work supports a model that individual ECs are diverse, and only some are competent to generate HSCs,^47^^,^^183^^,^^184^ which we hypothesize may reflect the diverse primitive streak origins of these different ECs.

### Temporally dynamic signals drive consecutive steps of blood development

One of our principal findings is that key extracellular signaling pathways must be turned on and off every 24 h to effect differentiation; even closely related cell-types (e.g., artery and hemogenic ECs) are specified by diametrically opposed signals (Figure 5D). We must therefore discover the rapid temporal dynamics with which these signals act, and manipulate them with equal dynamism, to efficiently effect differentiation *in vitro*. Prolonged activation or inhibition of these signals instead generates heterogeneous cell populations. For instance, on day 4 of differentiation, TGF-β induces artery ECs,^104^ but 24 h later, TGF-β must be sharply repressed for artery ECs to segue into hemogenic ECs. Conversely, GP130 (OSM and LIF) signaling generates *RUNX1*+ hemogenic ECs on days 5–7, but subsequently these ligands must be withdrawn on days 8–10; their continued addition blocks the production of *HLF*+ hematopoietic progenitors (Figure S6Hii). Remarkably, similar signaling dynamics were inferred from scRNA-seq analyses of human embryos: TGF-β was implied to be initially active in artery ECs, but declined later in hemogenic ECs, whereas GP130-JAK/STAT signaling was predicted to be induced later.^30^ Intriguingly, at later stages, certain signals (e.g., OSM and LIF) enhance production of CD34+ CD45+ hematopoietic progenitors, but decrease *HLF* expression (Figure S6Hii). This emphasizes that there are multiple routes to produce hematopoietic progenitors and care must be taken to specifically induce *HLF*+ *HOXA*+ hematopoietic progenitors.

Our work also clarifies the exact lineage relationships between hPSC-derived artery and hemogenic ECs, which have been debated for some time.^11^^,^^58^ In one model, artery and hemogenic ECs arise independently from one another,^54^ whereas in another, hemogenic ECs give rise to artery ECs that subsequently develop into blood.^53^^,^^185^ Our *in vitro* work suggests a third model: that artery ECs give rise to hemogenic ECs, which subsequently generate *HLF+ HOXA*+ hematopoietic progenitors. This is congruent with pseudotemporal inferences from human and mouse embryo scRNA-seq data, which suggested a progression from artery ECs to hemogenic ECs to blood,^28^^,^^29^^,^^30^^,^^31^ and related work in zebrafish.^186^

### hPSC-derived *HLF+ HOXA+* hematopoietic progenitors: A platform to efficiently produce human blood and immune cells

By sequentially generating >90% pure artery ECs, and subsequently, *HLF*+ *HOXA*+ hematopoietic progenitors, we provide a standardized, efficient, and reproducible platform to create a range of human blood and immune cells from hPSCs, including T cells, NK cells, and macrophages. There have been many past successes in generating human lymphoid, myeloid, and erythroid cells from hPSCs,^1^^,^^2^^,^^3^^,^^4^ but these often started from an impure cell population containing a subset of hematopoietic progenitors. Starting from nearly pure *HLF*+ *HOXA*+ hematopoietic progenitors may enhance the speed or efficiency of these protocols to generate downstream blood and immune cells, thus providing a boon for regenerative medicine, cancer immunotherapy, disease modeling, and a range of other applications. We also demonstrate a proof-of-principle for genetically engineering the resultant downstream immune cells, by differentiating T-hiPSCs carrying a rearranged TCR specific for a viral antigen. Given that all major blood and immune cell-types naturally derive from *HLF*+ HSCs *in vivo*,^68^^,^^70^^,^^72^ hPSC-derived *HLF*+ *HOXA*+ hematopoietic progenitors may represent a more physiological starting point to derive downstream blood and immune cell-types *in vitro*.

### Limitations of the study

Artery ECs may generate many, but not all, types of blood and immune cells. During development, there are multiple successive waves of blood development.^8^^,^^9^^,^^10^^,^^11^^,^^12^^,^^13^^,^^14^ Of the various hematopoietic waves, which one(s) derive from arterial precursors? While our lineage tracing reveals that artery ECs form E11.5 “definitive” HSCs, it has been asserted that the earliest “primitive” blood cells in the E7.5 yolk sac blood islands may not originate from ECs.^16^^,^^57^ Primitive yolk sac myeloid progenitors give rise to tissue-resident macrophages, long before HSCs emerge.^95^^,^^96^^,^^97^^,^^98^ Intriguingly, our lineage tracing suggests that artery ECs may also contribute to tissue-resident macrophages, including Kupffer cells and, to a lesser extent, microglia. Do artery ECs contribute to both primitive (e.g., tissue-resident macrophage) and definitive (HSC) blood? Do B1a B cells, γδ T cells, and other HSC-independent “primitive” immune cells likewise arise from artery ECs?^13^ Are there different artery EC subsets dedicated to primitive vs. definitive hematopoiesis? Does transitioning through an arterial intermediate dictate whether differentiating cells adopt a primitive vs. definitive blood identity?

Additionally, while the efficient and rapid differentiation of hPSCs into *HLF*+ *HOXA*+ hematopoietic progenitors constitutes a step forward for the field, these cells nevertheless do not robustly engraft *in vivo*. The basis of this important difference remains unknown, given that hPSC-derived hematopoietic progenitors express archetypic HSC signature genes, although their exact expression levels differ (Figures 6 and S7). Perhaps genes integral to HSC homing, survival, and/or self-renewal also differ. The minimal engraftment of our hPSC-derived hematopoietic progenitors intimates that critical signals for HSC specification remain to be discovered. hPSC-derived *HLF*+ *HOXA*+ hematopoietic progenitors may correspond to nascent HSPCs, as studies of human embryos have shown a continuum of *HLF*+ *HOXA*+ HSPCs that exist *in vivo*, starting from the yolk sac and DA and which progressively mature upon entry into the FL.^30^^,^^31^ Understanding the signals that foster subsequent maturation of *HLF*+ *HOXA*+ hematopoietic progenitors into fully fledged HSCs represents a coming challenge for stem cell and developmental biology. The ability to reliably create nearly pure populations of hPSC-derived *HLF*+ *HOXA*+ hematopoietic progenitors may provide an ideal experimental venue for such studies, facilitating genetic and chemical screens to understand how this subsequent maturation step is regulated.

## STAR★Methods

### Key resources table

**Table:** 

REAGENT or RESOURCE	SOURCE	IDENTIFIER
**Antibodies**		

BV421 Anti-human CD45 antibody, for flow cytometry of differentiated hPSCs	Thermo Fisher	404-0459-42
FITC Anti-human CD14 antibody, for flow cytometry of differentiated hPSCs	Biolegend	301804
FITC Anti-human CD33 antibody, for flow cytometry of differentiated hPSCs	Biolegend	303304
PE Anti-human CD235A/GPA antibody, for flow cytometry of differentiated hPSCs	Thermo Fisher	12-9987-82
APC Anti-human CD71 antibody, for flow cytometry of differentiated hPSCs	Thermo Fisher	17-0719-42
APC Anti-mouse/human CD11b antibody, for flow cytometry of differentiated hPSCs	Biolegend	101212
PE Cy7 Anti-human CD68 antibody, for flow cytometry of differentiated hPSCs	Thermo Fisher	25-0689-42
Alexa Fluor 700 Anti-human CD56 antibody, for flow cytometry of differentiated hPSCs	Biolegend	318316
APC Anti-human CD56 antibody, for flow cytometry of differentiated hPSCs	Biolegend	318310
APC-Cy7 Anti-human NKp46 antibody, for flow cytometry of differentiated hPSCs	Biolegend	331950
APC-Cy7 Anti-human CD336 (NKp44) antibody, for flow cytometry of differentiated hPSCs	Biolegend	325123
PE Anti-human CD314 (NKG2D) antibody, for flow cytometry of differentiated hPSCs	Biolegend	320805
PE Anti-human CD94 antibody, for flow cytometry of differentiated hPSCs	Biolegend	305506
PE-Cy7 Anti-human CD16 antibody, for flow cytometry of differentiated hPSCs	Biolegend	302016
APC-Cy7 Anti-human CD3 antibody, for flow cytometry of differentiated hPSCs	Biolegend	344818
PE Anti-human CD3 antibody, for flow cytometry of differentiated hPSCs	Biolegend	300441
Alexa Fluor 700 Anti-human CD3 antibody, for flow cytometry of differentiated hPSCs	Biolegend	300423
PE Anti-human CD5 antibody, for flow cytometry of differentiated hPSCs	Biolegend	300608
APC Anti-human CD7 antibody, for flow cytometry of differentiated hPSCs	BD Biosciences	653311
APC-Cy7 Anti-human CD4 antibody, for flow cytometry of differentiated hPSCs	Biolegend	300518
PE-Cy7 Anti-human CD8 antibody, for flow cytometry of differentiated hPSCs	Biolegend	344712
APC Anti-human TCRα/β antibody, for flow cytometry of differentiated hPSCs	Biolegend	306718
PE-Cy7 Anti-human CD10 antibody, for flow cytometry of differentiated hPSCs	Biolegend	312214
APC Anti-human CD19 antibody, for flow cytometry of differentiated hPSCs	Biolegend	302212
PE Anti-human CD144 antibody, for flow cytometry of differentiated hPSCs	BD Biosciences	560410
FITC Anti-human CD144 antibody, for flow cytometry of differentiated hPSCs	BD Biosciences	560411
APC Anti-human DLL4 antibody, for flow cytometry of differentiated hPSCs	Biolegend	346508
PE-Cy7 Anti-human CD34 antibody, for flow cytometry of differentiated hPSCs	Biolegend	343516
APC Anti-human CD43 antibody, for flow cytometry of differentiated hPSCs	BD Biosciences	560198
PE-Cy7 Anti-human CD45 antibody, for flow cytometry of differentiated hPSCs	BD Biosciences	557748
Alexa Fluor 488 Anti-human CD45 antibody, for flow cytometry of differentiated hPSCs	Biolegend	304017
eFluor 450 Anti-human CD34 antibody, for flow cytometry of human cord blood HSPCs	Thermo Fisher	48-0349-42
FITC Anti-Human CD90 antibody, for flow cytometry of human cord blood HSPCs	BD Biosciences	555595
PE Anti-human CD45RA antibody, for flow cytometry of human cord blood HSPCs	Biolegend	304108
APC Anti-Human CD38 antibody, for flow cytometry of human cord blood HSPCs	BD Biosciences	340439
PE Cy5 anti-human CD2 antibody, for flow cytometry of human cord blood hematopoietic cells (lineage cocktail)	BD Biosciences	555328
PE Cy5 anti-human CD3 antibody, for flow cytometry of human cord blood hematopoietic cells (lineage cocktail)	BD Biosciences	555341
PE Cy5 anti-human CD4 antibody, for flow cytometry of human cord blood hematopoietic cells (lineage cocktail)	BD Biosciences	555348
PE Cy5 anti-human CD7 antibody, for flow cytometry of human cord blood hematopoietic cells (lineage cocktail)	BD Biosciences	555362
PE Cy5 anti-human CD8 antibody, for flow cytometry of human cord blood hematopoietic cells (lineage cocktail)	BD Biosciences	555368
PE Cy5 anti-human CD11b antibody, for flow cytometry of human cord blood hematopoietic cells (lineage cocktail)	BD Biosciences	555389
PE Cy5 anti-human CD14 antibody, for flow cytometry of human cord blood hematopoietic cells (lineage cocktail)	BD Biosciences	562335
PE Cy5 anti-human CD16 antibody, for flow cytometry of human cord blood hematopoietic cells (lineage cocktail)	BD Biosciences	561725
PE Cy5 anti-human CD19 antibody, for flow cytometry of human cord blood hematopoietic cells (lineage cocktail)	BD Biosciences	555414
PE Cy5 anti-human CD20 antibody, for flow cytometry of human cord blood hematopoietic cells (lineage cocktail)	BD Biosciences	555624
PE Cy5 anti-human CD56 antibody, for flow cytometry of human cord blood hematopoietic cells (lineage cocktail)	BD Biosciences	555517
PE Cy5 anti-human CD235A/GPA antibody, for flow cytometry of human cord blood hematopoietic cells (lineage cocktail)	BD Biosciences	559944
Alexa Fluor 647 anti-mouse CD144 antibody, for flow cytometry of mouse embryonic hematopoietic cells	BD Biosciences	562242
V450 anti-mouse CD45, for flow cytometry of mouse embryonic hematopoietic cells	BD Biosciences	560501
BV421 Anti-mouse CD48 antibody, for flow cytometry of mouse HSCs	Biolegend	103428
PE Anti-mouse Sca-1 antibody, for flow cytometry of mouse HSCs	Biolegend	108108
PE-Cy7 Anti-mouse CD150 antibody, for flow cytometry of mouse HSCs	Biolegend	115914
APC Anti-mouse c-Kit (CD117) antibody, for flow cytometry of mouse HSCs	Biolegend	313205
PE Anti-mouse GR-1/Ly-6G antibody, for flow cytometry of mouse hematopoietic cells	Biolegend	108408
PE Anti-mouse Mac-1/CD11b antibody, for flow cytometry of mouse hematopoietic cells	Biolegend	101208
PE-Cy7 Anti-mouse CD45 antibody, for flow cytometry of mouse hematopoietic cells	Biolegend	103114
APC Anti-mouse CD4 antibody, for flow cytometry of mouse hematopoietic cells	Biolegend	100516
APC Anti-mouse CD8a antibody, for flow cytometry of mouse hematopoietic cells	Biolegend	100712
APC-Cy7 Anti-mouse B220/CD45R antibody, for flow cytometry of mouse hematopoietic cells	Biolegend	103224
Brilliant Violet 421 anti-mouse CD41 antibody, for flow cytometry of mouse hematopoietic cells	Biolegend	133912
PE-Cy5 anti-mouse TER119 antibody, for flow cytometry of mouse hematopoietic cells	Thermo Fisher	15-5921-83
APC-eFluor 780 Streptavidin, for flow cytometry of mouse hematopoietic cells (lineage cocktail)	Thermo Fisher	47-4317-82
Biotin Anti-mouse Ter119 antibody, for flow cytometry of mouse hematopoietic cells (lineage cocktail)	Biolegend	116204
Biotin Anti-mouse CD4 antibody, for flow cytometry of mouse hematopoietic cells (lineage cocktail)	Biolegend	100508
Biotin Anti-mouse CD8a antibody, for flow cytometry of mouse hematopoietic cells (lineage cocktail)	Biolegend	100704
Biotin Anti-mouse CD127 (IL7R) antibody, for flow cytometry of mouse hematopoietic cells (lineage cocktail)	Biolegend	135006
Biotin Anti-mouse B220/CD45R antibody, for flow cytometry of mouse hematopoietic cells (lineage cocktail)	Biolegend	103204
Biotin Anti-mouse GR-1/Ly-6G antibody, for flow cytometry of mouse hematopoietic cells (lineage cocktail)	Biolegend	108404
Brilliant Violet 785 Anti-mouse CD45 antibody, for flow cytometry of mouse tissue myeloid cells	Biolegend	103149
Brilliant Violet 711 Anti-mouse/human CD11b antibody, for flow cytometry of mouse tissue myeloid cells	Biolegend	101242
Brilliant Violet 650 Anti-mouse F4/80 antibody, for flow cytometry of mouse tissue myeloid cells	Biolegend	123149
APC/Cyanine7 Anti-mouse CX3CR1 antibody, for flow cytometry of mouse tissue myeloid cells	Biolegend	149048
Brilliant Violet 421 Anti-mouse CD206 antibody, for flow cytometry of mouse tissue myeloid cells	Biolegend	141717
BD Horizon V450 Mouse anti-Human CD45 antibody, for flow cytometry of humanized mice	BD Biosciences	560367
APC-Cy7 anti-Human HLA-A/B/C antibody, for flow cytometry of humanized mice	Biolegend	311425
PE anti-Human CD33 antibody, for flow cytometry of humanized mice	Biolegend	303404
APC anti-Human CD19 antibody, for flow cytometry of humanized mice	Biolegend	302212
TruStain FcX anti-mouse CD16/32	Biolegend	101320
Anti-Rat and Anti-Hamster Ig κ Compensation Particles	BD Biosciences	552845
Anti-Mouse Ig κ Compensation Particles	BD Biosciences	552843
FcR Blocking Reagent, human	Miltenyi Biotec	130-059-901
Anti-mouse CD45 Microbeads, for magnetic enrichment	Miltenyi Biotec	130-052-301
Anti-APC Microbeads, for magnetic enrichment	Miltenyi Biotec	130-090-855
Goat anti-human SOX17 antibody, for immunostaining of human cells	R&D Systems	AF1924
Mouse anti-human CD144 antibody, for immunostaining of human cells	BD Biosciences	555661
Rabbit anti-human/mouse/rat RUNX1 antibody, for immunostaining of human cells	Abcam	92336
Mouse anti-human/mouse CD45 antibody, for immunostaining of human cells	eBioscience	14-0459082
Rabbit anti-human/mouse GFI1 antibody, for immunostaining of human cells	Cell Signaling Technology	31929
Rabbit anti-mouse RFP antibody, for immunostaining of mouse embryos	Rockland Immunochemicals	600-401-379
Rat anti-mouse KIT (CD117) antibody, for immunostaining of mouse embryos	Thermo Fisher	14-1171-82
Rat anti-mouse CD144 antibody, for immunostaining of mouse embryos	BD Biosciences	550548
Goat anti-mouse CD45 antibody, for immunostaining of mouse embryos	R&D Systems	AF114-SP
Donkey anti-goat Alexa Fluor 647 antibody	Thermo Fisher	A21447
Donkey anti-mouse Alexa Fluor 555 antibody	Thermo Fisher	A32773
Donkey anti-rabbit Alexa Fluor 647 antibody	Thermo Fisher	A31573
Donkey anti-rat Alexa Fluor 647 antibody	Thermo Fisher	A48268
Donkey anti-goat Alexa Fluor 555 antibody	Thermo Fisher	A21447

**Chemicals, peptides and recombinant proteins**		

mTeSR Plus medium	STEMCELL Technologies	100-0276
Essential 8 medium	Thermo Fisher	A1517001
Penicillin/streptomycin	Thermo Fisher	15-140-122
KnockOut Serum Replacement	Thermo Fisher	10828028
Geltrex LDEV-Free, hESC-Qualified, Reduced Growth Factor Basement Membrane Matrix	Thermo Fisher	A1413302
Recombinant Human Vitronectin Protein, Truncated (VTN-N)	Thermo Fisher	A14700
Versene solution	Thermo Fisher	15040066
TrypLE Express Enzyme (1X)	Thermo Fisher	12604013
Accutase (used in this study for *in vitro* cell dissociation)	Thermo Fisher	00-4555-56
Accutase (used in this study for brain tissue dissociation)	Innovative Cell Technologies	AT104
Type II Collagenase	Thermo Fisher	17101015
Liberase	Roche	5401119001
DNase I	Worthington	LS002007
HBSS without Ca^2+^/Mg^2+^	Thermo Fisher	14175103
HBSS with Ca^2+^/Mg^2+^	Thermo Fisher	24020117
Percoll	Cytiva	17-0891-02
Papain	Worthington Biochemical	LS003118
F062-mercaptoethanol (2-mercaptoethanol)	Thermo Fisher	21985023
Cysteine HCl	Sigma	C7477
M199 Media	Thermo Fisher	11150059
DMEM/F12 + GlutaMAX	Thermo Fisher	10565042
IMDM + GlutaMAX	Thermo Fisher	31980-097
F12 + GlutaMAX	Thermo Fisher	31765-092
αMEM + nucleosides	Thermo Fisher	12-571-063
Polyvinyl alcohol (PVA)	Sigma	P8136-250G
1-thioglycerol	Sigma	M6145-100ML
Chemically defined lipid concentrate	Thermo Fisher	11905-031
Recombinant human insulin	Sigma	11376497001
Human transferrin	Sigma	10652202001
DNase/RNase free water	Thermo Fisher	10977023
EDTA	Thermo Fisher	15575020
DMSO	Sigma Aldrich	D2650-100ML
Thiazovivin	Tocris	3845
Recombinant human VEGF	R&D Systems	293-VE-0500
Recombinant human FGF2	R&D Systems	233-FB-01M
Recombinant human BMP4	R&D Systems	314-BP-050
Recombinant human Activin A	R&D Systems	338-AC-500/CF
GDC-0941	Cellagen Technology	C4321-25
Forskolin	Tocris	1099
XAV939	Tocris	3748
Ascorbic acid-2-phosphate (AA2P)	Sigma	49752-10G
DMH1	Tocris	4126
SB505124	Tocris	3263
CHIR99201	Tocris	4423
TTNPB	Tocris	0761
DLL4-E12	Vincent Luca’s laboratory (Moffitt Cancer Center)	Luca et al.^125^
LIF	R&D Systems	7734-LF-025
OSM	R&D Systems	295-OM-010
SR1	Cellagen	C7710-5
UM171	ApexBio	A8950
UNC0638	Tocris	4343
UNC1999	Tocris	4904
EED226	Selleck Chemicals	S8496
GM-CSF	Peprotech	300-03
M-CSF	Peprotech	300-25
IL-3	Peprotech	200-03
IL-6	Peprotech	200-06
FLT3L	Peprotech	300-19
FLT3L	R&D Systems	308-FK
TPO	R&D Systems	288-TP-005/CF
TPO	Peprotech	300-18
SCF	Peprotech	300-07
SCF	R&D Systems	255-SC
IL-7	Peprotech	200-07
IL-15	Peprotech	200-15
G-CSF	Peprotech	300-23
Bovine serum albumin (BSA)	Sigma	A2153
Bovine serum albumin (BSA), Fraction V	Thermo Fisher	15260-037
Tween-20	Sigma	P9416
Insulin-Transferrin-Selenium-Ethanolamine (ITS-X)	Thermo Fisher	51500-056
Sytox Red Dead Cell Stain	Thermo Fisher	S34859
DAPI	Thermo Fisher	D1306
Propidium iodide (PI)	Biolegend	421301
Propidium iodide (PI)	Sigma	P4170
Donkey Serum	Millipore Sigma	D9663-10ML
Triton X-100	Millipore Sigma	T8787-50ML
Triton X-100	Sigma	X100-500ML
PBS, pH 7.4	Thermo Fisher	10010049
(*Z*)-4-Hydroxytamoxifen (4OHT), for lineage tracing	Millipore Sigma	H7904-25MG
(*E/Z*)-4-hydroxytamoxifen-d5, internal standard for mass spectrometry	Cayman Chemical	34232
Corn oil	Sigma	C8267-500ML
ACK (Ammonium-Chloride-Potassium) Lysing Buffer	Thermo Fisher	A1049201
Dextran from *Leuconostoc spp.*, molecular weight 450,000-650,000	Sigma	31392-50G
32% paraformaldehyde solution	Fisher Scientific	50-980-495
Methanol	Fisher Scientific	A412-1
D-Sucrose	Fisher Scientific	BP220-212
AkaLumine HCl (otherwise known as TokeOni)	Tocris	6555
Ethanol, Absolute (200 Proof), Molecular Biology Grade	Fisher Scientific	BP2818100

**Critical commercial assays**		

RNeasy Micro kit	Qiagen	74004
High-Capacity cDNA Reverse Transcription Kit	Applied Biosystems	4368814
SensiFAST SYBR Green Lo-ROX Kit	Thomas Scientific	BIO-94050
Chromium Single Cell 3’ GEM, Library & Gel Bead Kit v3	10x Genomics	1000075
Chromium Single Cell B Chip Kit, 48 reactions	10x Genomics	1000073
MACS LS column	Miltenyi Biotec	130-042-401
Fetal Bovine Serum (FBS), Premium Select	R&D Systems (formerly Atlanta Biologicals)	S11550
EDTA-coated Microtainer Blood Collection Tube	BD Biosciences	365974
O.C.T. Compound	Fisher Scientific	23-730-571
ProLong Gold Antifade Mountant	Thermo Fisher	P36930
MethoCult H4435 Enriched	STEMCELL Technologies	04435
StemSpan Erythroid Expansion Supplement	STEMCELL Technologies	02692
StemSpan Serum-Free Expansion Medium II (SFEM II)	STEMCELL Technologies	09655
StemPro-34 serum-free medium (SFM)	Thermo Fisher	10640-019
StemSpan T Cell Generation Kit	STEMCELL Technologies	09940
ImmunoCult Human CD3/CD28/CD2 T Cell Activator	STEMCELL Technologies	10970
Bioanalyzer High Sensitivity RNA Analysis	Agilent	5067-1513
Bioanalyzer High Sensitivity DNA Analysis	Agilent	5067-4626
KAPA Library Quantification Kit	Kapa Biosystems	KK4854
Illumina Nextera XT DNA Sample Preparation Kit	Illumina	FC-131-1096

**Deposited data**		

Single-cell RNA-sequencing data of E8.5-E11 whole mouse embryos	Jay Shendure’s laboratory (University of Washington)	NCBI Accession: GSE186069 and NCBI Accession: GSE228590 (Qiu et al.^85^)
Single-cell RNA-sequencing data of hematopoietic progenitor and endothelial cells isolated from the E10-E11 mouse embryo dorsal aorta	Catherine Robin’s laboratory (Hubrecht Institute)	NCBI Accession: GSE112642 (Baron et al.^28^)
Single-cell RNA-sequencing data of whole E8.5 mouse embryos, accessible via online browser	John Marioni’s and Berthold Göttgens’s laboratories (University of Cambridge)	https://marionilab.cruk.cam.ac.uk/MouseGastrulation2018/ (Pijuan Sala et al.^81^)
Bulk population RNA-sequencing data of FACS-purified CD144+ CD45+ HSCs and CD144- CD45+ non-HSCs from human fetal Carnegie Stage 15-16 (CS15-16) dorsal aorta	Alexander Medvinsky’s laboratory (University of Edinburgh)	NCBI Accession: GSE151877 (Crosse et al.^143^)
Single-cell RNA-sequencing profiles of human fetal 5- and 5.5-week-old aorta-gonad-mesenephros (AGM) cells	Hanna Mikkola’s laboratory (University of California Los Angeles)	NCBI Accession: GSE162950, samples GSM4968832 and GSM4968833 (Calvanese et al.^30^)
Single-cell RNA-sequencing profiles of hPSC-derived hematopoietic progenitors, differentiated using the Elefanty, Stanley, and Ng laboratories’ protocol^30^	Hanna Mikkola’s laboratory (University of California Los Angeles)	NCBI Accession: GSE162950, sample GSM6205033 (Calvanese et al.^30^)
Single-cell RNA-sequencing profiles of hPSC-derived hematopoietic progenitors, differentiated using the Keller laboratory’s protocol^54^	Ludovic Vallier’s, Daniel Ortmann’s, and Ana Cvejic’s laboratories (University of Cambridge)	European Bioinformatics Institute Accession: E-MTAB-8205, sample EXP2_CTRL2_4823STDY7231845 (Canu et al.^144^)
Single-cell RNA-sequencing profiles of hPSC-derived hematopoietic progenitors, differentiated using the Zandstra laboratory’s protocol^145^	Peter Zandstra’s laboratory (University of British Columbia)	NCBI Accession: GSE207157, sample GSM6280645 (Michaels et al.^145^)
Bulk-population RNA-sequencing timecourse profiling of hPSCs differentiating into *HLF*+ *HOXA*+ hematopoietic progenitors, differentiated using the present protocol	Kyle Loh’s and Lay Teng Ang’s laboratories (Stanford University)	NCBI Accession: PRJNA1074858 (this study)
Single-cell RNA-sequencing timecourse profiling of hPSCs differentiating into *HLF*+ *HOXA*+ hematopoietic progenitors, differentiated using the present protocol	Kyle Loh’s and Lay Teng Ang’s laboratories (Stanford University)	NCBI Accession: PRJNA1073685, also accessible via an interactive web browser: https://anglohlabs.shinyapps.io/blood_devcell_shiny/ (this study)

**Experimental models: cell lines**		

H1 hESCs	WiCell	WiCell, WA01
H7 hESCs	WiCell	WiCell, WA07
H9 hESCs	WiCell	WiCell, WA09
H1 *SOX17-2A-mPlum* hESCs	Kyle Loh’s and Lay Teng Ang’s laboratories (Stanford University)	Ang et al.^104^
HES3 *MIXL1-GFP* hESCs	Andrew Elefanty’s, Edouard Stanley’s, and Elizabeth Ng’s laboratories (Murdoch Children’s Research Institute)	Davis et al.^102^
TkDA3-4 *RUNX1-2A-mOrange* hiPSCs	Hiromitsu Nakauchi’s laboratory (Stanford University)	Ikeda et al.^126^
iSU223n hiPSCs	Hiromitsu Nakauchi’s laboratory (Stanford University)	Nishimura et al.^146^
iLC13-F1 hiPSCs	Hiromitsu Nakauchi’s laboratory (Stanford University)	Ando et al.^147^
H7 *CAG-AkaLuc-PuroR* hESCs	Kyle Loh’s laboratory (Stanford University)	This study
WTC11 hiPSCs	Coriell Institute for Medical Research	Coriell Institute for Medical Research, GM25256, Kreitzer et al.^148^
OP9-*DLL4-IRES-GFP* feeder cells	Juan Carlos Zúñiga-Pflücker’s laboratory (University of Toronto)	Mohtashami et al.^149^
MS5 feeder cells	DSMZ	ACC 441
Human cord blood CD34+ hematopoietic stem and progenitor cells (pooled from mixed donors)	StemExpress	CB3400.5C

**Experimental models: organisms/strains**		

*Mus musculus (mouse): Cx40-CreERT2*	Lucile Miquerol’s laboratory (Aix-Marseille Université)	Beyer et al.^77^
*Mus musculus (mouse): Efnb2-CreERT2*	Kyle Loh’s laboratory (Stanford University)	This study (being deposited at The Jackson Laboratory [JAX], 038831)
*Mus musculus (mouse): Apj-CreERT2*	Kristy Red-Horse’s laboratory (Stanford University)	Chen et al.^91^
*Mus musculus (mouse): Rosa26-CAG-LoxP-Stop-LoxP-ZsGreen (Ai6)*	The Jackson Laboratory	JAX 007906 (Madisen et al.^78^)
*Mus musculus (mouse):* FVB/NJ	The Jackson Laboratory	JAX 001800
*Mus musculus (mouse):* C57BL/6-CD45.2	The Jackson Laboratory	JAX 000664
*Mus musculus (mouse):* C57BL/6-CD45.1 (Pepboy)	The Jackson Laboratory	JAX 002014
*Mus musculus (mouse):* NOD-SCID *Il2rg-/-* (NSG)	The Jackson Laboratory	JAX 005557 (Shultz et al.^150^)

**Oligonucleotides**		

Primers	See Table S4	N/A
HCR3 probe for mouse *Gja5* (*Cx40*), compatible with amplifier B3	Molecular Instruments	Custom probe against *Gja5* (sequence deposited at NCBI accession NM_001271628)
HCR3 probe for *CreERT2*, compatible with amplifier B1	Molecular Instruments	Custom probe against *CreERT2* (sequence deposited at https://www.addgene.org/14797/)
HCR3 B3-Alexa Fluor 647 amplifier probe	Molecular Instruments	B3-Alexa Fluor 647
HCR3 B1-Alexa Fluor 488 amplifier probe	Molecular Instruments	B1-Alexa Fluor 488

**Software and algorithms**		

anndata	Fabian Theis’s laboratory (Helmholtz Munich)	https://github.com/theislab/anndata
AnnotationDbi	Hervé Pagès, Marc Carlson, Seth Falcon, Nianhua Li (Bioconductor Team)	https://github.com/Bioconductor/AnnotationDbi
biomaRt	Damian Smedley, Syed Haider, Benoit Ballester, Richard Holland, Darin London, Gudmundur Thorisson, Arek Kasprzyk (Bioconductor Team)	https://github.com/grimbough/biomaRt
CellRanger	10x Genomics	https://support.10xgenomics.com/single-cell-gene-expression/software/pipelines/latest/what-is-cell-ranger
DESeq2	Michael Love’s laboratory (University of North Carolina at Chapel Hill)	https://github.com/thelovelab/DESeq2 (Love et al.^151^)
dplyr	Hadley Wickham (Tidyverse Team)	https://github.com/tidyverse/dplyr
ggplot2	Hadley Wickham (Tidyverse Team)	https://github.com/tidyverse/ggplot2
hdf5r	Holger Hoefling and Mario Annau (Novartis Institutes for BioMedical Research)	https://github.com/hhoeflin/hdf5r/
FIJI/ImageJ	Albert Cardona’s laboratory (University of Cambridge)	https://imagej.net/software/fiji/ (Schindelin et al.^152^)
Kallisto	Lior Pachter’s laboratory (California Institute of Technology)	https://pachterlab.github.io/kallisto/ (Bray et al.^153^)
matrix	Timothy Davis’s laboratory (Texas A&M University)	https://Matrix.R-forge.R-project.org
org.Hs.eg.db	Martin Mächler (ETH Zürich)	http://bioconductor.org/packages/org.Hs.eg.db/
patchwork	Thomas Lin Pederson (Unaffiliated)	https://github.com/thomasp85/patchwork
RColorBrewer	Erich Neuwirth (University of Vienna)	http://colorbrewer2.org
RCurl	CRAN Team	https://curl.se/libcurl/
reticulate	Yuan Tang (Reticulate Team)	https://github.com/rstudio/reticulate
rhdf5	Bernd Fischer, Mike Smith, Gregoire Pau, Martin Morgan, Daniel van Twisk (European Molecular Biology Laboratory)	https://github.com/grimbough/rhdf5
RStudio v 2022.12.0+353	RStudio Team	https://www.rstudio.com/
scanpy	Fabian Theis’s laboratory (Helmholtz Munich)	https://github.com/theislab/Scanpy
scCustomize	Samuel Marsh (Children's Hospital Boston)	https://github.com/samuel-marsh/scCustomize
Seurat v4.3.0	Rahul Satija’s laboratory (New York Genome Center)	https://github.com/satijalab/seurat (Hao et al.^154^)
SeuratDisk	Rahul Satija’s laboratory (New York Genome Center)	https://github.com/mojaveazure/seurat-disk
tidyverse	Hadley Wickham (Tidyverse Team)	https://github.com/tidyverse
tximport	Michael Love’s laboratory (University of North Carolina at Chapel Hill)	https://bioconductor.org/packages/release/bioc/html/tximport.html (Soneson et al.^155^)
viridis	Simon Garnier, Noam Ross, Robert Rudis, Pedro Camargo, Marco Sciaini, Cédric Sherer (New Jersey Institute of Technology)	https://github.com/sjmgarnier/viridis
Computational scripts used for genomics analyses in this study	Kyle Loh’s laboratory (Stanford University)	https://github.com/lohlaboratory/blood-differentiation (https://doi.org/10.5281/zenodo.10729568)

### Resource availability

#### Lead contact

Requests for further information should be directed to and will be fulfilled by the lead contact, Kyle M. Loh (kyleloh@stanford.edu).

#### Materials availability

All cell lines will be made freely available upon request and the completion of applicable material transfer agreements. *Efnb2-CreERT2* mice are being deposited at The Jackson Laboratory (JAX strain number 038831).

#### Data and code availability

Bulk-population RNA-sequencing datasets generated as part of this study are available at the NCBI Sequence Read Archive (NCBI Accession: PRJNA1074858).

Single-cell RNA-sequencing datasets generated as part of this study are available at the NCBI Sequence Read Archive (NCBI Accession: PRJNA1073685). Single-cell RNA-seq datasets encompassing differentiated hPSCs that were generated as part of this study can be interactively browsed at a custom web portal: https://anglohlabs.shinyapps.io/blood_devcell_shiny/.

Computational scripts used for genomics analyses conducted as part of this study are available at Github: https://github.com/lohlaboratory/blood-differentiation (https://doi.org/10.5281/zenodo.10729568).

Any additional information required to reanalyze the data reported in this study is available from the lead contact upon request.

### Experimental model and study participant details

#### Cell culture

All cells in this study were cultured in standard incubator conditions (20% O_2_, 5% CO_2_, and 37 °C).

#### Human pluripotent stem cell lines

Wild-type H1, H7, and H9 hESCs have been described previously.^187^ Their genotypes are as follows: H1 hESCs (XY genotype, Central European ethnicity), H7 hESCs (XX genotype, Middle East/East European ethnicity), and H9 hESCs (XX genotype, Middle East/East European ethnicity).^188^

HES3 *MIXL1-GFP* hESCs have been described previously.^102^ HES3 hESCs were genetically engineered to partially replace the coding sequence of the endogenous *MIXL1* gene with a *GFP* reporter.^102^ This approach did not preserve the coding sequence of the endogenous *MIXL1* gene. HES3 hESCs are of an XX genotype and Han Chinese ethnicity.^188^

H1 *SOX17-2A-mPlum* hESCs have been described previously.^104^ H1 hESCs were genetically engineered to replace the stop codon of the endogenous *SOX17* gene with an *2A-mPlum* reporter.^104^ This approach theoretically preserved the coding sequence of the *SOX17* gene.

TkDA3-4 RUNX1-2A*-mOrange* hiPSCs have been described previously.^126^ Human dermal fibroblasts were retrovirally transduced with the reprogramming factors to yield TkDA3-4 hiPSCs,^189^ which were subsequently genetically engineered to replace the stop codon of the endogenous *RUNX1* gene with an *2A-mOrange* reporter.^126^ This approach theoretically preserved the coding sequence of the *RUNX1* gene.

iSU223n hiPSCs have been described previously.^146^ They were reprogrammed from SU223, a 20-year-old female patient with relapsed acute myeloid leukemia (AML), which carried a t(9;11)(p22;q23) chromosomal rearrangement (which encodes a *KMT2A-MLLT3* fusion protein), in addition to *FLT3*^*ITD*^, *NRAS*^*G12D*^, *SEMA4A*^*Y5589H*^, and *WT1*^*T390fs*^ mutations.^166^ SU223 AML cells were reprogrammed using Sendai viruses carrying the reprogramming factors to yield iSU223n hiPSCs,^146^ which were subsequently engineered to express *BFP* and a cell-cycle fluorescent marker.

iLC13-F1 hiPSCs have been described previously.^147^ They were reprogrammed from a human adult T cell that carried a T cell receptor (TCR) recognizing the Epstein-Barr virus (EBV) peptide FLYALALLL, which is encoded by the EBV gene *LMP2* and is presented by HLA-A^∗^02:01.^147^ After delivery of the reprogramming factors, the resultant “T-hiPSCs” (a term referring to hiPSCs originally generated from a T cell) encoded a rearranged, EBV-specific TCR. In a previous report, the iLC13-F1 hiPSC line was named “EBV-iPS”.^147^

H7 *CAG-AkaLuciferase-PuroR* hESCs were generated as part of this study. PiggyBac transposition was used to deliver a PiggyBac (*pPB-CAG-AkaLuciferase-PuroR*) construct^190^ into H7 hESCs. *AkaLuciferase* encodes a luciferase variant optimized for highly sensitive intravital bioluminescent imaging, which affords the capability to detect single cells *in vivo* under certain circumstances.^168^

Wild-type WTC11 hiPSCs have been described previously.^148^ Human skin fibroblasts were transfected with episomal plasmids encoding the reprogramming factors.^148^ WTC11 hiPSCs are of an XY genotype and Japanese ethnicity (https://www.coriell.org/0/Sections/Search/Sample_Detail.aspx?Ref=GM25256).

#### Feeder cells

OP9-DLL4*-IRES-GFP* feeder cells^149^ were provided courtesy of Mahmood Mohtashami and Juan Carlos Zúñiga-Pflücker, and were maintained in αMEM (Thermo Fisher, 12-571-063) supplemented with 20% FBS (R&D Systems, S11550) and 1% penicillin/streptomycin (Thermo Fisher, 15-140-122). These feeder cells constitutively express both the NOTCH ligand *DLL4* and a fluorescent GFP reporter.^149^

MS5 feeder cells (DSMZ) were maintained in αMEM (Thermo Fisher) supplemented with 10% FBS (R&D Systems) and 1% penicillin/streptomycin (Thermo Fisher).

#### Human cord blood hematopoietic stem and progenitor cells

Human CD34+ cord blood hematopoietic stem and progenitor cells (HSPCs) were obtained from de-identified donors and were magnetically enriched using CD34 magnetic-activated cell sorting (MACS) by StemExpress (CB3400.5C).

#### Mouse models

*Cx40-CreERT2* mice have been described previously,^77^ and were provided by Lucile Miquerol’s laboratory. In these mice, the endogenous coding sequence of the *Cx40* gene was replaced by a *CreERT2-IRES-RFP*-*PGK-NeomycinR* cassette.^77^ These mutant mice were maintained heterozygously (i.e., *Cx40*^*CreERT2/+*^) on a CD1 background. These mice were homozygous for the CD45.2 antigen.

*Efnb2-CreERT2* mice were generated as part of this study, by Hong Zeng, Charlene Wang, and the Stanford Transgenic, Knockout, and Tumor Model Center. In these mice, the endogenous *Efnb2* gene was edited to replace the *Efnb2* stop codon with a *GSG-P2A-CreERT2-F5* cassette, as described more fully in the “Construction of *Efnb2-CreERT2* mice” section. In brief, a GSG-P2A linker was chosen, owing to the high translational skipping efficiency afforded by this linker^191^; the GSG sequence (preceding P2A) was published previously.^192^ A single F5 site was also inserted downstream of *CreERT2*. This approach theoretically preserves the coding sequence of the endogenous *Efnb2* gene. These mutant mice were maintained heterozygously (i.e., *Efnb2*^*CreERT2/+*^) on a C57BL/6 background. For reasons that are not fully understood, homozygous *Efnb2*^*CreERT2/CreERT2*^ mice could be not obtained; moreover, heterozygous mutant mice also exhibited certain difficulties in breeding. *Efnb2-CreERT2* mice are being deposited at The Jackson Laboratory (JAX strain number 038831).

*Apj-CreERT2* mice have been described previously,^91^ and were provided by Kristy Red-Horse’s laboratory. In these mice, a bacterial artificial chromosome (BAC) containing *Apj* (otherwise known as *Aplnr*) was genetically edited to replace the *Apj* gene with *CreERT2*, and the resultant BAC was randomly integrated into the mouse genome.^91^ *Apj-CreERT2* mice were maintained heterozygously (i.e., *Apj*^*CreERT2/+*^) on an FVB/NJ background, with regular genotyping performed to confirm the presence of *CreERT2*.

*Rosa26-CAG-LoxP-Stop-LoxP-ZsGreen* mice (otherwise known as “Ai6”) have been described previously^78^; they were originally developed by the Allen Brain Institute and were provided through the intermediacy of The Jackson Laboratory (JAX 007906). In these mice, a *CAG-LoxP-Stop-LoxP-ZsGreen* allele was inserted into the endogenous *Rosa26* safe harbor locus, such that Cre-driven recombination leads to the stable expression of the *ZsGreen* fluorescent reporter.^78^

C57BL/6-CD45.2 and C57BL/6-CD45.1 (Pepboy) mice were obtained from The Jackson Laboratory (JAX 000664 and JAX 002014, respectively) and bred in-house. C57BL/6-CD45.2 and C57BL/6-CD45.1 were crossed to yield heterozygous mice (CD45.2+ CD45.1+). For mouse HSC transplant experiments, 6- to 12-week-old mice were used as recipients.

NOD-SCID Il2rg^-/-^ (NSG) mice^150^ were obtained from The Jackson Laboratory (JAX 005557) and bred in-house. For human hematopoietic stem and progenitor cell transplant experiments, male and female 8- to 12-week-old NSG mice were used as recipients.

### Method details

#### Mouse husbandry and lineage tracing

Mice of the desired genetic backgrounds were mated to generate timed pregnancies. Noon on the day a vaginal plug was detected was designated as embryonic day 0.5 (E0.5). For lineage tracing, 25 mg of (*Z*)-4-hydroxytamoxifen (4OHT; Sigma, H7904) was dissolved in 1250 μL ethanol (Fisher Scientific, BP2818500) by vortexing and heating at 60 °C to create a 20 mg/mL stock, which was aliquoted and stored at -20 °C. 50 μL aliquots (containing 1 mg of 4OHT) were heated for 10 minutes at 65 °C before being mixed with pre-warmed corn oil (250 μL, Sigma, C8267). The 4OHT/corn oil mixture was thoroughly vortexed to mix it, before delivery via intraperitoneal injection to pregnant female mice at the relevant labeling timepoint. 1 mg of 4OHT was injected per mouse.

Raw data for all lineage tracing experiments is tabulated in Table S1. For all lineage tracing experiments that analyzed cell contribution to embryonic lineages, we analyzed at least 8 independent embryos from at least 3 independent litters per timepoint. Each dot on the bar charts represents an independent litter (Figures 1K–1M and S2G).

#### Transplantation of mouse HSCs into recipient mice

Male and female 8- to 12-week-old C57BL/6 mice, of either a CD45.1 (Pep Boy) or CD45.1/CD45.2 genotype, were used as recipient mice. These C57BL/6 mice received a split dose of irradiation (two doses of 6.5Gy, separated by 4 hours) for a total dosage of 13Gy. 1-5 million cells (of a CD45.2 genotype) were transplanted by retro-orbital injection. Transplanted cells consisted of either dissociated fetal liver cells (primary transplant) or dissociated bone marrow cells (secondary transplant).

#### Flow cytometry analysis of mouse adult peripheral blood

Peripheral blood of adult mice was collected and analyzed to detect the percentage of fluorescently labeled blood and immune cells. In brief, mice were anesthetized with isoflurane, and then retroorbital blood was drawn and placed into an EDTA-coated Microtainer tube (BD Biosciences, 365974). 1 μL of blood was set aside to analyze red blood cells and platelets and was stained using antibodies diluted in FACS buffer (PBS + 2% FBS + 1% Penicillin/Streptomycin + 0.5 mM EDTA).

Separately, erythrocytes were depleted from the remainder of the blood sample by 45 minutes of dextran sedimentation (Sigma, 31392), followed by two rounds of lysis in ACK (Ammonium-Chloride-Potassium) Lysing Buffer (for 5 minutes each; Thermo Fisher, A1049201), and then cells were stained using antibodies diluted in FACS buffer.

Within mouse peripheral blood, we identified the following cell-types using various antibodies:

• CD45+ hematopoietic cells (PE-Cy7 Anti-mouse CD45 antibody, Biolegend, 103114)
• Gr1+/Mac1+ myeloid cells (PE Anti-mouse GR-1/Ly-6G antibody, Biolegend, 108408 and PE Anti-mouse Mac-1/CD11b antibody, Biolegend, 101208)
• CD4+/CD8+ T cells (APC Anti-mouse CD4 antibody, Biolegend, 100516 and APC Anti-mouse CD8a antibody, Biolegend, 100712)
• B220+ B cells (APC-Cy7 Anti-mouse B220/CD45R antibody, Biolegend, 103224)
• CD41+ platelets (Brilliant Violet 421 anti-mouse CD41 antibody, Biolegend, 133912)
• TER119+ red blood cells (PE-Cy5 anti-mouse TER119 antibody, Thermo Fisher, 15-5921-83)

Flow cytometric analysis was performed on a BD FACSAria II SORP.

#### *In situ* hybridization of whole mount mouse embryos

Hybridization chain reaction v3.0 (HCR3)^79^ was used to perform whole-mount fluorescent *in situ* hybridization (FISH) of mouse embryos. For HCR3, embryos were dissected in ice-cold 4% paraformaldehyde (diluted from Fisher Scientific, 50-980-495), and subsequently fixed overnight prior to methanol dehydration (Fisher Scientific, A412-1). Hybridization mRNA probes, amplifiers, and buffers were obtained from Molecular Instruments. Staining of whole-mount mouse embryos was performed as per the Molecular Instruments protocol (https://www.molecularinstruments.com/hcr-rnafish-protocols). Embryos were incubated in DAPI + SSCT (sodium chloride, sodium citrate, and Tween buffer) prior to mounting.

#### Immunohistochemistry of mouse embryo sections

Embryos were dissected, fixed in 4% paraformaldehyde overnight at 4 °C, washed in PBS overnight at 4 °C, cryoprotected with 30% sucrose for 24-48 hours (until the tissue sank), and embedded in O.C.T. solution. They were then sectioned to a thickness of 16-20 microns and then stained as described previously.^193^ In brief, slides were washed for 3 x 10 minutes in PBS at room temperature, then permeabilized and blocked in blocking buffer (PBS + 0.1% Triton X-100 + 5% donkey serum) for 1 hour at room temperature. They were then incubated overnight in primary antibodies diluted in PBS + 0.1% Triton X-100 + 1% donkey serum in a humidified chamber at 4C. The following day, slides were washed 3 x 10 minutes in PBS at room temperature and stained with Alexa Fluor-conjugated secondary antibodies diluted in PBS + 0.1% Triton X-100 + 1% donkey serum in a humidified chamber at room temperature. Slides were then washed 3 x 5 minutes in PBS, stained with DAPI, mounted in ProLong Gold anti-fade reagent (Thermo Fisher, P36930), and then cured for 24 hours at room temperature. Z-stacks of optical sections were captured on an Olympus FV3000 confocal microscope. Data were displayed using FIJI/ImageJ software.^152^

#### Flow cytometry analysis of mouse embryo dorsal aorta, yolk sac, and fetal liver and mouse adult bone marrow

Mouse embryos were dissected to obtain dorsal aorta, yolk sac, or fetal liver, as described previously.^194^ For dorsal aorta and yolk sac analyses, tissues were dissociated in collagenase for 30-90 minutes at 37 °C before being stained by antibodies diluted in FACS buffer (PBS + 2% FBS + 1% Penicillin/Streptomycin + 0.5 mM EDTA).^194^ For fetal liver analyses, tissues were passed through a 100 micron filter, washed twice in FACS buffer, and passed through a 40 micron filter before being stained by antibodies diluted in FACS buffer.

Adult mouse bone marrow was analyzed as previously described, either through intrafemoral aspirates of living mice^195^ or alternatively, by sacrificing mice and crushing bones to isolate bone marrow.^196^

We identified by CD144+ CD45+ hematopoietic stem and progenitor cells in the yolk sac and dorsal aorta^46^ by staining with Alexa Fluor 647 anti-mouse CD144 antibody (BD Biosciences, 562242) and V450 anti-mouse CD45 antibody (BD Biosciences, 560501).

As described previously,^196^ we defined HSCs in the adult mouse bone marrow and fetal liver as CD150+ CD48- Lineage- Sca1+ Kit+ (LSK).^87^^,^^197^ The following antibodies were employed to discriminate HSCs: BV421 anti-mouse CD48 antibody (Biolegend, 103428), PE anti-mouse Sca-1 antibody (Biolegend, 108108), PE-Cy7 anti-mouse CD150 antibody (Biolegend, 115914), and APC anti-mouse c-Kit/CD117 antibody (Biolegend 313205). Exclusion using the following cocktail of “lineage” antibodies was also used to identify HSCs: we stained with biotinylated primary antibodies directed to Ter119, CD4, CD8, CD127/IL7R, B220, and Gr1 (Biolegend, 116204, 100508, 100704, 135006, 103204, and 108404, respectively), followed by secondary staining with APC-eFluor 780 Streptavidin (Thermo Fisher, 47-4317-82).^196^

Flow cytometry was performed on a BD FACSAria II SORP and data were analyzed with FlowJo.

#### Adult mouse brain dissociation

Brain tissue was dissected from adult mice, and then gently minced with a razor blade to yield ∼1 mm^3^ chunks. The minced tissue was resuspended in Hank’s balanced salt solution (HBSS) with Ca^2+^/Mg^2+^ (Thermo Fisher, 24020117) with 10 μg/mL Liberase (Roche, 5401119001) and 200 μg/mL DNase I (Worthington, cat. #LS002007), then incubated at 37°C for 30 minutes under constant agitation. Afterwards, samples were triturated by pulling the cell suspension through a 10 mL serological pipette fitted with a 1000 μL pipette tip for 10-15 strokes or until it passed through smoothly. The cell suspension was spun down and resuspended in Accutase (Innovative Cell Technologies, AT104) supplemented with 200 μg/mL DNase I, then incubated at 25 °C for 15 minutes under constant agitation. Myelin debris removal was performed using a Percoll density gradient (Cytiva, 17-0891-02): cells were resuspended in 30% Percoll and centrifuged at 300g for 15 min at 25°C with no brakes. The floating myelin layer and supernatant were removed, and the resulting cell pellet was used for antibody staining. All washes and staining were performed in HBSS without Ca^2+^/Mg^2+^ (Thermo Fisher, 14175103) supplemented with 0.1% polyvinyl alcohol (PVA) (Sigma, P8136).

#### Adult mouse liver dissociation

Liver tissue was dissected from adult mice and then gently minced with a razor blade. The minced tissue was resuspended in M199 medium (Thermo Fisher, 11150059) with 2.2% Type II Collagenase (Thermo Fisher, 17101015) and 0.2% DNase I (Worthington, LS002007), and then incubated at 37°C for 30 minutes under constant agitation. Afterwards, samples were triturated with a 10 mL serological pipette 10-15 times. The cell suspension was then passed through a 100-micron filter and centrifuged. Cells were resuspended in PBS (Thermo Fisher, 10010001) supplemented with 1% FBS and 2 mM EDTA (Thermo Fisher, 15575020). CD45+ cell enrichment was performed by incubating cells with anti-mouse CD45 MicroBeads (Miltenyi, 130-052-301) at 4°C for 15 minutes. Cells were washed twice and passed through LS Columns (Miltenyi, 130-042-401) on a MACS Separator. Columns were removed from the magnetic stand and plunged to positively select CD45+ cells.

#### Adult spleen dissociation

Spleen tissue was dissected from adult mice and transferred to a cell culture dish containing FACS buffer (PBS supplemented with 1% FBS and 2 mM EDTA) on ice. 100-micron strainers were placed on 50 mL conical tubes, and then these strainers were then washed (primed) with 2 mL FACS buffer. Spleens were then placed on the primed strainers and were gently crushed using the flat end of sterile 3 mL syringe plunger by pressing the spleens in a circular motion. Strainers were washed with an additional 5 mL FACS buffer and cells were pelleted by centrifugation.

#### Flow cytometry analysis of adult mouse brain, liver, and spleen

Dissociated brain, liver, and spleen cells were washed two times with PBS supplemented with 1% FBS and 2 mM EDTA prior to counting and staining. All samples were then Fc blocked with TruStain FcX anti-mouse CD16/32 (Biolegend, 101320) for 5 minutes prior to staining. Cells were then stained for 30 minutes on ice with anti-mouse CD45 (Biolegend, 103149), anti-mouse/human CD11b (Biolegend, 101242), anti-mouse F4/80 (Biolegend, 123149), anti-mouse CX3CR1 (Biolegend, 149048), and anti-mouse CD206 (Biolegend, 141717) antibodies. Samples were washed two times with PBS supplemented with 1% FBS and 2 mM EDTA before analyzing. Sytox Red Dead Cell Stain was added (Thermo Fisher, S34859) to each sample before analysis. Flow cytometry data were analyzed with FlowJo.

Cell populations of interest were defined using previously published combinations of cell-surface markers. We defined the following myeloid cell-types within the liver as described previously^198^:

• Kupffer cells: CD45+ F4/80^high^ CD11blow CX3CR1-
• Liver capsular macrophages: CD45+ F4/80^high^ CD11b^low^ CX3CR1+
• Monocyte-derived macrophages: CD45+ F4/80^lo^ CD11b^hi^ CX3CR1+
• Monocytes as CD45+ F4/80- CD11b^hi^ CX3CR1+
• Neutrophils/dendritic cells: CD45+ F4/80- CD11b^hi^ CX3CR1-

Within the brain, microglia were defined as CD45^low^ CD11b+ F4/80^high^ CX3CR1+, as described previously.^96^^,^^199^^,^^200^

Within the spleen, splenic macrophages were defined as CD45+ F4/80+, as described previously.^96^

#### Liquid chromatography with tandem mass spectrometry

Liquid chromatography with tandem mass spectrometry (LC/MS-MS) was performed on a Waters Xevo TQ-XS mass spectrometer system, as described previously.^201^ Standards were prepared using CD1 mouse plasma spiked with E/Z-4-hydroxytamoxifen-d5 internal standard (Cayman Chemicals, 34232). Peripheral blood was obtained from adult mice through retroorbital blood draws and stored in EDTA-coated Microtainer tubes (BD Biosciences, 365974). Blood samples were centrifuged for 2000g for 15 minutes at 4 °C to separate the plasma (supernatant), which was aliquoted and frozen at -20 °C before mass spectrometric analysis.

#### Basement membrane matrices

hPSCs were maintained and differentiated on cell culture plates that been pre-coated with basement membrane matrices. To coat cell culture plates, a volume of basement membrane matrix solution was added that was roughly equivalent to half the working volume of the well or dish (e.g., 1 mL, 0.5 mL, or 0.3-0.35 mL of basement membrane matrix solution was added per well of a 6-well, 12-well, or 24-well plate, respectively).

Propagation of undifferentiated hPSCs, as well as hPSC differentiation into posterior primitive streak, lateral mesoderm, and artery endothelium cells (as described below), was conducted on Geltrex-coated plates. As described previously,^104^ Geltrex (Thermo Fisher, A1413302) was diluted 1:100 in DMEM/F12 (Thermo Fisher, 10565042) and was used to coat tissue culture plastics for at least 1 hour at 37 °C. Afterwards, Geltrex was aspirated, leaving behind a thin film, and cells were then plated.

Differentiation of hPSCs into hemogenic endothelium cells and HLF+ HOXA+ hematopoietic progenitors (as described below) was conducted on plates that had been coated with vitronectin and the high-affinity, engineered NOTCH agonist DLL4-E12 (Luca et al.^125^). Recombinant human truncated vitronectin (Thermo Fisher, A14700; “VTN-N”) was diluted to a 10 μg/mL stock in PBS, and 20 nM of DLL4-E12 (from Vincent Luca’s laboratory^125^) was then added. This DLL4-E12/vitronectin solution then used to coat cell culture plates for at least 1 hour at 37 °C. Afterwards, the DLL4-E12/vitronectin solution was aspirated, and plates were gently washed three times with PBS to remove any soluble DLL4-E12, which would instead strongly inhibit NOTCH signaling.^125^ After washing was completed, cells were then plated.

#### Culture and passaging of undifferentiated hPSCs

All undifferentiated hPSCs utilized in this study (with the exception of WTC11 hPSCs) were routinely propagated in mTeSR Plus medium (STEMCELL Technologies) + 1% penicillin/streptomycin, as described previously.^104^ For concision, mTeSR Plus + 1% penicillin/streptomycin is referred to as “mTeSR” throughout the remainder of this document. WTC11 hPSCs were propagated in E8 (Thermo Fisher) + 1% penicillin/streptomycin.

When they became partially or largely confluent, undifferentiated hPSCs were passaged for maintenance by treating them for 5-7 minutes with EDTA solution (Versene; Thermo Fisher, 15040066) at room temperature, after which EDTA was removed, mTeSR was added, and then hPSCs were manually scraped off the plate with a scraper to generate clumps. hPSC clumps were then seeded onto new Geltrex-coated plates that contained mTeSR. For WTC11 hPSCs, the same procedure was performed, except that they were scraped and seeded in E8 medium.

#### Preparation of CDM2 and CDM3 basal media for differentiation

The composition of CDM2 has been described previously^103^^,^^104^^,^^123^: 50% IMDM + GlutaMAX (Thermo Fisher, 31980-097) + 50% F12 + GlutaMAX (Thermo Fisher, 31765-092) + 1 mg/mL polyvinyl alcohol (Sigma, P8136-250G) + 1% v/v chemically defined lipid concentrate (Thermo Fisher, 11905-031) + 450 μM 1-thioglycerol (Sigma, M6145-100ML) + 0.7 μg/mL recombinant human insulin (Sigma, 11376497001) + 15 μg/mL human transferrin (Sigma, 10652202001) + 1% v/v penicillin/streptomycin (Thermo Fisher, 15070-063).

The composition of CDM3 has been described previously^202^: 45% IMDM + GlutaMAX (Thermo Fisher, 31980-097) + 45% F12 + GlutaMAX (Thermo Fisher, 31765-092) + 10% KnockOut Serum Replacement (Thermo Fisher, 10828028) + 1 mg/mL polyvinyl alcohol (Sigma, P8136-250G) + 1% v/v chemically defined lipid concentrate (Thermo Fisher, 11905-031) + 1% v/v penicillin/streptomycin (Thermo Fisher, 15070-063).

To prepare CDM2 and CDM3, first polyvinyl alcohol was suspended in either IMDM or F12 by gentle warming and magnetic stirring. After polyvinyl alcohol was dissolved, the remaining media components were mixed together, and the complete media was sterilely filtered through a 0.22 μm filter prior to use.

#### Resuspension of recombinant growth factor proteins and small molecules

We observed the following practices regarding the growth factors and small molecules employed below for hPSC differentiation. Small molecules were typically resuspended in DMSO to generate 10 mM stocks, which were frozen in a -20 °C freezer. Recombinant growth factor proteins were typically resuspended in PBS + 0.1% BSA Fraction V (Thermo Fisher, 15260-037) to generate 50-100 μg/mL stocks, which were frozen in a -20 °C freezer. The PBS + 0.1% BSA Fraction V solution was sterilely filtered before being used to resuspend growth factor proteins.

As needed, small molecule and growth factor stocks were thawed, added to CDM2 or CDM3 differentiation media (as described below), and then any remaining amount of the stock was re-frozen.

#### hPSC differentiation into HLF+ HOXA+ hematopoietic progenitors

In brief, undifferentiated hPSCs were dissociated using Accutase (Thermo Fisher, 00-4555-56) and sparsely seeded as single cells prior to commencing differentiation. Sparse seeding of single cells was crucial for efficient differentiation. To reiterate, undifferentiated hPSCs were maintained by passaging them as small clumps (using EDTA-based dissociation, to maintain normal karyotype [as described above]), but were seeded for differentiation as single cells (using Accutase-based dissociation; to enable efficient differentiation).^104^ The initial steps of differentiation (posterior primitive streak, lateral mesoderm, and artery endothelium induction) were conducted in CDM2 basal media.

• **Seeding hESCs for differentiation** (**Step 0**). Largely-confluent hPSCs were dissociated into single cells using Accutase (Thermo Fisher), and counted. They were then plated into recipient plates that had been precoated with Geltrex (as described above [Thermo Fisher, A1413302]) in mTeSR supplemented with thiazovivin (1 μM, a ROCK inhibitor, to enhance hPSC survival after passaging [Tocris, 3845]). ∼30,000-50,000 hPSCs/cm^2^ were seeded (i.e., ∼1.2-2x10^5^ hPSCs/well of a 12-well plate or ∼1.5-2.75x10^6^ hPSCs per 10-cm dish). For any given hPSC line, we tested a range of initial seeding densities in order to determine the best day 0 seeding density to achieve the highest purity of day-4 CD144+ DLL4+ artery endothelial cells. This optimization of initial seeding densities was critical to ensure efficient differentiation, as different hPSC lines may exhibit slightly different proliferation rates. Freshly-seeded hPSCs were allowed to adhere and recover for 24 hours in mTeSR + 1 μM thiazovivin prior to initiating differentiation, during which the hPSCs re-formed small clumps.
• **Day 1-2** (**posterior primitive streak induction, 48 hours**) (**Step 1**). Day 0 hPSCs were briefly washed (DMEM/F12 [Thermo Fisher, 10565042]) to remove all traces of mTeSR + thiazovivin. Then, they were differentiated towards posterior primitive streak in CDM2 media supplemented with BMP4 (40 ng/mL [R&D Systems, 314-BP-050]), CHIR99021 (6 μM [Tocris, 4423]), and FGF2 (20 ng/mL [R&D Systems, 233-FB-01M]) for 48 hours. Posterior primitive streak induction media was refreshed every 24 hours.
• **Day 3** (**lateral mesoderm induction, 24 hours**) (**Step 2**). Day 2 posterior primitive streak cells were briefly washed (DMEM/F12) and then differentiated towards lateral mesoderm in CDM2 media supplemented with BMP4 (40 ng/mL), GDC-0941 (2.5 μM [Cellagen Technology, C4321-25]), Forskolin (10 μM [Tocris, 1099]), SB505124 (2 μM [Tocris, 3263]), VEGF (100 ng/mL [R&D Systems, 293-VE-0500]), XAV939 (1 μM [Tocris, 3748]), AA2P (200 μg/mL [Sigma, 49752-10G]), and TTNPB (0.5 nM [Tocris, 0761]) for 24 hours.
• **Day 4** (**artery endothelium induction, 24 hours**) (**Step 3**). Day 3 lateral mesoderm cells were briefly washed (DMEM/F12) and then differentiated towards artery endothelial cells in CDM2 media supplemented with Activin A (15 ng/mL [R&D Systems, 338-AC-500/CF]), DMH1 (250 nM [Tocris, 4126]), GDC-0941 (2.5 μM), VEGF (100 ng/mL), XAV939 (1 μM), AA2P (200 μg/mL), and TTNPB (0.5 nM) for 24 hours.

Subsequently, hPSC-derived day 4 artery endothelial cells were dissociated into single cells (using Accutase), counted, and then re-seeded at high density (∼500,000 cells/cm^2^; i.e., ∼1x10^6^ cells/well of a 24-well plate) on plates that had been coated with 10 μg/mL vitronectin (Thermo Fisher, A14700) + 20 nM of the high-affinity NOTCH agonist DLL4-E12 (from Vincent Luca’s laboratory^125^). ∼300-350 μL of vitronectin/E12 solution was used to coat each well of a 24-well plate, using the method described in a preceding section. At this stage, high cellular seeding density was critical to subsequently achieve efficient blood differentiation. The next steps of differentiation (hemogenic endothelium and HLF+ HOXA+ hematopoietic progenitor induction) were conducted in CDM3 basal media.

• **Day 5-7** (**hemogenic endothelium induction, 72 hours**) (**Step 4**). Day 4 artery endothelial cells cells were dissociated into a single-cell suspension (Accutase); densely re-seeded at 500,000 cells/cm^2^ onto plates precoated with 10 μg/mL Vitronectin + 20 nM of high-affinity NOTCH agonist DLL4-E12; and then further differentiated towards hemogenic endothelium in CDM3 media supplemented with Forskolin (10 μM), LIF (20 ng/mL [R&D Systems, 7734-LF-025]), OSM (10 ng/mL [R&D Systems, 295-OM-010]), SB505124 (2 μM), and UNC1999 (1 μM [Tocris, 4904]) for 72 hours. Hemogenic endothelium induction media was refreshed every 24 hours with a complete media change.
• **Day 8-10** (**HLF+ HOXA+ hematopoietic progenitor induction, 72 hours**) (**Step 5**). Day 7 hemogenic endothelium cells were differentiated towards HLF+ HOXA+ hematopoietic progenitors in CDM3 media supplemented with Forskolin (10 μM), SB505124 (2 μM), SR1 (750 nM [Cellagen, C7710-5]) and UM171 (75 nM [ApexBio, A8950]), UNC0638 (500 nM [Tocris, 4343]), and UNC1999 (1 μM) for 72 hours. No wash was performed when adding media to avoid disturbing the emerging semi-adherent cells. Hematopoietic progenitor induction media was refreshed every 24 hours with a complete media change for day 8 and 9, but on the last day (day 10), media was supplemented only.

Two different methods were used to dissociate and collect day 10 HLF+ HOXA+ hematopoietic progenitors. First, they were dissociated with TrypLE (Thermo Fisher, 12604013) for 3-5 minutes at 37°C and gentle trituration was performed. Alternatively, for more gentle dissociation, they were incubated in Papain (0.5 mg/mL; Worthington Biochemical, LS003118) dissolved in PBS + 1.1 mM EDTA (Thermo Fisher, 15575020) + 0.067 mM 2-mercaptoethanol (Gibco, 21985023) + 5.5 mM Cysteine-HCl (Sigma, C7477) for 30-45 minutes at 37 °C and gentle trituration was performed. HLF+ HOXA+ hematopoietic progenitors were then counted, washed (DMEM/F12, Thermo Fisher) and prepared as needed for downstream assays.

#### Designing the protocol to differentiate hPSCs into HLF+ HOXA+ hematopoietic progenitors: Rationale and design considerations

Here we outline how we systematically optimized the differentiation of hPSCs into HLF+ HOXA+ hematopoietic progenitors (described above) and the underlying rationale. At each differentiation step, we systematically screened activators and inhibitors of various developmental signaling pathways, including BMP, FGF, NOTCH, RA, TGFβ, and WNT in a serum-free medium, and assessed the impact of each perturbation on marker gene expression. At later differentiation steps, we also tested pharmacological inhibitors of various chromatin regulators. The optimal timing for each differentiation step was determined as described below.

The differentiation strategy from days 0-4 was modified from our published work describing the stepwise differentiation of hPSCs into mid primitive streak, lateral mesoderm, and artery endothelial cells.^104^ In this study, the following modifications were made: (1) use of posterior (instead of middle) primitive streak, (2) elongation of posterior primitive streak differentiation from 1 day to 2 days, and (3) addition of RA agonist during lateral mesoderm and artery endothelial differentiation steps. Days 0-4 of differentiation were conducted in CDM2 basal media, as previously described.^104^

• **Day 1–2** (**posterior primitive streak induction, 48 hours**) (**Step 1**). We activated BMP (which induces posterior primitive streak), without providing exogenous TGFβ (which induces anterior primitive streak)^103^^,^^123^ (Figure 4B). Alongside BMP, we also activated FGF and WNT (Figure 4B), as both FGF and WNT are required for pan-primitive streak induction.^103^^,^^123^ The duration of the primitive streak stage (2 days) was empirically determined through experimentation. 2 days of posterior primitive streak differentiation were crucial to induce *HOXA5-HOXA10* within primitive streak cells, whereas 1 day was not sufficient (Figure 4Ci). Additionally, 2 days of posterior primitive streak differentiation were crucial to subsequently generate HLF+ hematopoietic progenitors, whereas 1 day was not sufficient (Figure 4Fiii).
• **Day 3** (**lateral mesoderm induction, 24 hours**) (**Step 2**). The duration of, and signals used during, this 1-day differentiation step were largely based on our past work to differentiate hPSC-derived primitive streak into lateral mesoderm.^104^ However, we additionally activated the RA pathway at this stage, which promoted the expression of certain anterior *HOXA* genes (Figure S5Iii).
• **Day 4** (**artery endothelium induction, 24 hours**) (**Step 3**). The duration of, and signals used during, this 1-day differentiation step were largely based on our past work to differentiate hPSC-derived primitive streak into lateral mesoderm.^104^ However, we additionally activated the RA pathway at this stage, which promoted the expression of certain anterior *HOXA* genes (Figure S5Iiv).

Having generated day 4 artery ECs, we then optimized their subsequent differentiation into hemogenic ECs, and finally, HLF+ hematopoietic progenitors. Multiple considerations were taken into account when optimizing these differentiation steps, and here we emphasize three specific points. First, as described above, we dissociated artery endothelial cells and seeded them onto new plates coated with high-affinity NOTCH agonist DLL4-E12 (Luca et al.^125^), given that NOTCH signaling is necessary for HSC development *in vivo*.^178^^,^^180^ Second, we found that seeding artery ECs at high density (∼500,000 cells/cm^2^) was critical to efficiently generate hematopoietic progenitors at later differentiation steps (Figure S6F). Third, the following differentiation steps were conducted in CDM3 basal media, which contains KnockOut Serum Replacement, which we found to enhance hematopoietic progenitor generation (not shown).

• **Day 5-7** (**hemogenic endothelium induction, 72 hours**) (**Step 4**). Through systematic screening, we found that the simultaneous modulation of five signaling pathways at this stage was important to differentiate artery endothelium into hemogenic endothelium: (1) GP130 pathway activation (LIF and OSM), (2) cAMP/PKA pathway activation (Forskolin), (3) TGFβ inhibition (SB505124), (4) PRC2 inhibition (UNC1999), and (5) NOTCH activation (DLL4-E12). First, we activated GP130 signaling, which is known to drive mouse and zebrafish hematopoietic development.^128^^,^^129^^,^^130^ Second, we activated cAMP/PKA signaling, which is known to upregulate *Runx1* in response to prostaglandin E2^132^ and shear stress.^131^ Third, we inhibited TGFβ signaling, consistent with how TGFβ inhibitors enhance hematopoietic specification in various contexts.^203^^,^^204^^,^^205^ Fourth, we inhibited PRC2, as deletion of the PRC2 methyltransferase *Ezh1* leads to precocious HSC emergence *in vivo*.^137^ Fifth, we activated NOTCH, because NOTCH signaling is strictly required for HSC formation *in vivo*.^178^^,^^180^ Regarding timing: 3 days of differentiation is optimal to achieve efficient conversion of artery ECs into RUNX1+ CD144+ hemogenic endothelium cells, as shown by timecourse analysis of RUNX1-mOrange expression (Figure S6A).
• **Day 8–10** (**HLF+ HOXA+ hematopoietic progenitor induction, 72 hours**) (**Step 5**). Through systematic screening, we found that the simultaneous modulation of seven signaling pathways at this stage was important to differentiate hemogenic endothelium into HLF+ HOXA+ hematopoietic progenitors: (1) cAMP/PKA pathway activation (Forskolin), (2) TGFβ inhibition (SB505124), (3) PRC2 inhibition (UNC1999), (4) NOTCH activation (DLL4-E12), (5) G9A/GLP inhibition (UNC0638), (6) aryl hydrocarbon receptor inhibitor (SR1), and (7) LSD1 inhibitor (UM171). The rationale for cAMP/PKA activation, TGFβ inhibition, PRC2 inhibition, and NOTCH activation was described above. We employed an aryl hydrocarbon inhibitor (SR1),^140^ an LSD1 inhibitor (UM171),^138^^,^^139^ and a G9A/GLP inhibitor (UNC0638),^141^^,^^142^ because all of these are thought to stabilize undifferentiated HSCs *ex vivo* and block their spontaneous differentiation into downstream progeny.^138^^,^^139^^,^^140^^,^^141^^,^^142^ Indeed, by day 10 of hPSC differentiation, IL7R+ lymphoid or GATA1+ erythroid progenitors were not detected (Figure S6Mii), suggesting that we had prevented the precocious differentiation of day-10 hPSC-derived HLF+ HOXA+ hematopoietic progenitors into lymphoid or erythroid progenitors. Regarding timing: 3 days of differentiation is optimal to achieve efficient conversion of hemogenic ECs into HLF+ hematopoietic progenitors, as shown by timecourse analysis of *HLF* expression (Figure S6G).

#### Differentiation of hPSC-derived HLF+ HOXA+ hematopoietic progenitors into myeloid and erythroid cells in methylcellulose culture

Day 10 hPSC-derived HLF+ HOXA+ hematopoietic progenitors were collected, counted, and cultured in methylcellulose (MethoCult H4435 Enriched, STEMCELL Technologies, 04435) with 1.6x10^3^ cells per 35mm dish prepared in triplicate. Cultures were incubated at 37 °C for 14 days and then manually counted to identify each colony forming unit (CFU) containing granulocyte, erythroid, monocyte, and megakaryocytes (CFU-GEMM), granulocyte and monocyte cells (CFU-GM), erythroid cells (CFU-E), as well as burst forming unit-erythroid (BFU-E).

#### Differentiation of hPSC-derived HLF+ HOXA+ hematopoietic progenitors into erythroid cells

Erythroid differentiation was performed using the StemSpan Erythroid Expansion Supplement (STEMCELL Technologies, 02692) following the manufacturer’s recommendations. In brief, day 10 hPSC-derived HLF+ HOXA+ hematopoietic progenitors were collected, counted, and seeded in StemSpan SFEM II (STEMCELL Technologies, 09655) supplemented with the StemSpan Erythroid Expansion Supplement at a density of 1x10^4^-1x10^5^ cells/mL. On day 3, cells were supplemented with an equal volume of complete media. On day 7 and 10, cells were harvested and replated in complete media at a density of 1x10^5^ cells/mL. On day 14, cells were harvested and processed for flow cytometry.

High-performance liquid chromatography (HPLC) to detect hemoglobin tetramers was performed as previously described.^206^

#### Differentiation of hPSC-derived HLF+ HOXA+ hematopoietic progenitors into macrophages

Day 10 hPSC-derived HLF+ HOXA+ hematopoietic progenitors were collected, counted, and seeded in StemPro-34 base media (Thermo Fisher) supplemented with the following factors:

• Day 0-5: SCF (50 ng/mL [Peprotech, 300-07]), TPO (10 ng/mL [Peprotech, 300-18]), IL-3 (50 ng/mL [Peprotech, 200-03]), FLT3L (50 ng/mL [Peprotech, 300-19]), M-CSF (50 ng/mL [Peprotech, 300-25]), and ITS-X (Thermo Fisher, 51500-056])
• Day 6-10: FLT3L (50 ng/mL), M-CSF (50 ng/mL), GM-CSF (25 ng/mL [Peprotech, 300-03]), and ITS-X
• Day 10-17: M-CSF (100 ng/mL), GM-CSF (50 ng/mL), and ITS-X

At day 17, cells were dissociated using TrypLE (Thermo Fisher), stained with CD11b APC antibody (BioLegend, 101212) and CD68 PE Cy7 antibody (Thermo Fisher, 25-0689-42) in the presence of human Fc blocking reagent (Miltenyi Biotec, 130-059-901), and analyzed for flow cytometry.

#### Differentiation of hPSC-derived HLF+ HOXA+ hematopoietic progenitors into T cells

T cell differentiation was performed using the StemSpan T Cell Generation Kit (STEMCELL Technologies, 09940) following the manufacturer’s recommendations. In brief, day 10 hPSC-derived HLF+ HOXA+ hematopoietic progenitors were collected, counted, and seeded in StemSpan Lymphoid Progenitor Expansion Medium at a density of 1-2x10^4^ cells/mL (1x10^4^ cells/mL for CD34+ cord blood HSPCs) in plates pre-coated with Lymphoid Differentiation Coating Material. On day 3 (referring to 3 days of T cell differentiation, after the HLF+ HOXA+ hematopoietic progenitor stage), cells were supplemented with an equal volume of StemSpan Lymphoid Progenitor Expansion Medium. On days 7 and 10, half medium changes were performed. On day 14, cells were harvested, counted, and reseeded in StemSpan T Cell Progenitor Maturation Medium at a density of 1x10^5^-1x10^6^ cells/mL in plates pre-coated with Lymphoid Differentiation Coating Material. On day 17, cells were supplemented with an equal volume of StemSpan T Cell Progenitor Maturation Medium. On days 21 and 24, half media changes were performed. On day 28, cells were collected, counted, and processed for flow cytometry.

For further maturation into CD8+ single-positive T cells, T cells from day 28 were harvested, counted, and reseeded in StemSpan T Cell Progenitor Maturation Medium supplemented with IL-15 (10 ng/mL [Peprotech, 200-15]) and 12.5μL/mL ImmunoCult Human CD3/CD28/CD2 T Cell Activator (STEMCELL Technologies, 10970) at a density of 1x10^6^ cells/mL in plates pre-coated with Lymphoid Differentiation Coating Material. On day 31, cells were supplemented with an equal volume of StemSpan T Cell Progenitor Maturation Medium supplemented with IL-15. On day 35, cells were collected, counted, and processed for flow cytometry.

#### Differentiation of hPSC-derived HLF+ HOXA+ hematopoietic progenitors into NK cells

NK cell differentiation was performed using co-culture with OP9-DLL4*-IRES-GFP* (alternatively known as “OP9-DLL4”) feeder cells.^149^ Day 10 hPSC-derived HLF+ HOXA+ hematopoietic progenitors were collected, counted and reseeded onto a confluent layer of OP9-DLL4*-IRES-GFP* feeder cells at a concentration of 1x10^4^-1x10^5^ cells/mL (1x10^3^ cells/mL for CD34+ cord blood HSPCs) in αMEM (Thermo Fisher, 12-571-063) supplemented with 20% FBS (R&D Systems, S11550), 1% penicillin/streptomycin (Thermo Fisher, 15-140-122), SCF (30 ng/mL [Peprotech, 300-07]), FLT3L (5 ng/mL [Peprotech, 300-19]), IL-7 (5 ng/mL [Peprotech, 200-07]), IL-15 (10 ng/mL [Peprotech, 200-15]), and for the first week of culture only, IL-3 (5 ng/mL [Peprotech, 200-03]). Cells were supplemented with an equal volume of media on days 3 and 10, and cells were collected and reseeded onto fresh OP9-DLL4*-IRES-GFP* feeders on day 7. On day 14, cells were collected, counted, and processed for flow cytometry.

#### NK cell killing assay

On day -1, 10^3^ OP9-DLL4*-IRES-GFP* feeder cells were seeded per well of a 96-well plate in αMEM (Thermo Fisher, 12-571-063) supplemented with 20% FBS (R&D Systems, S11550) and 1% penicillin/streptomycin (Thermo Fisher, 15-140-122). On day 0, NK cells derived from human CD34+ cord blood HSPCs or hPSC-derived HLF+ HOXA+ hematopoietic progenitors were collected, counted, and MACS enriched for CD56+ cells following the manufacturer’s instructions. In brief, cells were stained with anti-human CD56 APC antibody (Biolegend, 318310), and then they were stained with anti-APC microbeads (Miltenyi, 130-090-855) before performing magnetic separation.

After MACS enrichment, 10^4^ CD56+ NK cells were seeded onto OP9-DLL4*-IRES-GFP* feeders per well of a 96-well plate in αMEM + 20% FBS + 1% penicillin/streptomycin. Cells were cultured in an Incucyte Live-Cell Analysis System and imaged every hour for 72 hours. The total GFP+ area was measured to quantify NK cell killing efficacy over time.

#### Differentiation of hPSC-derived HLF+ HOXA+ hematopoietic progenitors into B cells

B cell differentiation was performed using co-culture with MS5 feeder cells (DSMZ). Day 10 hPSC-derived HLF+ HOXA+ hematopoietic progenitors were collected, counted, and reseeded onto a confluent layer of MS5 feeder cells at a concentration of 1-5x10^5^ cells/mL (1x10^3^-1x10^4^ cells/mL for CD34+ cord blood HSPCs) in αMEM (Thermo Fisher, 12-571-063) + 10% FBS (R&D Systems, S11550) + 1% penicillin/streptomycin (Thermo Fisher, 15-140-122) + SCF (100 ng/mL [Peprotech, 300-07]) + G-CSF (10 ng/mL [Peprotech, 300-23]). Cells were supplemented with equal volume of media on days 3, 10, and 17, and cells were collected and reseeded onto fresh MS5 feeders on days 7 and 14. On day 21, cells were collected, counted, and processed for flow cytometry.

#### Culture of human cord blood hematopoietic stem and progenitor cells

Human cord blood CD34+ hematopoietic stem and progenitor cells (HSPCs [StemExpress, CB3400.5C]) were thawed and cultured in StemSpan II medium (STEMCELL Technologies, 09655) supplemented with SCF (20 ng/mL [Peprotech, 300-07]), TPO (50 ng/mL [Peprotech, 300-18]), FLT3L (20 ng/mL [Peprotech, 300-19]), IL-6 (20 ng/mL [Peprotech, 200-06]), UM171 (75 nM [ApexBio, A8950]), and SR1 (750 nM [Cellagen Technology, C7710-5]). Cells were seeded at a concentration of 2x10^5^ cells/mL in U-bottom tissue culture treated plates. Each day, cells were gently triturated, and half of the cell suspension was transferred to a new well with all wells supplemented with fresh media to maintain the culture at low density. CD34+ cord blood HSPCs were either used immediately after thaw, or were cultured 3 days or less for all assays.

#### Dissociation, antibody staining, and flow cytometry analysis of cultured cells

Different methods were used to dissociate distinct types of cultured cells for flow cytometric analysis. First, undifferentiated and differentiated hPSCs were typically dissociated by incubation in TrypLE Express (Thermo Fisher, 12604013) for 3-5 minutes at 37 °C, followed by gentle trituration. Second, hPSC-derived hematopoietic progenitors were also more gently dissociated by incubation in Papain (0.5 mg/mL; Worthington Biochemical, LS003118) dissolved in PBS + 1.1 mM EDTA (Thermo Fisher, 15575020) + 0.067 mM 2-mercaptoethanol (Gibco, 21985023) + 5.5 mM Cysteine-HCl (Sigma, C7477) for 30-45 minutes at 37 °C, followed by gentle trituration, as described above. Third, hPSC-derived downstream blood and immune cells as well as cord blood HSPCs were less adherent and were therefore simply dissociated by gentle trituration.

After dissociation, cells were diluted 1:10 in DMEM/F12 and centrifuged at 500g for 5 minutes at 4 °C. Cell pellets were resuspended in FACS buffer (PBS + 1 mM EDTA [Thermo Fisher] + 2% v/v FBS [R&D Systems] + 1% penicillin/streptomycin [Thermo Fisher]). Generally speaking, fluorophore-conjugated primary antibodies were added to cells in FACS buffer and incubated for 30 minutes at 4 °C, protected from light. After staining, cells were washed twice with FACS buffer and resuspended in FACS buffer supplemented with either DAPI (1:10,000, Biolegend) or Propidium Iodide (PI; 1:1,000, Biolegend) for live/dead cell discrimination. Cells were passed through a 40 μM filter prior to flow cytometry. Flow cytometry was performed on a Beckman Coulter CytoFlex Analyzer, BD FACSymphony A5, or BD FACSAria II SORP. All data analysis was done in FlowJo. Cells were first gated based on forward and side scatter, followed by height and width for doublet discrimination. Subsequently, live cells were identified as those that did not stain for DAPI or propidium iodide.

Flow cytometry compensation was performed using compensation beads (BD Biosciences, 552845 and 552843).

#### Quantitative PCR

RNA extraction, reverse transcription, and quantitative PCR (qPCR) was generally performed as described previously.^103^ Cultured cells were directly lysed in the culture plate, or alternatively, were purified via FACS and then lysed, using 350 μL of RLT Plus Buffer. RNA was then extracted using the RNeasy Plus Micro Kit (Qiagen) according to the manufacturer’s protocol. 300 ng of total RNA was reverse transcribed into cDNA for qPCR using the High-Capacity cDNA Reverse Transcription Kit (Applied Biosystems) according to the manufacturer’s protocol. qPCR was performed in 384-well format as previously described,^103^ using gene-specific forward and reverse primers, as well as the SensiFAST SYBR Green Lo-ROX Kit (Thomas Scientific), on a QuantStudio 5 qPCR machine (Thermo Fisher). qPCR primer sequences are provided in Table S4. Expression of all genes was normalized to the levels of the reference gene *YWHAZ*.

#### Immunocytochemistry of hPSC-derived cell types

Adherent cells were washed with PBS, fixed in 4% paraformaldehyde for 10 minutes at room temperature, washed three times in PBS for 5 minutes each, and then permeabilized and blocked in blocking buffer (PBS + 0.1% Triton X-100 + 5% donkey serum) for 1 hour at 4 °C. Semi-adherent cells were harvested from the cell culture plate with TrypLE Express (Thermo Fisher, 12604013) and gentle trituration (as described above), then transferred to slides using a Cytospin cytocentrifuge (500g for 5 minutes), left to dry overnight, fixed in 2% paraformaldehyde for 10 minutes at room temperature, and then left to dry for 2 hours at room temperature.

Fixed cells were then incubated overnight in primary antibodies diluted in blocking buffer in a humidified chamber at 4 °C. The following day, cells were washed three times in PBS for 10 minutes each at room temperature and stained with Alexa Fluor-conjugated secondary antibodies diluted in blocking buffer for 1 hour at 4 °C. Cells were washed twice in PBS for 5 minutes each, and then stained with DAPI in PBS for an additional 5 minutes. Images were captured on an Olympus FV3000 confocal microscope. Images were rendered and analyzed with FIJI/ImageJ software.

#### Bulk-population RNA-sequencing of differentiated hPSCs and comparison with primary human hematopoietic stem and progenitor cells

For bulk population RNA-sequencing, we employed fluorescence activated cell sorting (FACS) to purify (1) CD144+ CD45+ hPSC-derived day 10 hematopoietic progenitors, (2) CD34+ CD38- CD90+ CD45RA- Lineage- cord blood HSCs, (3) CD34+ CD38- CD90- Lineage- cord blood multipotent progenitors (MPPs), and (4) CD34+ CD38+ CD90- Lineage- cord blood downstream hematopoietic progenitors (Figure S7C), following previously-published cell surface marker definitions of cord blood HSPCs.^207^^,^^208^ For FACS purification of cord blood HSPCs, we stained with the following antibodies — eFluor 450 Anti-human CD34 antibody (Thermo Fisher, 48-0349-42), FITC Anti-Human CD90 antibody (BD Biosciences, 555595), PE Anti-human CD45RA antibody (Biolegend, 304108), and APC Anti-Human CD38 antibody (BD Biosciences, 340439) — and also we employed a cocktail of PE Cy5-conjugated “lineage” antibodies to exclude differentiated blood and immune lineages, which recognized the following cell-surface markers: CD2, CD3, CD4, CD7, CD8, CD11b, CD14, CD16, CD19, CD20, CD56, and CD235A/GPA (BD Biosciences, 555328, 555341, 555348, 555362, 555368, 555389, 562335, 561725, 555414, 555624, 555517, 559944).

hPSC-derived cells or primary cord blood HSPCs were either directly lysed in the culture plate, or purified via FACS as indicated and then lysed, using 350 μL of RLT Plus Buffer and RNA was extracted using the RNeasy Plus Micro Kit (Qiagen) according to the manufacturer’s protocol. RNA concentration and quality was assessed by Agilent Bioanalyzer using the High Sensitivity RNA Analysis kit (Agilent, 5067-1513), to ensure that RNA was of high quality (RNA integrity number [RIN] > 7).

1 ng of total RNA was further processed to synthesize cDNA using the SMART-Seq2 pipeline.^209^ cDNA size distribution and concentration were assessed by Agilent Bioanalyzer using the High Sensitivity DNA Analysis kit (Agilent, 5067-4626). The Illumina Nextera XT DNA Sample Preparation kit (Illumina, FC-131-1096) was used for library construction and the KAPA Library Quantification Kit (Kapa Biosystems, KK4854) was used for library quantification. Library size distribution was assessed by Agilent Bioanalyzer. RNA-sequencing libraries were sequenced on the Illumina NovaSeq 6000 sequencer, to obtain 2x150bp paired-end reads (PE150).

Kallisto^153^ was used to align reads to the hg38 human reference genome (Homo_sapiens.GRCh38.96.gtf, downloaded from Ensemble) and to quantify transcript level abundances. Default parameters were used for quantification, and data were imported into DESeq2^151^ using tximport^155^ for all downstream analysis in R, including differential gene expression analysis.

Bulk population RNA-sequencing data of FACS-purified CD144+ CD45+ HSCs and CD144- CD45+ non-HSCs from the Carnegie Stage 15-16 dorsal aorta (CS15-16) were downloaded from a previous report^143^ (NCBI accession GSE151877). Raw RNA-seq reads from this previous study were aligned and analyzed as described above. Regarding the nomenclature surrounding this dataset: it was previously shown that all HSCs in the human dorsal aorta reside in the CD144+ CD45+ fraction,^156^ which we thus define as “HSCs”, in contrast to the CD144+ CD45- fraction, which we refer to as “non-HSCs”.

Computational scripts are available at Github: https://github.com/lohlaboratory/blood-differentiation.

#### Single-cell RNA-sequencing of differentiated hPSCs

Using the 10x Genomics Chromium v3 platform,^210^ we performed single-cell RNA-seq on day-0 undifferentiated hPSCs, day-1 posterior primitive streak, day-2 posterior primitive streak, day-3 lateral mesoderm, day-4 artery endothelium, day-7 hemogenic endothelium, and day-10 HLF+ HOXA+ hematopoietic progenitors, all of which were generated from the H7 hPSC line. Libraries were prepared using the Chromium 3’ Kit v3 (10x Genomics, 1000075) and Chromium Single Cell Chips (10x Genomics, 1000073) following the manufacturer’s guidelines. Libraries were prepared with i7 indices (compatible with Illumina sequencers) and then multiplexed. Multiplexed libraries were sequenced across two lanes of the Illumina NovoSeq 6000 sequencer to obtain 2x150bp paired-end reads (PE150). Unique sequences in each i7 index were used for demultiplexing.

Computational analysis of single-cell RNA-seq data was performed largely as described previously.^104^ Cell Ranger (10x Genomics) was used to perform read alignment to the hg38 reference genome, filtering, barcode counting, and unique molecular identifier (UMI) counting. Cell matrix files generated from Cell Ranger were imported into RStudio using the function ‘‘Read10x_h5” in Seurat v4^154^ to generate a Seurat object. Subsequent processing followed previously described workflows (https://satijalab.org/seurat/articles/pbmc3k_tutorial), which included normalizing data, finding variable features, scaling, and performing linear dimensional reduction to create Uniform Manifold Approximation and Projection (UMAP) graphs.^211^ Importantly, all cells within each sample population were shown on the UMAP graph, without any pre-selection of a cell-type of interest.

Computational scripts are available at Github: https://github.com/lohlaboratory/blood-differentiation.

#### Comparison of hPSC-derived hematopoietic progenitors generated using various differentiation protocols by single-cell RNA-sequencing

We downloaded published single-cell RNA-sequencing data of hPSC-derived hematopoietic progenitors differentiated using three different protocols that were originally developed by the Keller laboratory^54^ (European Bioinformatics Institute Accession E-MTAB-8205 sample EXP2_CTRL2_4823STDY7231845),^144^ the Zandstra laboratory (NCBI Accession GSE207157 sample GSM6280645),^145^ or the Elefanty, Stanley, and Ng laboratories (Calvanese et al.; NCBI Accession GSE162950 sample GSM6205033).^30^ Data were processed as described above in the section “Single-cell RNA-sequencing of differentiated hPSCs” using Seurat v4^154^ (https://satijalab.org/seurat/articles/pbmc3k_tutorial) to generate UMAP plots.^211^ All cells within each of the respective sample populations were shown on the UMAP projection, without any pre-selection of a cell-type of interest.

Computational scripts are available at Github: https://github.com/lohlaboratory/blood-differentiation.

#### Comparison of differentiated hPSCs and primary human hematopoietic stem and progenitor cells by single-cell RNA-sequencing

Single-cell RNA-sequencing data of human fetal hematopoietic stem and progenitor cells (HSPCs) were downloaded from a previous report^30^ (NCBI Accession GSE162950, samples GSM4968832 and GSM4968833). hPSC-derived (*in vitro*) and human fetal (*in vivo*) data were merged and integrated using Seurat v4^154^ in accordance with previously described methods (https://satijalab.org/seurat/articles/integration_introduction). In the resultant merged dataset, we annotated different cell-types based on expression of cell-type-specific markers.^30^

Computational scripts are available at Github: https://github.com/lohlaboratory/blood-differentiation.

#### Computational analysis of mouse embryo single-cell RNA-sequencing data

Single-cell RNA-sequencing data of E8.5-E11 whole mouse embryos were downloaded from a previous report (Qiu et al., 2023; NCBI Accession GSE186069 and GSE228590).^85^ We employed the cell-type annotations defined by the authors of the original study.^85^

Single-cell RNA-sequencing data of E10-E11 mouse hematopoietic progenitor and endothelial cells were downloaded from a previous report (NCBI Accession GSE112642).^28^ We employed the cell-type annotations defined by the authors of the original study.^28^

Single-cell RNA-sequencing data of whole E8.5 mouse embryos were plotted using an online browser (https://marionilab.cruk.cam.ac.uk/MouseGastrulation2018/).^81^ We employed the cell-type annotations defined by the authors of the original study.^81^

Computational scripts are available at Github: https://github.com/lohlaboratory/blood-differentiation.

#### Transplantation of human HSPCs or hPSC-derived hematopoietic progenitors into recipient mice

Male and female 8- to 12-week-old NOD-SCID Il2rg-/- (NSG) mice were used as recipient mice. Immediately prior to transplantation, NSG mice received a single dose of 2Gy irradiation. 1-3 million hPSC-derived hematopoietic progenitors, or alternatively, 80,000-100,000 human CD34+ cord blood hematopoietic stem and progenitor cells were transplanted intrafemorally in 20-30 μL IMDM + 20% FBS into irradiated NSG mice. 3-6 months post-transplantation, mice were sacrificed, and bone marrow was analyzed by flow cytometry to detect the presence of human cells. The following antibodies were used to detect human hematopoietic cells in transplanted mice via flow cytometry: BD Horizon V450 anti-human CD45 antibody (BD Biosciences, 560367), APC-Cy7 anti-Human HLA-A/B/C antibody (Biolegend, 311425), PE anti-Human CD33 antibody (Biolegend, 303404), and APC anti-Human CD19 antibody (Biolegend, 302212).

#### Intravital bioluminescent imaging

Twenty minutes prior to imaging, mice were injected intraperitoneally with 100 μL of 15 mM AkaLumine HCl (otherwise known as TokeOni [Aobious], which was dissolved in H_2_O).^168^ Mice were anesthetized using isoflurane and placed in the imaging chamber of a SII Lago-X bioluminescent imaging machine. Imaging parameters were kept constant throughout the duration of each experiment with no images reaching saturation (Binning = 4, FStop = 1.2, exposure time = 10 s). Subsequent image analysis was done in Aura.

#### Construction of *Efnb2-CreERT2* mice

Pronuclear injection was performed on 1-cell stage mouse zygotes from the C56BL/6 genetic background, to deliver a mixture of sgRNA (5 ng/μL), transgenic donor DNA (5 ng/μL) and Cas9 protein (15 ng/μL). Subsequently, microinjected zygotes were transferred into the oviduct of a pseudopregnant CD1 surrogate mother.

The sgRNA sequence targeting the endogenous mouse *Efnb2* gene was as follows: TCAGACCTTGTAGTAAATGT. The transgenic donor DNA sequence encoding the *GSG-P2A-CreERT2-F5* cassette, including 5’ and 3’ homology arms targeting the *Efnb2* locus, was as follows:

GCACTTTTCGGGGAAATGTGCGCGGAACCCCTATTTGTTTATTTTTCTAAATACATTCAAATATGTATCCGCTCATGAGACAATAACCCTGATAAATGCTTCAATAATATTGAAAAAGGAAGAGTATGAGTATTCAACATTTCCGTGTCGCCCTTATTCCCTTTTTTGCGGCATTTTGCCTTCCTGTTTTTGCTCACCCAGAAACGCTGGTGAAAGTAAAAGATGCTGAAGATCAGTTGGGTGCACGAGTGGGTTACATCGAACTGGATCTCAACAGCGGTAAGATCCTTGAGAGTTTTCGCCCCGAAGAACGTTTTCCAATGATGAGCACTTTTAAAGTTCTGCTATGTGGCGCGGTATTATCCCGTATTGACGCCGGGCAAGAGCAACTCGGTCGCCGCATACACTATTCTCAGAATGACTTGGTTGAGTACTCACCAGTCACAGAAAAGCATCTTACGGATGGCATGACAGTAAGAGAATTATGCAGTGCTGCCATAACCATGAGTGATAACACTGCGGCCAACTTACTTCTGACAACGATCGGAGGACCGAAGGAGCTAACCGCTTTTTTGCACAACATGGGGGATCATGTAACTCGCCTTGATCGTTGGGAACCGGAGCTGAATGAAGCCATACCAAACGACGAGCGTGACACCACGATGCCTGTAGCAATGGCAACAACGTTGCGCAAACTATTAACTGGCGAACTACTTACTCTAGCTTCCCGGCAACAATTAATAGACTGGATGGAGGCGGATAAAGTTGCAGGACCACTTCTGCGCTCGGCCCTTCCGGCTGGCTGGTTTATTGCTGATAAATCTGGAGCCGGTGAGCGTGGGTCTCGCGGTATCATTGCAGCACTGGGGCCAGATGGTAAGCCCTCCCGTATCGTAGTTATCTACACGACGGGGAGTCAGGCAACTATGGATGAACGAAATAGACAGATCGCTGAGATAGGTGCCTCACTGATTAAGCATTGGTAACTGTCAGACCAAGTTTACTCATATATACTTTAGATTGATTTAAAACTTCATTTTTAATTTAAAAGGATCTAGGTGAAGATCCTTTTTGATAATCTCATGACCAAAATCCCTTAACGTGAGTTTTCGTTCCACTGAGCGTCAGACCCCGTAGAAAAGATCAAAGGATCTTCTTGAGATCCTTTTTTTCTGCGCGTAATCTGCTGCTTGCAAACAAAAAAACCACCGCTACCAGCGGTGGTTTGTTTGCCGGATCAAGAGCTACCAACTCTTTTTCCGAAGGTAACTGGCTTCAGCAGAGCGCAGATACCAAATACTGTCCTTCTAGTGTAGCCGTAGTTAGGCCACCACTTCAAGAACTCTGTAGCACCGCCTACATACCTCGCTCTGCTAATCCTGTTACCAGTGGCTGCTGCCAGTGGCGATAAGTCGTGTCTTACCGGGTTGGACTCAAGACGATAGTTACCGGATAAGGCGCAGCGGTCGGGCTGAACGGGGGGTTCGTGCACACAGCCCAGCTTGGAGCGAACGACCTACACCGAACTGAGATACCTACAGCGTGAGCTATGAGAAAGCGCCACGCTTCCCGAAGGGAGAAAGGCGGACAGGTATCCGGTAAGCGGCAGGGTCGGAACAGGAGAGCGCACGAGGGAGCTTCCAGGGGGAAACGCCTGGTATCTTTATAGTCCTGTCGGGTTTCGCCACCTCTGACTTGAGCGTCGATTTTTGTGATGCTCGTCAGGGGGGCGGAGCCTATGGAAAAACGCCAGCAACGCGGCCTTTTTACGGTTCCTGGCCTTTTGCTGGCCTTTTGCTCACATGTTCTTTCCTGCGTTATCCCCTGATTCTGTGGATAACCGTATTACCGCCTTTGAGTGAGCTGATACCGCTCGCCGCAGCCGAACGACCGAGCGCAGCGAGTCAGTGAGCGAGGAAGCGGAAGAGCGCCCAATACGCAAACCGCCTCTCCCCGCGCGTTGGCCGATTCATTAATGCAGCTGGCACGACAGGTTTCCCGACTGGAAAGCGGGCAGTGAGCGCAACGCAATTAATGTGAGTTAGCTCACTCATTAGGCACCCCAGGCTTTACACTTTATGCTTCCGGCTCGTATGTTGTGTGGAATTGTGAGCGGATAACAATTTCACACAGGAAACAGCTATGACCATGATTACGCCAAGCTCGAAATTAACCCTCACTAAAGGGAACAAAAGCTGGAGCTGATTTAAATTTCCCGGGTCCCATCACTCCTTCCTTTCTGGCGCATCGCTGCTGGTAGGCAGGGCTTCTGTGGAAGGCGGGCGTTTAAAGACGGACATATAACACCCAGTTTCCAGCATGAGGGAAGAGCAGGCCAGGCCAGGGCTGCACCATGCTCTGGAGTAGCTGCGTGTGCCTTTTTTCCAGCCGAGCCTGGTGCATCACCGGGAAGTGCAGAGGCTAGGACGGCCCAGGGCTTGCTGGCTGTCTCGCCATTCTGCTGCCGGTGACACAGACAAGGAAGCAGTTGGGGATTGTATGAGCATTGGACCTTGAGGCTCCTTTGCCCTGTCTGTCAAGTTCGCTCTGAGGGTTGAGCGTTGGACATGCTTAATTATTACATTTCGGGGTTTCTGCAAGAAACAGGGCACACCCAGGACTCTGGTAACTGGCTCTTCTTGCTCCTGTGCAGGTTCTAGCACCGATGGCAACAGCGCGGGGCATTCCGGGAACAATCTCCTGGGTTCCGAAGTGGCCTTATTCGCAGGGATCGCATCAGGATGCATCATCTTCATCGTCATCATCATCACTTTGGTGGTGCTGCTGCTCAAGTACCGCAGGAGACACCGCAAACACTCTCCACAGCACACGACCACGCTGTCTCTCAGCACACTGGCCACGCCCAAGCGAGGTGGCAACAACAATGGCTCGGAGCCCAGTGACGTTATCATACCACTAAGGACTGCAGACAGCGTCTTCTGCCCGCACTACGAGAAGGTCAGCGGGGACTATGGGCACCCGGTGTACATCGTGCAGGAGATGCCCCCACAGAGTCCTGCAAACATTTACTACAAGGTCGGATCCGGAGCTACTAACTTCAGCCTGCTGAAGCAGGCTGGAGACGTGGAGGAGAACCCTGGACCTATGTCCAATTTACTGACCGTACACCAAAATTTGCCTGCATTACCGGTCGATGCAACGAGTGATGAGGTTCGCAAGAACCTGATGGACATGTTCAGGGATCGCCAGGCGTTTTCTGAGCATACCTGGAAAATGCTTCTGTCCGTTTGCCGGTCGTGGGCGGCATGGTGCAAGTTGAATAACCGGAAATGGTTTCCCGCAGAACCTGAAGATGTTCGCGATTATCTTCTATATCTTCAGGCGCGCGGTCTGGCAGTAAAAACTATCCAGCAACATTTGGGCCAGCTAAACATGCTTCATCGTCGGTCCGGGCTGCCACGACCAAGTGACAGCAATGCTGTTTCACTGGTTATGCGGCGGATCCGAAAAGAAAACGTTGATGCCGGTGAACGTGCAAAACAGGCTCTAGCGTTCGAACGCACTGATTTCGACCAGGTTCGTTCACTCATGGAAAATAGCGATCGCTGCCAGGATATACGTAATCTGGCATTTCTGGGGATTGCTTATAACACCCTGTTACGTATAGCCGAAATTGCCAGGATCAGGGTTAAAGATATCTCACGTACTGACGGTGGGAGAATGTTAATCCATATTGGCAGAACGAAAACGCTGGTTAGCACCGCAGGTGTAGAGAAGGCACTTAGCCTGGGGGTAACTAAACTGGTCGAGCGATGGATTTCCGTCTCTGGTGTAGCTGATGATCCGAATAACTACCTGTTTTGCCGGGTCAGAAAAAATGGTGTTGCCGCGCCATCTGCCACCAGCCAGCTATCAACTCGCGCCCTGGAAGGGATTTTTGAAGCAACTCATCGATTGATTTACGGCGCTAAGGATGACTCTGGTCAGAGATACCTGGCCTGGTCTGGACACAGTGCCCGTGTCGGAGCCGCGCGAGATATGGCCCGCGCTGGAGTTTCAATACCGGAGATCATGCAAGCTGGTGGCTGGACCAATGTAAATATTGTCATGAACTATATCCGTAACCTGGATAGTGAAACAGGGGCAATGGTGCGCCTGCTGGAAGATGGCGATCTCGAGCCATCTGCTGGAGACATGAGAGCTGCCAACCTTTGGCCAAGCCCGCTCATGATCAAACGCTCTAAGAAGAACAGCCTGGCCTTGTCCCTGACGGCCGACCAGATGGTCAGTGCCTTGTTGGATGCTGAGCCCCCCATACTCTATTCCGAGTATGATCCTACCAGACCCTTCAGTGAAGCTTCGATGATGGGCTTACTGACCAACCTGGCAGACAGGGAGCTGGTTCACATGATCAACTGGGCGAAGAGGGTGCCAGGCTTTGTGGATTTGACCCTCCATGATCAGGTCCACCTTCTAGAATGTGCCTGGCTAGAGATCCTGATGATTGGTCTCGTCTGGCGCTCCATGGAGCACCCAGTGAAGCTACTGTTTGCTCCTAACTTGCTCTTGGACAGGAACCAGGGAAAATGTGTAGAGGGCATGGTGGAGATCTTCGACATGCTGCTGGCTACATCATCTCGGTTCCGCATGATGAATCTGCAGGGAGAGGAGTTTGTGTGCCTCAAATCTATTATTTTGCTTAATTCTGGAGTGTACACATTTCTGTCCAGCACCCTGAAGTCTCTGGAAGAGAAGGACCATATCCACCGAGTCCTGGACAAGATCACAGACACTTTGATCCACCTGATGGCCAAGGCAGGCCTGACCCTGCAGCAGCAGCACCAGCGGCTGGCCCAGCTCCTCCTCATCCTCTCCCACATCAGGCACATGAGTAACAAAGGCATGGAGCATCTGTACAGCATGAAGTGCAAGAACGTGGTGCCCCTCTATGACCTGCTGCTGGAGGCGGCGGACGCCCACCGCCTACATGCGCCCACTAGCCGTGGAGGGGCATCCGTGGAGGAGACGGACCAAAGCCACTTGGCCACTGCGGGCTCTACTTCATCGCATTCCTTGCAAAAGTATTACATCACGGGGGAGGCAGAGGGTTTCCCTGCCACAGCTTGACGTCGACGAAGTTCCTATTCCGAAGTTCCTATTCTTCAAAAGGTATAGGAACTTCGCTAGGCCGCAGACCTGCGACTCCCAAGGGAACTCGCACCTTGTTCTTGGGCACGCAGGGACTGCCTGAGCCTGGCTGTGGGGGCAGGATGCCTCCTGGAAGAGCCTGGATCTGGACAGTTTTGTAGTCTGTAGCTTTTCCGACCCTGGGGACCACAGACCCTCCCCGGAAGCTGGAAGACTGCTAGGAGATCCCCACTTGGACTGCCGCGGCCCCACGCGGACCTCCAAGCCATGCACCCAGCCACTCAGGCCTCTGCAGAGCCCGGGGAGGACACGGTAGGCTATGGATGGCGCAGCAGCATCTTAGGAGAAGGTTGCGCACCAGGCCGGCCCCTGCCTCCACGTTTCCTGCCGTGCACACTGGACTTATCACTTGGACCTCGGGTTCAGTAAGGTTTTCAAAGATCTCTAGTGTTTAGTCCTCACTCACTCACTCACTCACTCACTCCTTCTCTTGCCAGGGCTCTGCAGCAAACTCCCTAGACCCCTCACTCCACGTACTGCATCATTACGGGACACTCACCACAGAGTCCCAGCTCCACCCTTTACACCAAGATCAAATTAGATGGGTATTAGGTACAGAAGAACCCTGCTTGCCTGGAGGCCGGGTCAGCCGGGAAGCGCAGATGTGTGGAGGAGTGAGGAGGTGCTGGCTGCCACGGCAGGTCAAGGCTGCTTGCTGCCCCTGGAGCATAGTAGGGATGCAGGAAGGAAATAGATAATGCTTTGGTTTTTCTGAGAGGACAGAGACAGGTGGGAGGTGACTGACTGGTGAGTGGTGGGGAGCCTTTCACTACCACACAGCTATGCAGCAGGGAATCAAAAGGCGCGCCTGTACCCAATTCGCCCTATAGTGAGTCGTATTACAATTCACTGGCCGTCGTTTTACAACGTCGTGACTGGGAAAACCCTGGCGTTACCCAACTTAATCGCCTTGCAGCACATCCCCCTTTCGCCAGCTGGCGTAATAGCGAAGAGGCCCGCACCGATCGCCCTTCCCAACAGTTGCGCAGCCTGAATGGCGAATGGGACGCGCCCTGTAGCGGCGCATTAAGCGCGGCGGGTGTGGTGGTTACGCGCAGCGTGACCGCTACACTTGCCAGCGCCCTAGCGCCCGCTCCTTTCGCTTTCTTCCCTTCCTTTCTCGCCACGTTCGCCGGCTTTCCCCGTCAAGCTCTAAATCGGGGGCTCCCTTTAGGGTTCCGATTTAGTGCTTTACGGCACCTCGACCCCAAAAAACTTGATTAGGGTGATGGTTCACGTAGTGGGCCATCGCCCTGATAGACGGTTTTTCGCCCTTTGACGTTGGAGTCCACGTTCTTTAATAGTGGACTCTTGTTCCAAACTGGAACAACACTCAACCCTATCTCGGTCTATTCTTTTGATTTATAAGGGATTTTGCCGATTTCGGCCTATTGGTTAAAAAATGAGCTGATTTAACAAAAATTTAACGCGAATTTTAACAAAATATTAACGCTTACAATTTAGGTG

### Quantification and statistical analysis

#### Statistical tests

One factor ANOVA (analysis of variance, alpha = 0.05) was performed to test for statistical significance in Figures 1K–1M. In all other figures, statistical significance was evaluated using a t test (alpha = 0.05). In all figures, the following notation was used to denote statistical significance: ^∗^p < 0.05, ^∗∗^p < 0.01, n.s. = not significant. Histograms and line graphs typically depict the mean, and error bars indicate standard error of the mean (SEM).

## Supplemental information

Document S1. Figures S1–S7, Table S4, and supplemental references

Table S1. Lineage tracing data, related to Figures 1 and S2 Raw data from *Cx40*-*CreERT2*; *Ai6* mouse, *Efnb2-CreERT2*; *Ai6* mouse, and *Apj-CreERT2*; *Ai6* mouse lineage tracing experiments, tabulating what timepoint 4OHT was administered, when cells were analyzed, and what percentage of cells were labeled (i.e., ZsGreen+). For fetal liver HSC analyses, each sample represents the average of an independent litter of mouse embryos. For adult analyses, each sample represents a single adult.

Table S2. Genes differentially expressed between hPSC-derived day 10 HLF+ HOXA+ hematopoietic progenitors and AGM HSCs, related to Figures 6 and S7 RNA-seq analysis of genes differentially expressed between hPSC-derived day 10 HLF+ HOXA+ hematopoietic progenitors and FACS-purified human fetal CD144+ CD45+ ventral AGM HSCs (ref. ^143^).

Table S3. Genes differentially expressed between hPSC-derived day 10 HLF+ HOXA+ hematopoietic progenitors and cord blood HSCs, related to Figure S7 RNA-seq analysis of genes differentially expressed between hPSC-derived day 10 HLF+ HOXA+ hematopoietic progenitors and FACS-purified CD34+ CD38- CD90+ CD45RA- Lineage- cord blood HSCs.

Document S2. Article plus supplemental information
