# Supplementary material for: Lineage-tracing hematopoietic stem cell origins in vivo to efficiently make human HLF+ HOXA+ hematopoietic progenitors from pluripotent stem cells
Source: Dev Cell. 2024 May 6;59(9):1110–1131.e22. doi: 10.1016/j.devcel.2024.03.003 (PMC11072092; doi:10.1016/j.devcel.2024.03.003)
Supplement: Document S1. Figures S1–S7, Table S4, and supplemental references [file mmc1.pdf]

Developmental Cell, Volume *59*

Supplemental information

Lineage-tracing hematopoietic stem cell origins *in vivo* to efficiently make human HLF+ HOXA+ hematopoietic progenitors from pluripotent stem cells

Jonas L. Fowler, Sherry Li Zheng, Alana Nguyen, Angela Chen, Xiaochen Xiong, Timothy Chai, Julie Y. Chen, Daiki Karigane, Allison M. Banuelos, Kouta Niizuma, Kensuke Kayamori, Toshinobu Nishimura, M. Kyle Cromer, David Gonzalez-Perez, Charlotte Mason, Daniel Dan Liu, Leyla Yilmaz, Lucile Miquerol, Matthew H. Porteus, Vincent C. Luca, Ravindra Majeti, Hiromitsu Nakauchi, Kristy Red-Horse, Irving L. Weissman, Lay Teng Ang, and Kyle M. Loh

Supplemental Figure 1

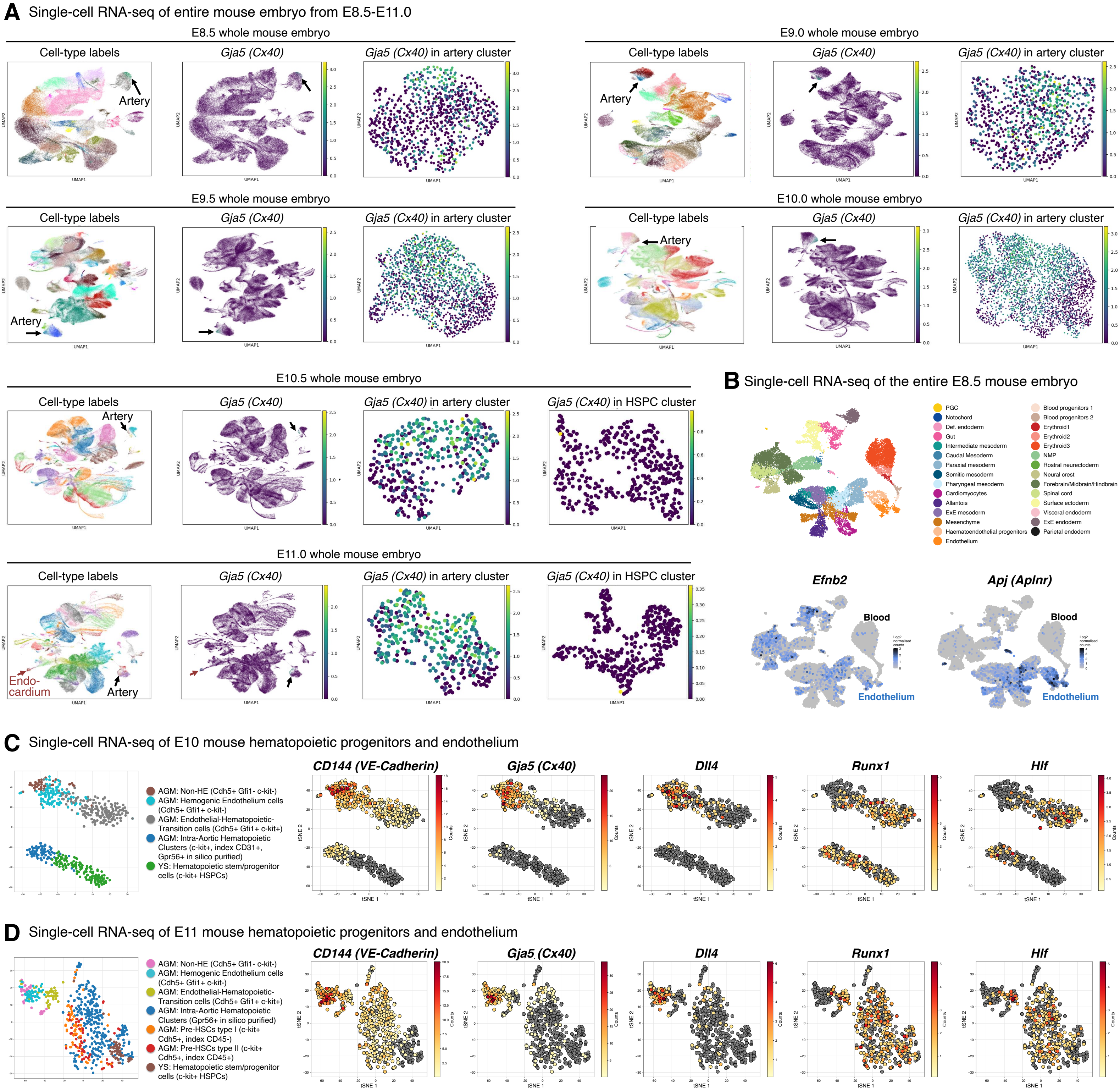

Supplemental Figure 1: Cell-type specificity of markers used for *in vivo* genetic lineage tracing, related to Figure 1.

A) scRNA-seq of the entire mouse embryo at 12-hour intervals between E8.5 and E11.0; data taken from a published resource[1]. Expression of *Cx40* (*Gja5*) in artery EC and hematopoietic stem and progenitor cell (HSPC) clusters; these clusters were defined by the original authors[1].
B) scRNA-seq of the entire E8.5 mouse embryo; data taken from a published resource[2]. Neither *Efnb2* nor *Aplnr*/*Apj* is expressed in hematopoietic progenitors at this stage. Cluster names were defined by the original authors[2].
C) scRNA-seq of endothelial and hematopoietic cells within the E10 mouse embryo aorta-gonad-mesonephros (AGM) region; data taken from a published resource[3]. Nascent *Hlf*+ HSPCs still express *VE-Cadherin* (*CD144/Cdh5*) to some extent, but minimally express *Cx40*.
D) scRNA-seq of endothelial and hematopoietic cells within the E11 mouse embryo AGM region; data taken from a published resource[3]. Nascent *Hlf*+ HSPCs still express *VE-Cadherin* (*CD144/Cdh5*) to some extent, but minimally express *Cx40*.

Supplemental Figure 2

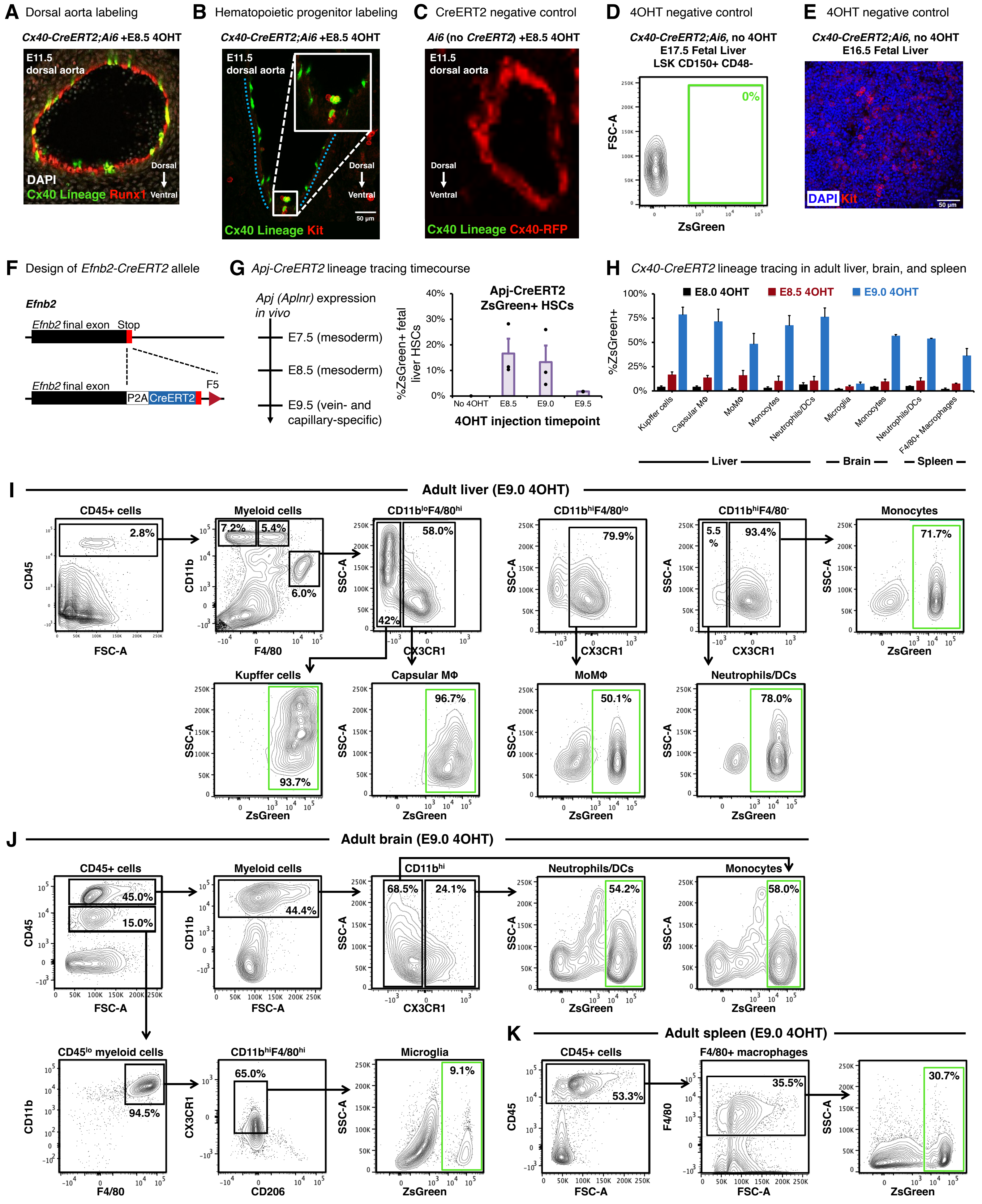

Supplemental Figure 2: Putative contribution of artery ECs to tissue-resident macrophages *in vivo* and control experiments for genetic lineage tracing, related to Figure 1.

A) 4OHT was administered to *Cx40-CreERT2*; *Ai6* (*ZsGreen* reporter) embryos at E8.5, resulting in ZsGreen labeling of Runx1+ hemogenic ECs in the E11.5 dorsal aorta, as shown by immunostaining.

B) 4OHT was administered to *Cx40-CreERT2*; *Ai6* (*ZsGreen* reporter) embryos at E8.5, resulting in ZsGreen labeling of Kit+ hematopoietic progenitors and Kit- ECs in the E11.5 dorsal aorta, as shown by immunostaining.

C) Heterozygous *Cx40-CreERT2/+* mice were crossed with homozygous *Ai6* (*ZsGreen* reporter) mice and 4OHT was administered at E8.5. Immunostaining was performed on a E11.5 CreERT2 negative embryo, showing that in the absence of CreERT2, 4OHT does not induce ZsGreen labeling, as shown by immunostaining. The *Cx40-CreERT2* allele also encodes *RFP* [4], which was used to visualize Cx40+ cells.

D) In the absence of 4OHT, there was no detectable ZsGreen labeling of Lineage- Sca1+ Kit+ CD150+ CD48- HSCs in the E17.5 fetal liver of *Cx40-CreERT2*; *Ai6* (*ZsGreen* reporter) embryos, as shown by flow cytometry.

E) In the absence of 4OHT, there was no detectable ZsGreen labeling of Kit+ hematopoietic progenitors in the E16.5 fetal liver of *Cx40-CreERT2*; *Ai6* (*ZsGreen* reporter) embryos, as shown by immunostaining.

F) Design of *Efnb2-CreERT2* knock-in mouse allele.

G) Vein and capillary ECs were lineage-traced in *Apj-CreERT2*; *Ai6* (*ZsGreen* reporter) embryos by administering 4OHT at E8.5, E9.0, or E9.5, and flow cytometry was performed to quantify the percentage of vein- or capillary-derived (i.e., ZsGreen+) CD150+ CD48- Lineage- Sca1+ Kit+ HSCs in the E14.5-E18.5 fetal liver. Each dot on the bar chart represents an independent litter. For each timepoint, at least 8 independent embryos from at least 3 independent litters were analyzed. Raw lineage tracing data are tabulated in **Table S1**.

H) Arteries were lineage-traced in *Cx40-CreERT2*; *Ai6* (*ZsGreen* reporter) embryos by administering 4OHT at E8.0, E8.5, or E9.0, and flow cytometry was performed to quantify the percentage of artery-derived (i.e., ZsGreen+) immune cells in the liver, brain, and spleen of 22-month-old adults. Raw lineage tracing data are tabulated in **Table S1**.

I) Arteries were lineage-traced in *Cx40-CreERT2*; *Ai6* (*ZsGreen* reporter) embryos by administering 4OHT at E9.0, and flow cytometry was performed to quantify the percentage of artery-derived (i.e., ZsGreen+) immune cells in the liver of 22-month-old adults. This revealed putative labeling of Kupffer cells, which are tissue-resident macrophages known to derive independently of HSCs.

J) Arteries were lineage-traced in *Cx40-CreERT2*; *Ai6* (*ZsGreen* reporter) embryos by administering 4OHT at E9.0, and flow cytometry was performed to quantify the percentage of artery-derived (i.e., ZsGreen+) immune cells in the brain of 22-month-old adults. This revealed putative labeling of microglia, which are tissue-resident macrophages known to derive independently of HSCs.

K) Arteries were lineage-traced in *Cx40-CreERT2*; *Ai6* (*ZsGreen* reporter) embryos by administering 4OHT at E9.0, and flow cytometry was performed to quantify the percentage of artery-derived (i.e., ZsGreen+) immune cells in the spleen of 22-month-old adults. This revealed putative labeling of splenic macrophages, some of which are known to arise independently of HSCs, but which are gradually replaced by HSC-derived monocytes throughout life[5].

Histograms depict the mean±SEM. Scale: 50 µm.

Supplemental Figure 3

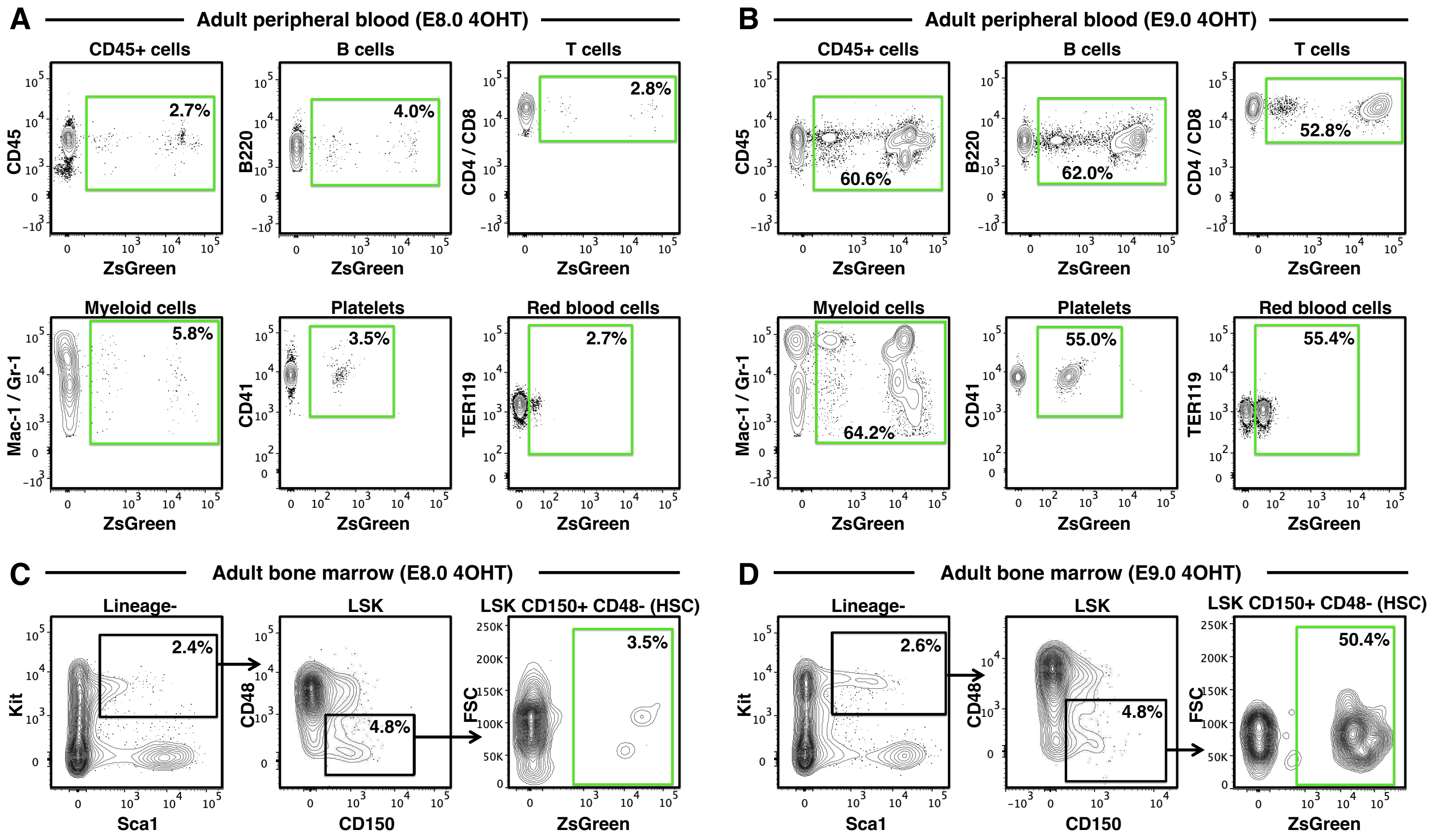

Supplemental Figure 3: Lineage tracing of artery-derived HSCs *in vivo*, related to Figure 2.

A) Arteries were lineage-traced in *Cx40-CreERT2*; *Ai6* (*ZsGreen* reporter) embryos by administering 4OHT at E8.0, and flow cytometry was performed to quantify the percentage of artery-derived (i.e., ZsGreen+) myeloid, erythroid, and lymphoid cells in the peripheral blood of 3-month-old adults.

B) Arteries were lineage-traced in *Cx40-CreERT2*; *Ai6* (*ZsGreen* reporter) embryos by administering 4OHT at E9.0, and flow cytometry was performed to quantify the percentage of artery-derived (i.e., ZsGreen+) myeloid, erythroid, and lymphoid cells in the peripheral blood of 3-month-old adults.

C) Arteries were lineage-traced in *Cx40-CreERT2*; *Ai6* (*ZsGreen* reporter) embryos by administering 4OHT at E8.0, and flow cytometry was performed to quantify the percentage of artery-derived (i.e., ZsGreen+) CD150+ CD48- Lineage- Sca1+ Kit+ HSCs in the bone marrow of 6-month-old adults. LSK: Lineage- Sca1+ Kit+.

D) Arteries were lineage-traced in *Cx40-CreERT2*; *Ai6* (*ZsGreen* reporter) embryos by administering 4OHT at E9.0, and flow cytometry was performed to quantify the percentage of artery-derived (i.e., ZsGreen+) CD150+ CD48- Lineage- Sca1+ Kit+ HSCs in the bone marrow of 6-month-old adults.

Supplemental Figure 4

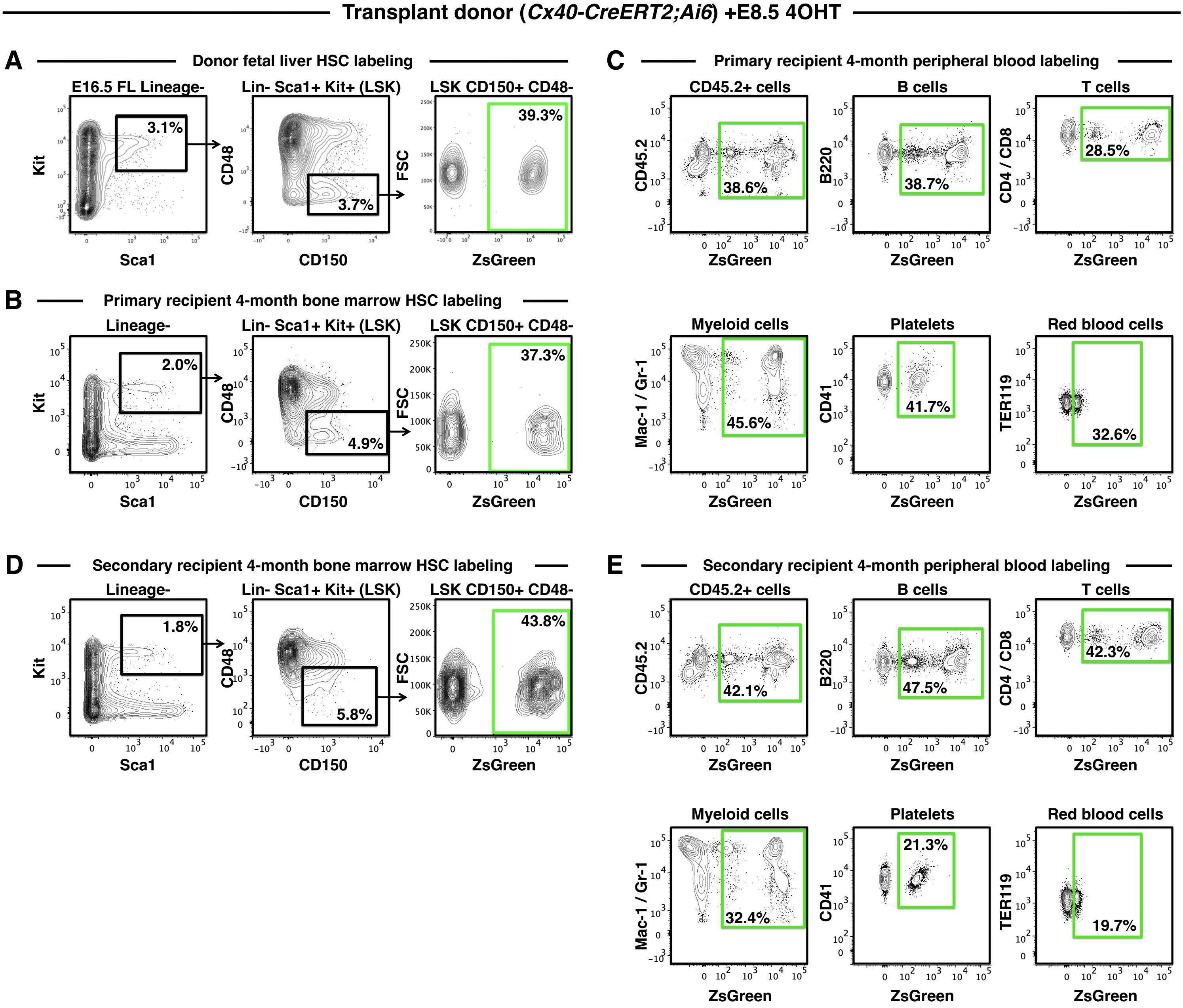

Supplemental Figure 4: Artery-derived HSCs are functional *in vivo* upon primary and secondary transplantation, related to Figure 3.

A) Arteries were lineage-traced in *Cx40-CreERT2*; *Ai6* (*ZsGreen* reporter) embryos by administering 4OHT at E8.5, and flow cytometry was performed to quantify the percentage of artery-derived (i.e., ZsGreen+) fetal liver HSCs at E16.5.

B) Flow cytometry of CD150+ CD48- Lineage- Sca1+ Kit+ HSCs in the bone marrow of primary recipient mice, four months after transplantation with E16.5 fetal liver cells that were originally obtained from lineage-traced *Cx40-CreERT2*; *Ai6* (*ZsGreen* reporter) embryos (4OHT administered at E8.5).

C) Flow cytometry of myeloid, erythroid, and lymphoid cells in the peripheral blood of primary recipient mice, four months after transplantation with E16.5 fetal liver cells that were originally obtained from lineage-traced *Cx40-CreERT2*; *Ai6* (*ZsGreen* reporter) embryos (4OHT administered at E8.5).

D) Flow cytometry of CD150+ CD48- Lineage- Sca1+ Kit+ HSCs in the bone marrow of secondary recipient mice that had been transplanted four months ago with bone marrow from primary recipient mice that were described in **Fig. 3C-D**.

E) Flow cytometry of myeloid, erythroid, and lymphoid cells in the peripheral blood of secondary recipient mice that had been transplanted four months ago with bone marrow from primary recipient mice that were described in **Fig. 3C-D**.

Supplemental Figure 5

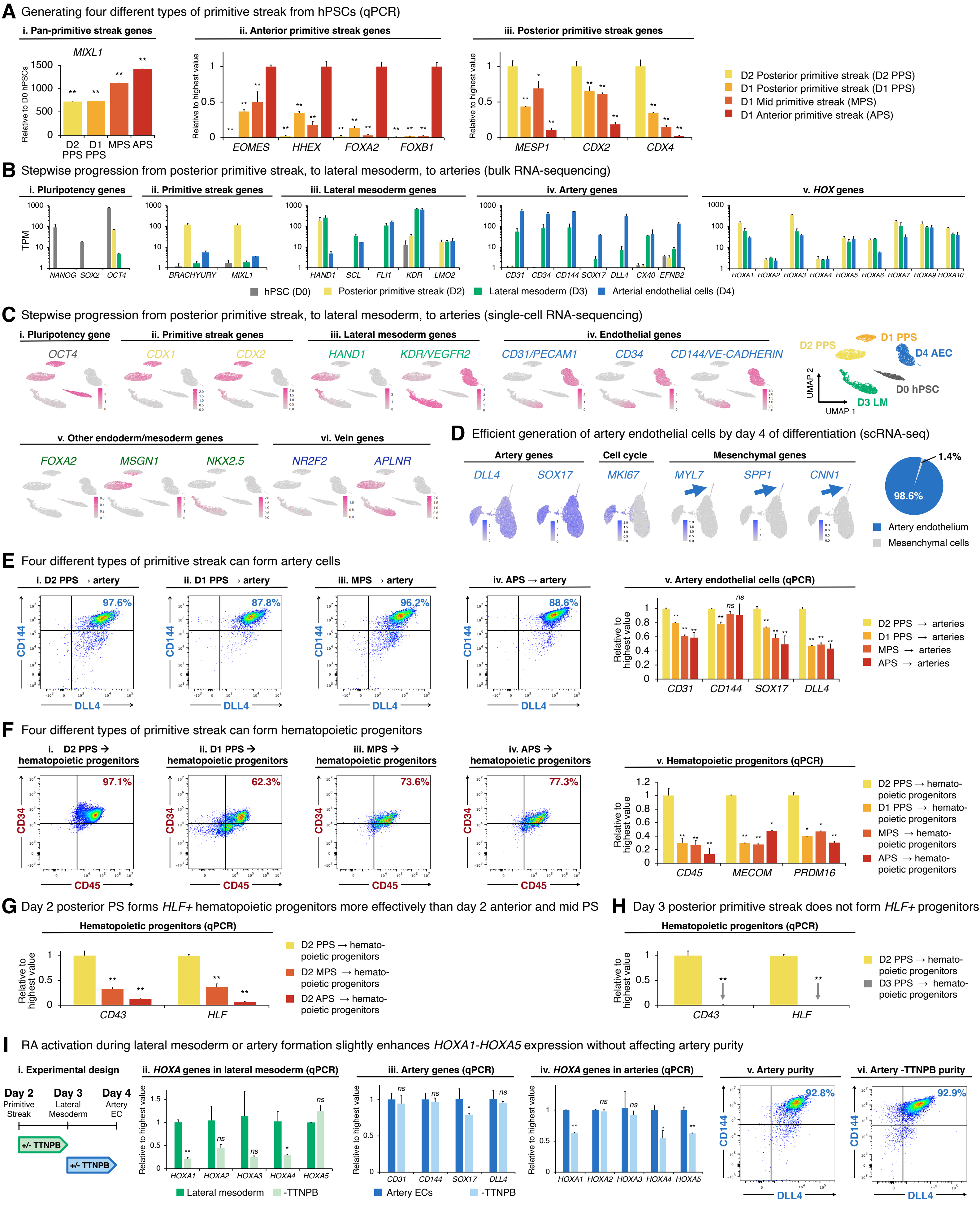

Supplementary Figure 5: Stepwise differentiation of hPSCs into posterior primitive streak, lateral mesoderm, and artery ECs, related to Figure 4.

A) qPCR of hPSCs differentiated into anterior primitive streak (day 1, “APS”), mid primitive streak (day 1, “MPS”), posterior primitive streak (day 1, “D1 PPS”), or posterior primitive streak (day 2, “D2 PPS”). (i) Expression normalized to undifferentiated hPSCs. (ii,iii) qPCR data were normalized to the sample with the highest expression in this experiment.

B) Bulk population RNA-seq of undifferentiated hPSCs (day 0), posterior primitive streak (day 2), lateral mesoderm (day 3) and artery ECs (day 4). Cells were colored by the day of differentiation they were profiled. All gene expression quantified in TPM (transcripts per million) units.

C) scRNA-seq of undifferentiated hPSCs (day 0), posterior primitive streak (day 2), lateral mesoderm (day 3) and artery ECs (day 4). Cells were colored by the day of differentiation they were profiled.

D) scRNA-seq of hPSC-derived artery ECs (day 4), showing gene expression (*left*) and cell-type assignment, defined by clustering (*right*).

E) hPSCs were initially differentiated into anterior primitive streak (day 1, “APS”), mid primitive streak (day 1, “MPS”), posterior primitive streak (day 1, “D1 PPS”), or posterior primitive streak (day 2, “D2 PPS”), and then further differentiated into artery endothelial cells, which were profiled by flow cytometry (i-iv) and qPCR (v). qPCR data were normalized to the sample with the highest expression in this experiment.

F) hPSCs were initially differentiated into anterior primitive streak (day 1, “APS”), mid primitive streak (day 1, “MPS”), posterior primitive streak (day 1, “D1 PPS”), or posterior primitive streak (day 2, “D2 PPS”), and then further differentiated into hematopoietic progenitors, which were profiled by flow cytometry (i-iv) and qPCR (v). qPCR data were normalized to the sample with the highest expression in this experiment.

G) hPSCs were initially differentiated into day 2 anterior primitive streak (“APS”), day 2 mid primitive streak (“MPS”), day 2 posterior primitive streak (“PPS”), and then were further differentiated into hematopoietic progenitors, which were profiled by qPCR. qPCR data were normalized to the sample with the highest expression in this experiment.

H) hPSCs were initially differentiated into day 2 posterior primitive streak (“D2 PPS”) or day 3 posterior primitive streak (“D3 PPS”), and then were further differentiated into hematopoietic progenitors, which were profiled by qPCR. qPCR data were normalized to the sample with the highest expression in this experiment.

I) hPSCs were differentiated into posterior primitive streak (day 2), which was subsequently differentiated into lateral mesoderm (day 3) in the presence or absence of retinoid pathway agonist TTNPB, followed by qPCR of lateral mesoderm cells (ii). Alternatively, hPSC-derived lateral mesoderm (day 3) was further differentiated into artery endothelial cells (day 4) in the presence or absence of retinoid pathway agonist TTNPB, followed by qPCR or flow cytometry of artery endothelial cells (iii-vi). qPCR data were normalized to the sample with the highest expression in this experiment.

*Histograms depict the mean±SEM. *P<0.05, **P<0.01, n.s. = not significant.*

Supplemental Figure 6

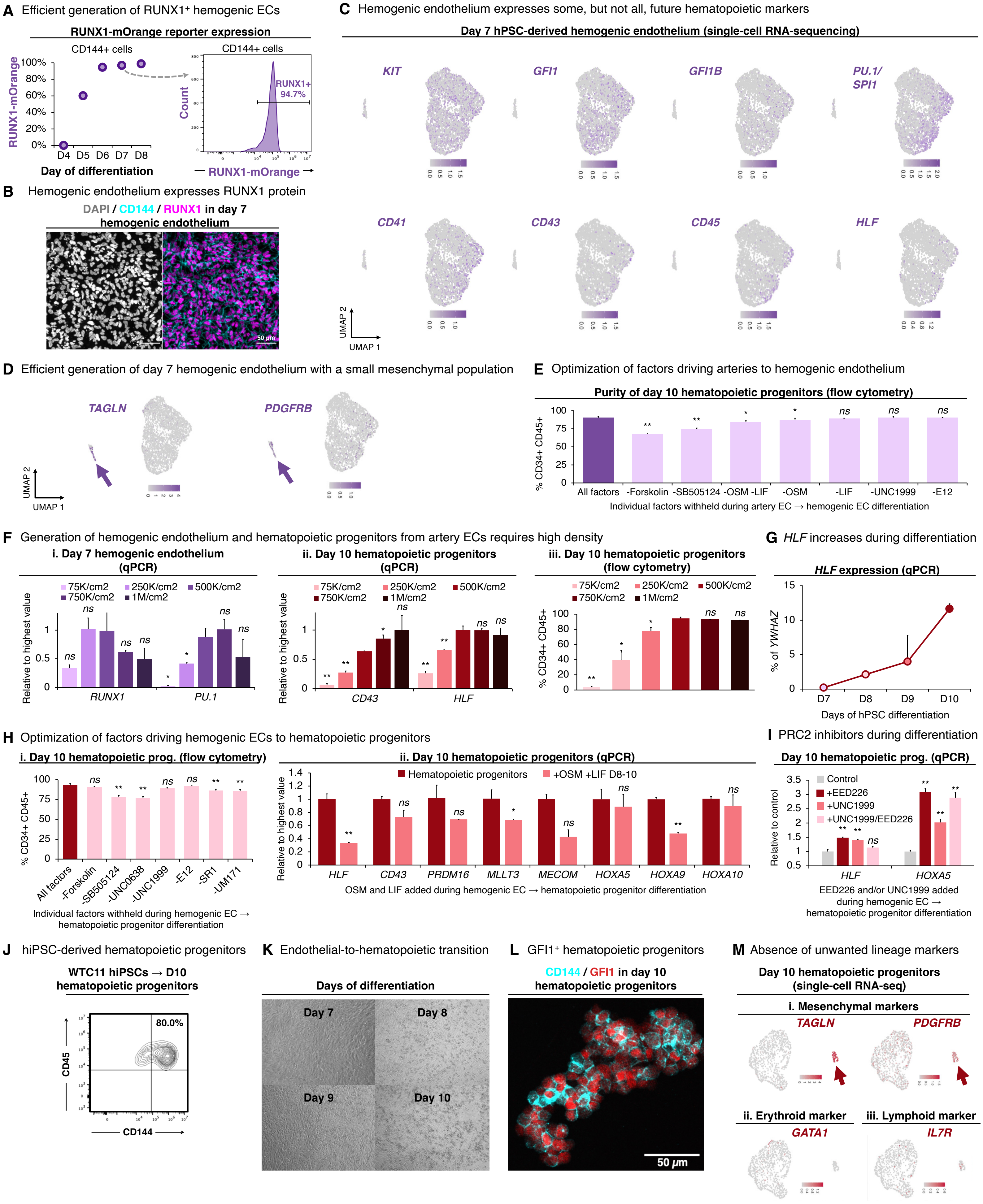

Supplementary Figure 6: Optimization of hPSC differentiation into hemogenic ECs and *HOXA*+ *HLF*+ hematopoietic progenitors, related to Figure 5.

A) *RUNX1-mOrange* hPSCs[6] were differentiated into day-4 artery ECs, which were then treated with hemogenic EC-inducing signals for 1-4 days (until days 5-8 of hPSC differentiation, respectively). Flow cytometry was performed to assess RUNX1-mOrange reporter expression, pre-gated on CD144+ ECs.

B) Immunostaining of day-7 hPSC-derived hemogenic ECs for RUNX1 and CD144 (VE-CADHERIN), with DAPI nuclear counterstain.

C) scRNA-seq of day-7 hPSC-derived hemogenic ECs, showing EC marker expression.

D) scRNA-seq of day-7 hPSC-derived hemogenic ECs, showing a small subset of cells expressing mesenchymal markers.

E) Day 4 hPSC-derived artery ECs were differentiated for 3 days into hemogenic ECs, or alternatively, individual differentiation factors were individually withheld during hemogenic EC induction, followed by continued 3-day differentiation into day 10 hematopoietic progenitors, which were analyzed by flow cytometry. Individual differentiation factors were withheld only during hemogenic EC induction; at the next step, the complete set of hematopoietic progenitor induction signals was used.

F) Day 4 hPSC-derived artery ECs were dissociated and re-plated at the indicated densities, followed by differentiation into day 7 hemogenic ECs (i) or day 10 hematopoietic progenitors (ii,iii), which were analyzed by qPCR or flow cytometry. qPCR data were normalized to the sample with the highest expression in this experiment.

G) Day 7 hPSC-derived hemogenic ECs were differentiated into hematopoietic progenitors for 0-3 days, and qPCR was performed on days 7-10 of differentiation. qPCR data shown relative to reference gene *YWHAZ* (i.e., *YWHAZ* expression level = 100%).

H) Day 7 hPSC-derived hemogenic ECs were differentiated for 3 days into hematopoietic progenitors, or alternatively, individual differentiation factors were individually withheld during hematopoietic progenitor induction, followed by flow cytometry (i). Alternatively, during the 3 days of hematopoietic progenitor differentiation, OSM and LIF were added, and qPCR of the resultant hematopoietic progenitors was performed (ii).

I) Day 7 hPSC-derived hemogenic ECs were differentiated for 3 days into hematopoietic progenitors in the absence of PRC2 inhibitors, or alternatively, in the presence of PRC2 inhibitors UNC1999 and/or EED226. qPCR was performed on day 10 hPSC-derived hematopoietic progenitors. qPCR data were normalized to the negative control (absence of PRC2 inhibitors).

J) WTC11 hiPSCs were differentiated into day-10 hematopoietic progenitors, followed by flow cytometry.

K) Phase contrast microscope images of day-7 hPSC-derived hemogenic ECs progressively differentiating into day 10 hematopoietic progenitors, revealing gradual emergence of rounded, semi-adherent cells.

L) Immunostaining of day-10 hPSC-derived hematopoietic progenitors for GFI1 and CD144 (VE-CADHERIN).

M) scRNA-seq of day-10 hPSC-derived hematopoietic progenitors, showing (1) mesenchymal marker expression in a small subset of non-hematopoietic cells and (2) minimal expression of downstream hematopoietic markers that are expressed in lymphoid and erythroid progenitors within the human embryo AGM[7,8].

*Histograms and line graphs depict the mean±SEM. *P<0.05, **P<0.01, n.s. = not significant. Scale: 50 μm.*

Supplemental Figure 7

A hPSC-derived day 10 hematopoietic progenitors express HSC signature genes

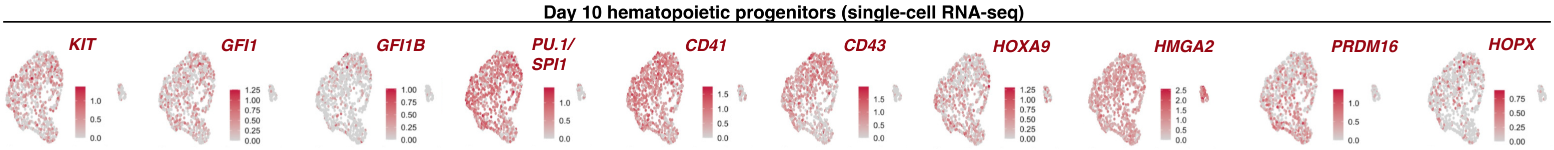

B Single-cell RNA-sequencing comparison of hPSC-derived hematopoietic progenitors from the current study versus published protocols

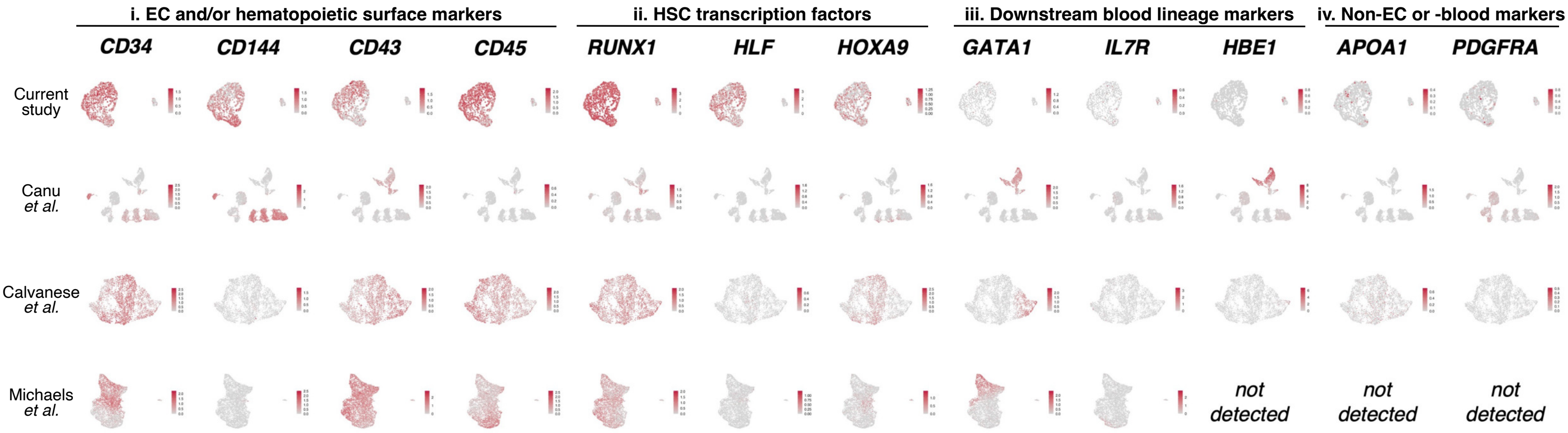

C hPSC-derived hematopoietic progenitors express HSC signature genes at levels comparable to human AGM HSCs and human cord blood HSCs

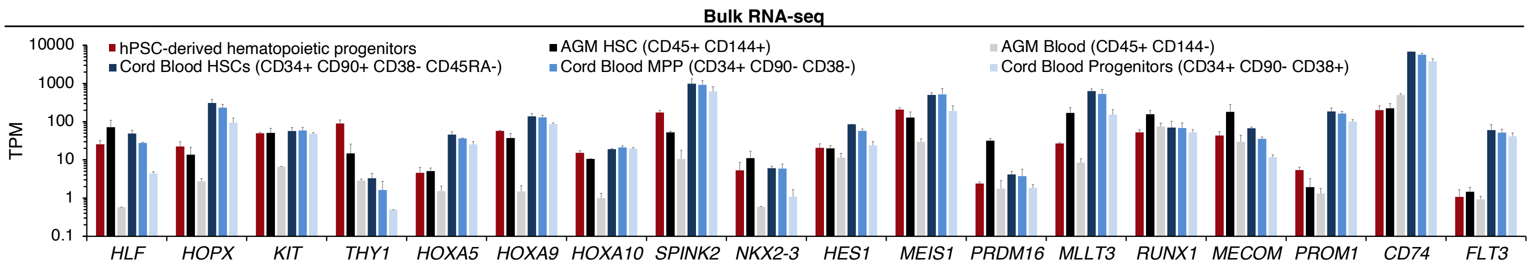

D Single-cell RNA-sequencing comparison of hPSC-derived hematopoietic progenitors and human CS15 AGM HSCs

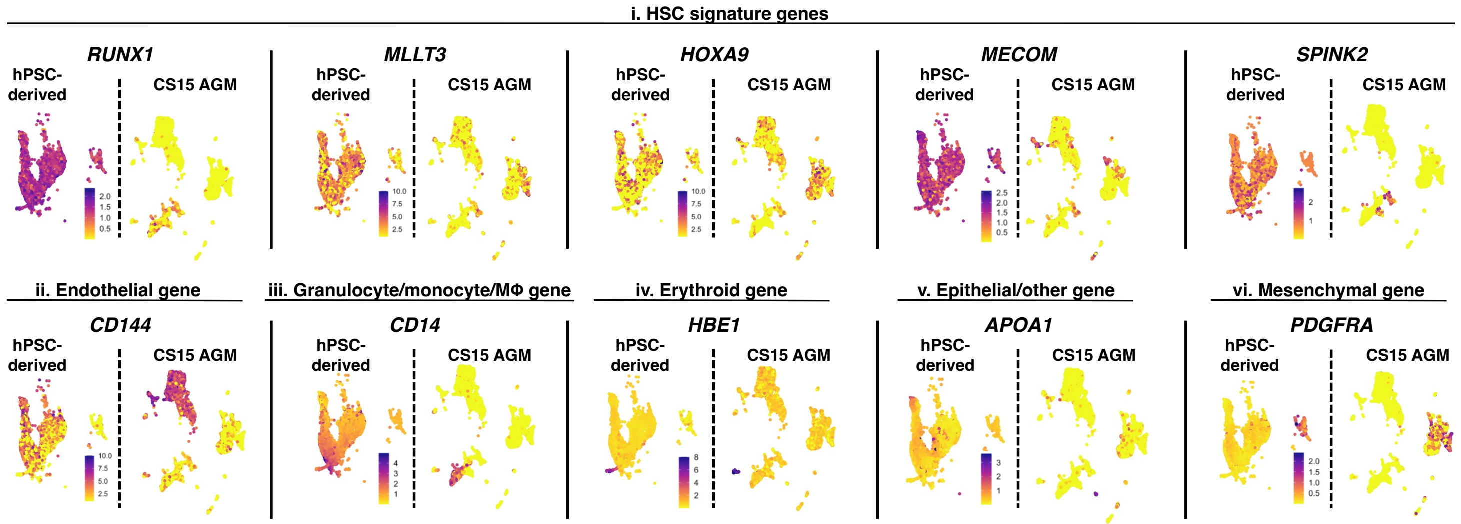

E Hemoglobin in hPSC-derived erythroid cells

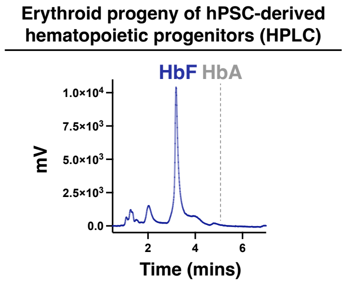

F Transplantation of AML-hiPSC derived hematopoietic progenitors

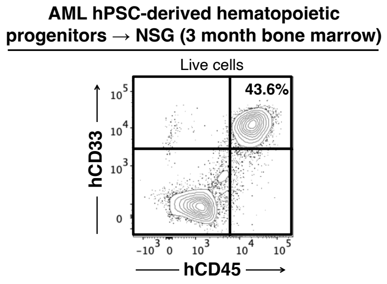

G CD34+ cord blood HSPCs engraft *in vivo*

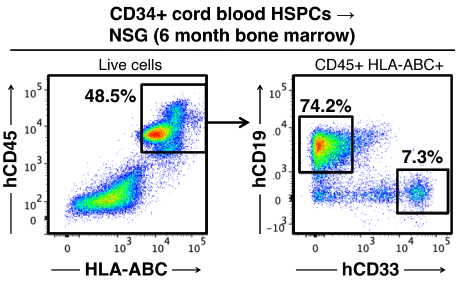

Supplementary Figure 7: Transcriptional and functional analyses of hPSC-derived *HLF*+ *HOXA*+ hematopoietic progenitors, related to Figures 6-7.

A) scRNA-seq of day 10 hPSC-derived hematopoietic progenitors. The entire day 10 population was analyzed, without preselecting for any cell subset.

B) scRNA-seq of hPSC-derived day-10 hematopoietic progenitors (this study) compared with published scRNA-seq profiles of hPSC-derived hematopoietic progenitors produced from three other differentiation protocols[8-10]. For all differentiation protocols, the entire cell population was analyzed, without preselecting for any cell subset. For our differentiation protocol ("current study"; top row), some scRNA-seq plots are identical to those shown in **Fig. 6A** and **Fig. S7A**, but are reproduced again here in order to compare the outcomes of various differentiation protocols.

C) Bulk-population RNA-seq of day 10 hPSC-derived hematopoietic progenitors, compared with FACS-purified human cord blood HSCs (CD34+ CD90+ CD38- CD45RA- Lineage-), human cord blood MPPs (CD34+ CD90- CD38- Lineage-), and human cord blood downstream progenitors (CD34+ CD90- CD38+ Lineage-), as well as published RNA-seq profiles of FACS-purified CD45+ CD144+ HSCs vs. CD45+ CD144- non-HSCs isolated from the aorta-gonad-mesonephros (AGM) region of Carnegie Stage 15/16 human embryos[11]. All gene expression shown in transcript per million (TPM) units.

D) scRNA-seq of day 10 hPSC-derived hematopoietic progenitors, compared with published scRNA-seq profiles of the Carnegie Stage 15 human embryo AGM[8].

E) hPSC-derived day-10 hematopoietic progenitors were differentiated into erythroid cells, followed by high-performance liquid chromatography (HPLC) to assess expression of fetal (HbF) or adult (HbA) hemoglobin proteins.

F) iSU223n AML patient-derived hiPSCs[12,13] were differentiated into day 10 hematopoietic progenitors, which were then transplanted into NSG mice. Flow cytometry was performed of the bone marrow 3 months post-transplantation.

G) CD34+ human cord blood HSPCs were intrafemorally transplanted into NSG mice, followed by flow cytometry of the bone marrow 6 months post-transplantation. Positive control for **Fig. 7I**.

Histograms depict the mean±SEM.

Table S4: Quantitative PCR primers used this study, related to STAR Methods.
All quantitative PCR (qPCR) primer sequences target human genes.

Primers	Forward	Reverse
BRACHYURY (*TBXT*)	TGCTTCCCTGAGACCCAGTT	GATCACTTCTTTCCTTTGCATCAAG
EOMES	CAACATAAACGGACTCAATCCCA	ACCACCTCTACGAACACATTGT
HHEX	CACCCGACGCCCTTTTACAT	GAAGGCTGGATGGATCGGC
FOXA2	GGGAGCGGTGAAGATGGA	TCATGTTGCTCACGGAGGAGTA
FOXB1	CTTTAAGATCCGAGCAGTCCGCC	GCTCAGCGGCAGCATCTTCT
MESP1	GAAGTGGTTCCTTGGCAGAC	TCCTGCTTGCCTCAAAGTGT
CDX2	GGGCTCTCTGAGAGGCAGGT	CCTTTGCTCTGCGGTTCTG
CDX4	AGTCTGGGGCTCACCCTAC	CTGTGCCCATTGTACTAGACG
CD144 (CDH5/VE-CADHERIN)	AACGAGCAGGGCGAGTTCACCTTC	TAGGTGACCAGCTGCTCGTGGATC
CD31 (PECAM1)	AACAGTGTTGACATGAAGAGCC	TGTAAAACAGCACGTCATCCTT
CD43 (SPN)	CACTTCAATAACAAGTGACCCTAA GG	TGGTAGGTTGTTGGCTCAGGTA
CD45 (PTPRC)	ACCACAAGTTTACTAACGCAAGT	TTTGAGGGGGATTCCAGGTAAT
DLL4	GTCTCCACGCCGGTATTGG	CAGGTGAAATTGAAGGGCAGT
HLF	CTGGGGCCTACCTTATGGGA	GGGGAATGCCATTTTCTGACA
HOXA1	CGTGAGAAGGAGGGTCTCTTG	GTGGGAGGTAGTCAGAGTGTC
HOXA2	CAGAACCGGAGGATGAAGCA	ACGCTAAGGGCTTGCTCAAA
HOXA3	AGCAGCTCCAGCTCAGGCGAAA	TGGCGCTCAGTGAGGTTCAG
HOXA4	CGTGGTGTACCCCTGGATGAAG	TATAACTGGGGTTAACGGCGCT
HOXA5	AAACTGTGACTCCAAGCGGT	GAGCCACTTCCAGAGTTCGT
HOXA7	AGGAGTTCCACTTCAACCGC	CAGTCGGACCTTCGTCCTTAT
HOXA9	TTGCACCAGACGAACAGTGA	GCCCAATGGCGGTTTCATAG
HOXA10	CTGGTTTCAGAACCGCAGGA	AGATGTAACGGCCCAGGAGA
MECOM (EVI1)	TATCCACGAAGAACGGCAATATC	CATGGAAACTTTTGGTGATCTGC
MIXL1	GGTACCCCGACATCCACTTG	TAATCTCCGGCCTAGCCAAA
MLLT3	TTTGTGGAGAAAGTCGTCTTCC	GAGGTGATTCACTGGTGGATG

PRDM16	GGCTGCTTCTGGACTCAAGG	CCCGGTTGGGCTCATACATA
PU.1 (SPI1)	GTGCCCTATGACACGGATCTA	AGTCCCAGTAATGGTCGCTAT
RUNX1	AGAACCTCGAAGACATCGGC	GGCTGAGGGTTAAAGGCAGTG
SOX17	CGCACGGAATTTGAACAGTA	GGATCAGGGACCTGTCACAC
YWHAZ	GAGCTGGTTCAGAAGGCCAAAC	CCTTGCTCAGTTACAGACTTCATGCA

Supplementary References

1. Qiu, C., Martin, B.K., Welsh, I.C., Daza, R.M., Le, T.M., Huang, X., Nichols, E.K., Taylor, M.L., Fulton, O., O'Day, D.R., et al. (2024). A single-cell time-lapse of mouse prenatal development from gastrula to birth. Nature. 10.1038/s41586-024-07069-w.
2. Pijuan Sala, B., Griffiths, J.A., Guibentif, C., Hiscock, T.W., Jawaid, W., Calero-Nieto, F.J., Mulas, C., Ibarra-Soria, X., Tyser, R.C.V., Ho, D.L.L., et al. (2019). A single-cell molecular map of mouse gastrulation and early organogenesis. Nature, 1-25. 10.1038/s41586-019-0933-9.
3. Baron, C.S., Kester, L., Klaus, A., Boisset, J.C., Thambyrajah, R., Yvernogeau, L., Kouskoff, V., Lacaud, G., van Oudenaarden, A., and Robin, C. (2018). Single-cell transcriptomics reveal the dynamic of haematopoietic stem cell production in the aorta. Nat Commun *9*, 2517. 10.1038/s41467-018-04893-3.
4. Beyer, S., Kelly, R.G., and Miquerol, L. (2011). Inducible Cx40-Cre expression in the cardiac conduction system and arterial endothelial cells. Genesis *49*, 83-91. 10.1002/dvg.20687.
5. Liu, Z., Gu, Y., Chakarov, S., Bleriot, C., Kwok, I., Chen, X., Shin, A., Huang, W., Dress, R.J., Dutertre, C.A., et al. (2019). Fate Mapping via Ms4a3-Expression History Traces Monocyte-Derived Cells. Cell *178*, 1509-1525 e1519. 10.1016/j.cell.2019.08.009.
6. Ikeda, K., Uchida, N., Nishimura, T., White, J., Martin, R.M., Nakauchi, H., Sebastiano, V., Weinberg, K.I., and Porteus, M.H. (2018). Efficient scarless genome editing in human pluripotent stem cells. Nature Methods *15*, 1045-1047. 10.1038/s41592-018-0212-y.
7. Zeng, Y., He, J., Bai, Z., Li, Z., Gong, Y., Liu, C., Ni, Y., Du, J., Ma, C., Bian, L., et al. (2019). Tracing the first hematopoietic stem cell generation in human embryo by single-cell RNA sequencing. Cell Research, 1-14. 10.1038/s41422-019-0228-6.
8. Calvanese, V., Capellera-Garcia, S., Ma, F., Fares, I., Liebscher, S., Ng, E.S., Ekstrand, S., Aguade-Gorgorio, J., Vavilina, A., Lefaudeux, D., et al. (2022). Mapping human haematopoietic stem cells from haemogenic endothelium to birth. Nature *604*, 534-540. 10.1038/s41586-022-04571-x.
9. Canu, G., Athanasiadis, E., Grandy, R.A., Garcia-Bernardo, J., Strzelecka, P.M., Vallier, L., Ortmann, D., and Cvejic, A. (2020). Analysis of endothelial-to-haematopoietic transition at the single cell level identifies cell cycle regulation as a driver of differentiation. Genome Biology, 1-28. 10.1186/s13059-020-02058-4.
10. Michaels, Y.S., Edgar, J.M., Major, M.C., Castle, E.L., Zimmerman, C., Yin, T., Hagner, A., Lau, C., Hsu, H.H., Ibanez-Rios, M.I., et al. (2022). DLL4 and VCAM1 enhance the emergence of T cell-competent hematopoietic progenitors from human pluripotent stem cells. Sci Adv *8*, eabn5522. 10.1126/sciadv.abn5522.
11. Crosse, E.I., Gordon-Keylock, S., Rybtsov, S., Binagui-Casas, A., Felchle, H., Nnadi, N.C., Kirschner, K., Chandra, T., Tamagno, S., Webb, D.J., et al. (2020). Multi-layered Spatial Transcriptomics Identify Secretory Factors Promoting Human Hematopoietic Stem Cell Development. Cell Stem Cell *27*, 822-839 e828. 10.1016/j.stem.2020.08.004.
12. Chao, M.P., Gentles, A.J., Chatterjee, S., Lan, F., Reinisch, A., Corces, M.R., Xavy, S., Shen, J., Haag, D., Chanda, S., et al. (2017). Human AML-iPSCs Reacquire Leukemic Properties after Differentiation and Model Clonal Variation of Disease. Cell Stem Cell *20*, 329-344 e327. 10.1016/j.stem.2016.11.018.
13. Nishimura, T., Xu, H., Iwasaki, M., Karigane, D., Saavedra, B., Takahashi, Y., Suchy, F.P., Monobe, S., Martin, R.M., Ohtaka, M., et al. (2019). Sufficiency for inducible Caspase-9 safety switch in human pluripotent stem cells and disease cells. Gene Ther *27*, 525-534. 10.1038/s41434-020-0179-z.
